# Potential repurposing of four FDA approved compounds with antiplasmodial activity identified through proteome scale computational drug discovery and in vitro assay

**DOI:** 10.1038/s41598-020-80722-2

**Published:** 2021-01-14

**Authors:** Bakary N’tji Diallo, Tarryn Swart, Heinrich C. Hoppe, Özlem Tastan Bishop, Kevin Lobb

**Affiliations:** 1 Research Unit in Bioinformatics (RUBi), Department of Biochemistry and Microbiology, Rhodes University, Grahamstown, 6140 South Africa; 2 Department of Chemistry, Rhodes University, Grahamstown, 6140 South Africa; 3 Department of Biochemistry and Microbiology, Rhodes University, Grahamstown, 6140 South Africa

**Keywords:** High-throughput screening, Drug discovery and development, Virtual screening

## Abstract

Malaria elimination can benefit from time and cost-efficient approaches for antimalarials such as drug repurposing. In this work, 796 DrugBank compounds were screened against 36 *Plasmodium falciparum* targets using QuickVina-W. Hits were selected after rescoring using GRaph Interaction Matching (GRIM) and ligand efficiency metrics: surface efficiency index (SEI), binding efficiency index (BEI) and lipophilic efficiency (LipE). They were further evaluated in Molecular dynamics (MD). Twenty-five protein–ligand complexes were finally retained from the 28,656 (36 × 796) dockings. Hit GRIM scores (0.58 to 0.78) showed their molecular interaction similarity to co-crystallized ligands. Minimum LipE (3), SEI (23) and BEI (7) were in at least acceptable thresholds for hits. Binding energies ranged from −6 to −11 kcal/mol. Ligands showed stability in MD simulation with good hydrogen bonding and favorable protein–ligand interactions energy (the poorest being −140.12 kcal/mol). In vitro testing showed 4 active compounds with two having IC_50_ values in the single-digit μM range.

## Introduction

In the 2019 World Malaria Report, the WHO African Region accounted for 94% of malaria cases in which 99.7% are caused by *P. falciparum*. The report showed a trend towards malaria elimination in more countries and declines in cases and mortality thanks to extensive efforts to fight the disease. However, a concerning stalling trend in malarial cases and deaths has been observed in some regions^1^. Moreover, there is no effective vaccine available, and reduction of funding such efforts could be disastrous in the long term^1^. Malaria remains a major health concern with parasites frequently developing resistance to the different drugs introduced to treat it. This threatens the current recommended artemisinin-based combination therapy (ACT) which at present is still effective^1^. Compounds having different mechanisms of action are required to address the resistance problem^2^, but to stay ahead of the resistance curve it is necessary to continuously develop new antimalarials^3^.

During the last few decades, the high attrition rate of existing drugs renewed interest in phenotypic screening and drug repurposing as a new alternative approach in drug discovery^4^. Drug repurposing is a time and cost-effective strategy with reduced failure risk for drug development. Also known as “drug reprofiling”, “drug repositioning”, “therapeutic switching” or “drug re-tasking”, it consists in using existing drugs (e.g. an approved drug or investigational drugs) to treat different diseases than their original indications^5^. As known drugs, they often already have in vitro and in vivo screening data, chemical optimization, bulk manufacturing, formulation data, toxicity and pharmacokinetic information, thus, presenting major cost and time advantage over the classical drug development pipeline^4,6^. Therefore, many pharmaceutical companies have included re-purposing strategies in their pipelines. In the context of drug resistance in malaria, drug repurposing strategies can accelerate antimalarial development to stay ahead of the resistance curve^7^. Some successful examples of drug repositioning include thalidomide: First causing phocomelia in children born from women taking it, it was later successfully repurposed to treat erythema nodosum leprosum and in combination with dexamethasone to treat multiple myeloma. Sales of the drug reached 224 US dollars in 2003. Sildenafil (Viagra) developed by Pfizer was first ineffective in treating angina pectoris and later found to be effective in the treatment of erectile dysfunction^8^. As a third example, eflornithine, an ornithine decarboxylase inhibitor, was developed as an anticancer agent but was found to be effective for trypanosomiasis and later approved for slowing the growth of unwanted facial hair^9^. Further examples of successfully re-purposed drugs include wellbutrin, minoxidil, duloxetine and zidovudine^10^.

The purpose of this study was to explore the re-purposing of FDA approved drugs for malaria treatment. The approach used here involved the combination of different metrics to identify promising selective malarial target-approved drug pairs.

## Results and discussion

### Workflow: Pose accuracy and scoring bias

**Figure 1:**
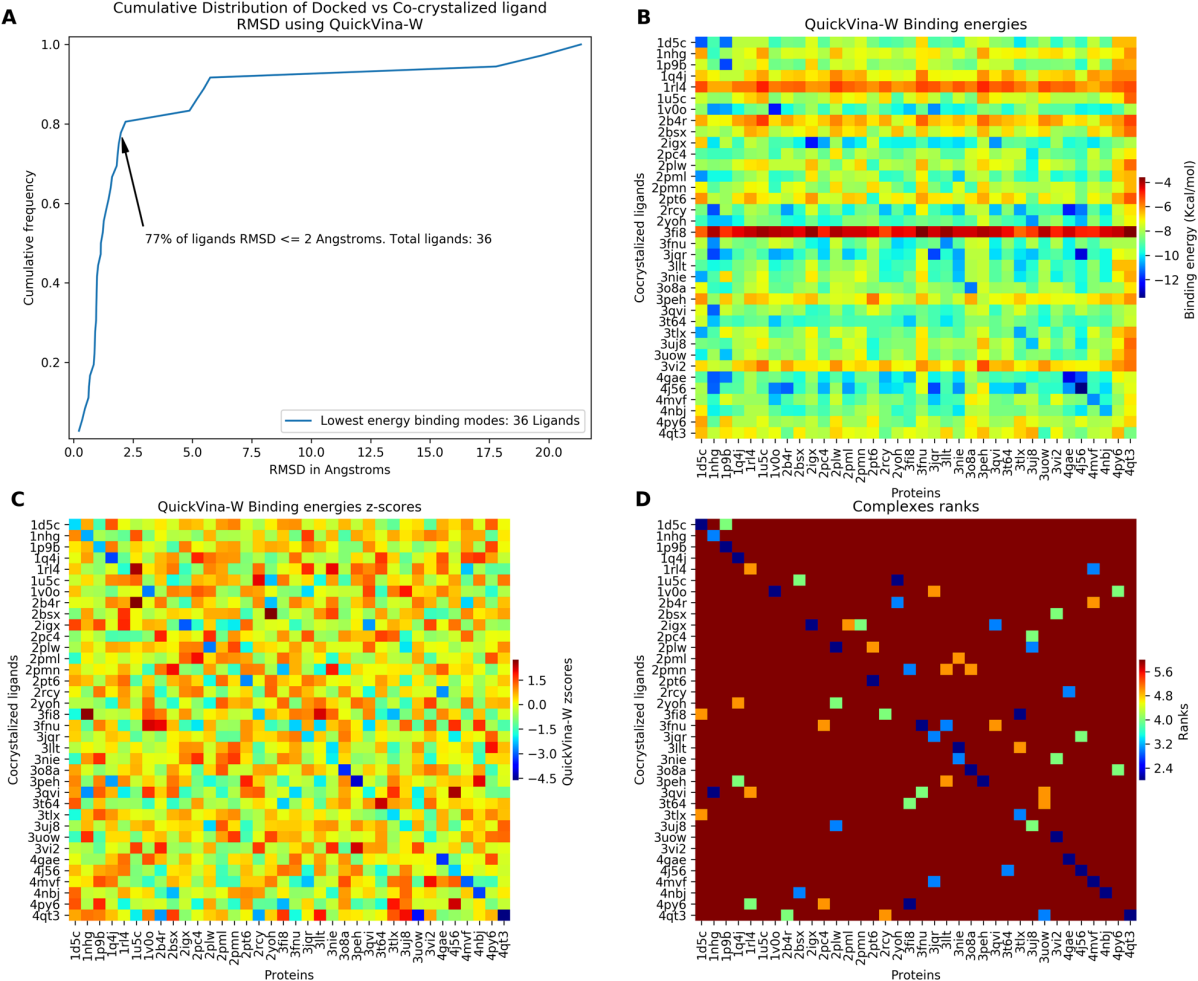
Docking validation and scores transformations. (**A**) The cumulative distribution of the RMSD (docked vs co-crystallized poses of the ligands). (**B**) Raw binding energies. (**C**) Standardized values; (**D**) Rank transformed values (only complexes with a rank value ≤ 6 are shown for clarity. On the heatmaps, rows (ligands) and columns (proteins) are alphabetically ordered. Heatmaps were generated using Seaborn version 0.9^85^.

First, co-crystallized ligands were docked to the different proteins. Figure 1 presents docked poses RMSD, binding energies, standardized and rank transformed values. On the heatmaps, cells on the diagonal represent pairs of proteins and their respective co-crystallized ligands (redocking). As expected redocked ligands tended to have the lowest calculated energy values.

From the docking experiment, 77% of ligands were docked accurately with an RMSD <  = 2 Å when considering only poses with the lowest RMSD (Fig. 1A). Pose accuracy in benchmarking studies showed similar proportions of accurate pose^11,12^. Co-crystallized ligands in the following proteins docked with RMSD greater than 2 Å: 1u5c (4.8 Å), 2b4r (21.3 Å), 2pc4 (5.7 Å), 2rcy (2.1 Å), 3jqr (5.4 Å), 3qvi (19.7 Å), 3uow (17.7 Å), 3vi2 (5.1 Å). While some of these included ligands still bound in the active site vicinity, co-crystallized ligands in 2b4r, 3qvi and 3uow showed RMSD deviations greater than 6 Å, 21 Å, 19 Å and 17 Å, respectively. Further structural analysis to investigate alternate conformations, protonation states, water-mediated interactions, flips of histidine, glycine or asparagine, missing residues, especially in the active site and other factors impacting docking, was done on these structures. In 2b4r structure, MolProbity^13^ indicated clear evidence of flip for ASN185, an active site residue. The co-crystallized ligand (AES) was redocked with an RMSD of 1.23 Å with the flipped ASN185. This might be the residue accurate conformation. Hence, current structures in sc-PDB may be further improved for docking with an analysis for potential residue flips. It is also noteworthy that AES was putatively assigned to an unexpected extra electron density^14^. Histo-aspartic protease (3qvi) was found to function at low pH (5.5)^15^. However, the co-crystallized ligand pose was not reproduced using the protonated structure at that pH^16^. Our structural investigation did not improve the docking for 3uow. MolProbity^13^ indicated GLN476 (chain A) was flipped. However, the docked ligand on the flipped GLN476 in the receptor did not reproduce the correct pose. An explanation for 3uow may be the rigid receptors. The enzyme guanosine 5′-monophosphate synthetase (3uow) is known to undergo a significant conformational change upon binding of XMP^17, 18^. An induced-fit docking approach or an approach with flexible residues may be a better alternative. Hence, despite the overall success in redocking with the majority of the structures, these three cases may be a limitation.

On binding energies, standardization of scores alleviated protein and ligand related biases. The difference of the mean of binding energies of ligands screened on 1nhg and 4qt3 was 2.61 kcal/mol (-9.11 kcal/mol and −6.50 kcal/mol respectively) (Fig. 1B,C). This may be explained by the buried active site in 1nhg, as compared to the more solvent-exposed active site of 4qt3. The standardization process reduced this inter protein noise by centering the mean of binding energies on each protein at zero. A similar phenomenon is observed when viewing and comparing the binding of pairs of ligands across the whole dataset. Co-crystallized ligands in 3fi8 and 1rl4, 2-aminoethyl dihydrogen phosphate and (2R)-2- [(hydroxy–amino)methyl]hexanoic acid have low molecular weights 141 Da and 189 Da respectively which may explain their low scores across all proteins (Fig. 1B). Indeed, vina scoring function has a ligand size-related bias^20^. By contrast, the co-crystallized ligand from 4j56, flavin-adenine dinucleotide, has a high molecular weight (785 Da) which may explain its high promiscuity (Fig. 1B).

As key findings from the workflow evaluation, 77% of co-crystallized ligands poses were reproduced. On the binding energies, scores standardization improved protein and ligand related biases.

**Figure 2:**
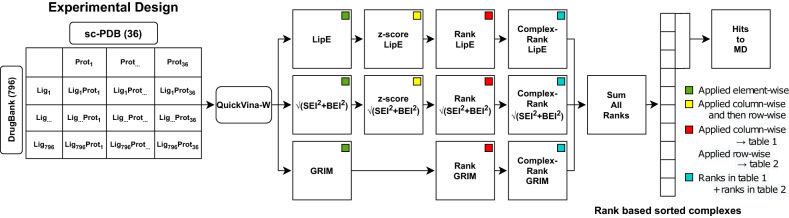
Virtual screening workflow. Square boxes represent tables of protein–ligand systems as described in the experimental design. Values in the table are given by the metric in the box center. Colors code the type of operation applied on each table.

The above workflow was extended to include ligand efficiency metrics and GRIM. The overall screening workflow including docking, scoring, ranking and molecular dynamics is represented in Fig. 2 and further detailed in the methods Sect. 796 drugs from DrugBank^21^ were docked against 36 *Plasmodium falciparum* protein structures. LipE, BEI and SEI were calculated for each ligand most energetically favorable pose. All poses were scored using the GRIM tool to obtain the Grscore and the pose with the best Grscore was maintained in the GRIM ranking scheme. LipE, SEI, BEI scores were standardized through the z-score across the proteins and then the ligands. All scores were then transformed to their ranks and a complex rank (sum of the ligand and protein ranks) is attributed to each complex as described above. A protein rank is its rank respective to a ligand when compared to other proteins. Complex ranks are finally summed and ranked. From the ranked list, 36 complexes were selected (each protein and ligand being unique) for further analysis of their docked poses and structure stability in MD. Finally, 25 stable complexes were maintained.

### Screening the DrugBank subset against *P. falciparum* structures

#### Top predicted complexes

The workflow in Fig. 2 was applied to the set of DrugBank^21^ compounds and the same set of targets. From the screening, 36 complexes, a ligand for each structure, preferentially the pose having the best Grscore if its binding energy was in a range of ± 2 kcal/mol of the lowest binding energy. Otherwise, the best Vina pose is used as it then has significantly better energy given Vina standard error of 2.85 kcal/mol^22^. For a better drug target space coverage of *P. falciparum*, a hit compound was selected for each drug target. After further visual analysis and MD evaluation, 25 complexes, 25 hits compounds for 25 unique targets were retained based on their stability during the simulation (Supplementary Table S2).

Regarding their ligand efficiencies, identified hits had acceptable LipE and BEI values. The minimum values LipE and BEI were 3, 23 respectively. Indeed, ideal values for LipE, BEI and SEI for small molecules in screening are respectively 3, 27, 18^23^. SEI values were consistently low. The minimum value was 7 while the highest value was 33. The average SEI value was 12, well within the lower limit threshold for SEI (15) defined as a good starting point by Kumar et al.^24^. Abiraterone, a hydrophobic compound with a cLogP of 5.3 and PSA of 33 Å^2^ had the highest SEI value. Dianhydrosorbitol 2,5-dinitrate, gemifloxacin, temozolomide, triamcinolone, pirbuterol, terazosin, tenoxicam, abacavir and sitaxentan all showed SEI < 10.

**Figure 3:**
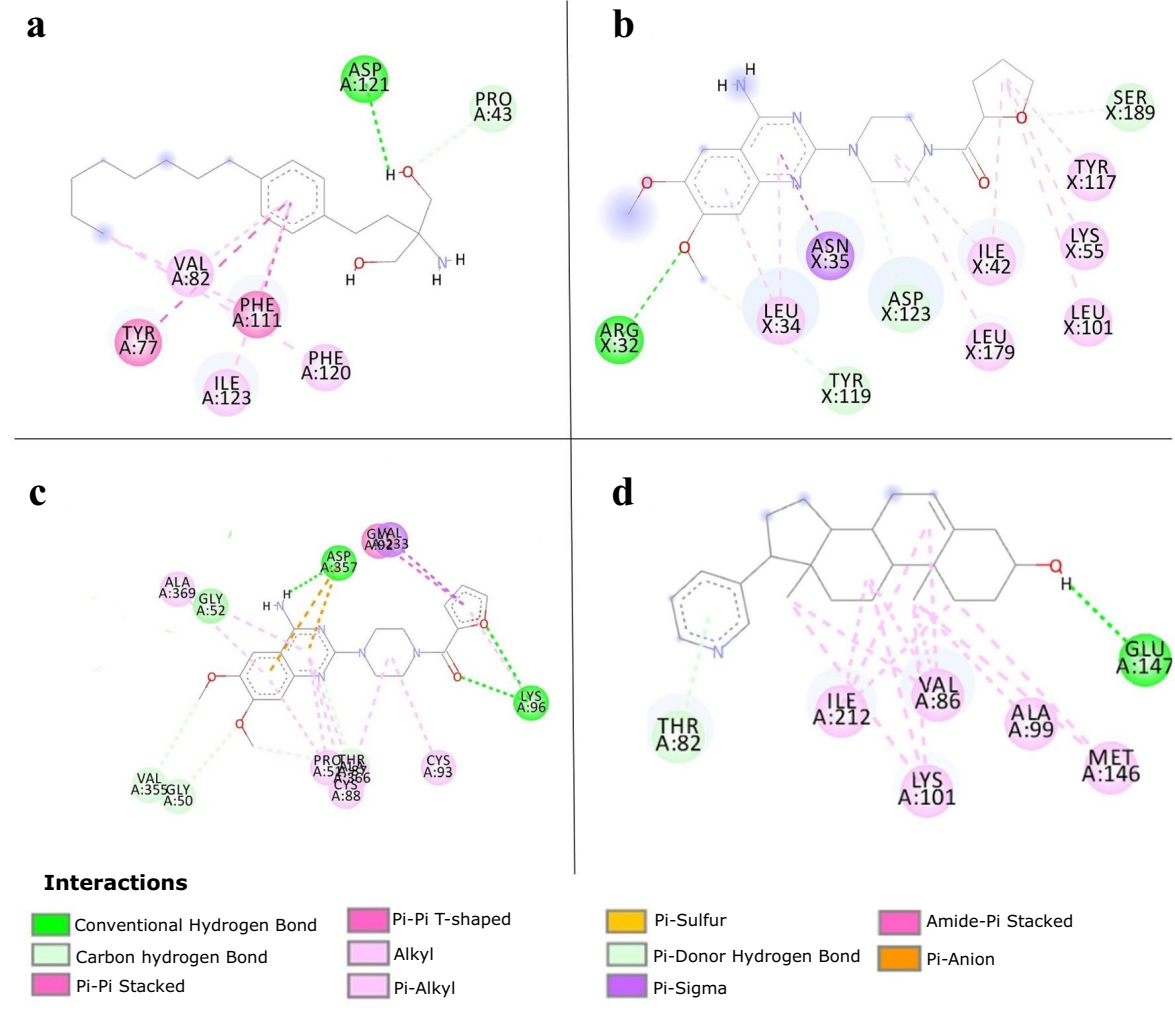
2D plot of intermolecular interactions depicted using Discovery Studio Visualizer 2017 R2. (**A**) Fingolimod and Plasmepsin 2, (**B**) Terazosin PfPK7 and (**C**) Prazosin and PfTrxR (**D**) Abiraterone and PfCDPK2. Dashed lines represent the different interactions and their color the interaction type. Colored circles represent residues with their three letter code, chain identifier and residue number.

On the other hand, with respect to their molecular interactions, hits and co-crystallized ligands showed similar interaction profiles supported by their high Grscore. All the selected complexes ligands showed a Grscore greater or equal to 0.58. Hit structures (Fig. 3) were compared with those of known inhibitors and current antimalarials. Most hits presented new scaffolds with good potential for scaffold modification in their respective binding sites. In the comparison of the current hits to the set of FDA-approved antimalarials, only terazosin (the hit for 2pml) and the FDA-approved primaquine had a similarity above 0.5 (0.52). However, this value is still too low to infer significant structural analogy. Further, hit scaffolds were also compared with those of the corresponding target known inhibitors in ChEMBL^25^ (if present) and to those of known antimalarials. The targeted protein FASTA sequence for known antimalarials was searched in ChEMBL^25^ using BLAST to find the corresponding protein. In most cases, the known inhibitors showed low similarity to the hit compound.

These differences between the hits and the co-crystallized ligands in terms of structure is in contrast with the high Grscores of the hits. However, Grscores do not imply ligand similarity, but rather similarity in molecular interactions. Of course, the GRIM score is not ligand centric nor target centric but focuses on the similarity of molecular interactions^26^. Nevertheless, some moieties and subgroups from hits had comparable binding modes with the co-crystallized ligands. For example, abacavir and sinefungin both have a purine moiety similarly interacting with the target 3uj8 (see Supplementary Fig. S15).

In addition, many compounds have appropriate molecular properties for scaffold modification (low molecular weight and logP). Furthermore, in their binding modes, some compounds showed good potential for extension within the binding site.

Table 1 presents the four hits confirmed through in vitro experiments, their IC_50_, binding energies, Grscore, and predicted targets. The section below describes the confirmed hits through in vitro assays. All other complexes selected after docking are described in the Supplementary Data (Sect. 1.4). A detailed analysis was performed on the four complexes: not only binding poses and molecular interactions but also their potential action on the parasite life cycle. All drug indication information was obtained from DrugBank^21^^.^

**Table 1:** Active compounds with their targets names, PDB IDs, QuickVina-W binding energies, GRIM Grscores and IC_50_. All other systems are in Supplementary Table S2. Structures’ drawings were obtained from DrugBank^86^. Structures were depicted with Open Babel^87^.

Names	IC_50_ (µM)	Structures	Predicted target and PDB ID	Binding Energy (kcal/mol)	Grscore	Mean PLIE^1^ (kcal/mol)
2igxFingolimod	2.21		Plasmepsin 2 (2igx)	−7.9	0.79	−177.18
4mvfAbiraterone	3.37		Calcium-dependent protein kinase 2 (4mvf)	−11.0	0.69	−166.17
4j56Prazosin	16.67		Thioredoxin reductase 2 (4j56)	−11.4	0.71	−331.42
Terazosin	34.72		PfPK7 (2pml)	−9.0	0.62	−190.83

^1^PLIE: Protein–ligand interaction energy.

*Plasmepsin 2 (PDB ID: 2igx)—Fingolimod (DB08868)* Plasmepsin 2 belongs to a family of aspartic proteinases involved in haemoglobin degradation for the parasite, and the crystal structure 2igx is co-crystallized with a potent achiral inhibitor (HET CODE: A1T)^27^. The compound fingolimod is known to modulate sphingosine 1-phosphate receptor activity and is indicated for the treatment of relapsing–remitting multiple sclerosis. This compound binds to Plasmepsin 2 at the A1T binding site, buried in a hydrophobic pocket, interacting with ASP121 though one of its hydroxyl groups (Fig. 3A and Supplementary Figure S38a) forming a hydrogen bond deep within the pocket. Fitting within that pocket has been observed to be crucial for high-affinity inhibitors^27^. Fingolimod has its polar groups (hydroxyls and amino) fitting in the depth of the hydrophobic pocket which may have associated energetic cost^28^. By contrast, A1T has a characteristic long aliphatic chain. Through its aromatic ring, fingolimod makes hydrophobic contacts with TYR77, PHE111, ILE123 and VAL82, and has Pi-Pi interactions with TYR77 and PHE111 (Fig. 3A). The carbon chain engages in alkyl contacts with PHE120 and PHE111 (Fig. 3A). Unlike A1T, fingolimod which presents a different scaffold (Tanimoto similarity 0.22 with A1T), and further does not show a large expansion in the vast trench area outside the pocket, though this region may offer an ideal possibility for scaffold growing. The phenanthrene halofantrine is a common antimalarial drug that targets Plasmepsin 2^29^.

*Protein kinase 7 (PDB ID: 2pml)—Terazosin (DB01162)* PfPK7 is an “orphan” kinase with the advantage that it presents different features to those of mammalian homologs^30^. Hence, selectivity may be achieved when targeting PfPK7^30,31^ especially in this absence of a human homolog. In the structure 2pml it is complexed with adenosine 5′-(beta,gamma-Imino)triphosphate, an ATP analog. On the other hand, terazosin is a quinazoline, a selective alpha1-antagonist used for the treatment of symptoms of benign prostatic hyperplasia (BPH). It binds to 2pml interacting with ASN35, ASP123, ASP190, ILE42, LEU101, LEU179, LEU34, LYS55, SER189, TYR117 (Fig. 3B and Supplementary Figure S38b). Interactions are dominated by hydrophobic contacts with only one hydrogen bond observed to ARG32. Terazosin presents similar interactions to known inhibitors that bind to this binding site^30^. However, the two compounds are different in their binding modes. Indeed, terazosin extends toward a more superficial area of the pocket while having a moiety fitting more deeply in the binding site^30^. The ATP analog lies in the pocket, though not in the deepest part, crossing terazosin in an overlay of the two. Also, the two structures are significantly different (Tanimoto similarity 0.23) and terazosin does not show significant similarity with available inhibitors (Target ID: CHEMBL6169) the highest being 0.50 for CHEMBL602580. The two molecules share a common long chain connected to a benzene ring.

*Thioredoxin reductase (PfTrxR, PDB ID: 4j56)—Prazosin (DB00457)* 4j56 is a thioredoxin reductase-thioredoxin structure complexed with its substrate and the prosthetic group flavin adenine dinucleotide (FAD). The enzyme is essential for *Plasmodium falciparum* and is involved in redox homeostasis^32^. It was identified as a putative liver stage target^33^. The compound prazosin is a selective $\alpha$-1-adrenergic receptor antagonist used to treat hypertension. In this case it binds to the FAD binding site in a completely buried pocket. It interacts with some water molecules, ALA191, ALA369, ASP357, CYS88 CYS93, GLY52, LYS96, PRO51, SER212, THR87, VAL233 (Fig. 3C and Supplementary Figure S38c). CYS93, CYS88 form the redox centers for the protein’s function^32^. Prazosin also forms hydrogen bonds with LYS96 and ASP357. The FAD binding pocket is large with a mitigated polar character, in which prazosin occupies a large portion. Its scaffold is characterized by an aromatic quinazoline similar to the quinoxaline found in the *Plasmodium* thioredoxin reductase (CHEMBL4547) inhibitor (CHEMBL380953). The two rings present numerous hydrophobic interactions. Prazosin has a high lipophilic efficiency (9), a molecular weight of 383 and BEI of 29. Further it has been found to be inactive againstagainst a mammalian thioredoxin reductase (PubChem^34^ Bioassay ID 488,772, 488,773, 588,453), a good indication of its selectivity profile.

*Calcium-dependent protein kinase 2 (PfCDPK2, PDB ID: 4mvf)—Abiraterone (DB05812)* 4mvf is the Structure of *Plasmodium falciparum* CDPK2 complexed with an inhibitor, staurosporine. Absent from vertebrates, PfCDPK2 is an interesting target for antimalarial therapy^35^. The compound abiraterone is a steroid and an innovative drug that offers clinical benefit to patients with hormone refractory prostate cancer. Abiraterone binds to 4mvf in a trench-like hydrophobic site and interacts with ALA99, ASP213, CYS149, ILE212, LEU199, LYS101, MET146, VAL130, VAL86 (Fig. 3D and Supplementary Figure S38d). The compound presents a hydrophobic character (low PSA: 33.12 Å^2^ with high logP: 5.39) with interactions dominated by alkyl contacts. A polar contact does present with THR82 on a more exposed area of the binding site. It does not share significant similarity with the enzyme (Target ID CHEMBL1908387) inhibitors, the highest being 0.5 for CHEMBL602580.

In conclusion, identified hits in docking interacted with their respective targets similarly to co-crystallized ligand. This is also supported by their high Grscores (> 0.58). Regarding their structures, most hits presented different scaffolds compared to the respective known inhibitors of the targets. Except for SEI, all ligand efficiencies were in good ranges for drug discovery.

#### Complex stability during MD simulations

All-atom MD simulations for the 25 different protein–ligand systems were performed for 20 ns to provide a profile of their dynamic behavior. MD is an effective means to assess ligand binding mode stability. To investigate both the stability of the apo proteins and complexes, the time evolution of the root mean square deviation (RMSD) and the radius of gyration (Rg) were monitored.

**Figure 4:**
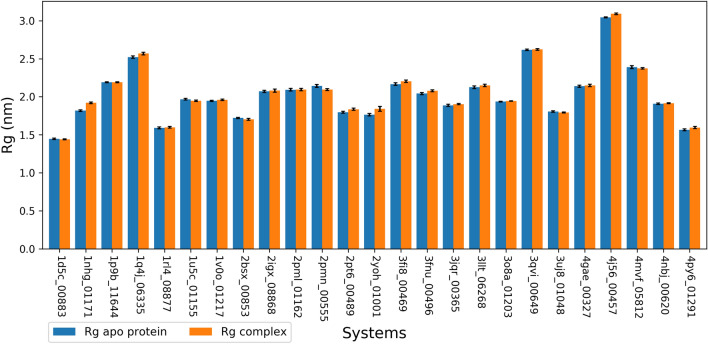
Calculated mean of Rg of the backbone atoms for apo and complexes during the 20 ns simulation. Protein–Ligand systems are represented by their PDB IDs and DrugBank IDs (last five digits). Error bars represent the standard deviation of the means (σ). Color code: orange: complexes; blue: apo proteins.

Rg is a measure related to the overall compactness of the protein which can thus assess structure instability, especially when unfolding^36^. An increasing Rg during simulation indicates that a structure is getting less compact and vice-versa. Figure 4 presents a bar plot of the mean values for the Rg values of the apo and the complexes during the 20 ns simulation.

However, in terms of comparing Rg values between structures, the difference in the Rg values is related to the size of the proteins, large structures having larger Rg values. Rg values for the apo structures ranged from 1.45 nm (1d5c_DB00883) to 3.04 nm (4j56_DB00457) while for the complexes, it ranged from 1.44 nm (1d5c_DB00883) to 3.09 nm (4j56_DB00457). 1d5c and 4j56 thus represent the Rg extreme values in both apo and complex structures.

The small values of the standard deviations indicate the small variation of the Rg values during simulation. Indeed, the highest values of the standard deviation were 0.05 nm for the complexes (2yoh_DB01001) and 0.02 nm for the apo proteins (4mvf_DB05812). Given that these extreme values are still low, we can conclude that both apo and complex structures maintained a constant level of compactness during simulations, indicating their stability.

Further, we compared Rg value for each system in its complex and apo forms. Because small molecules binding to a protein may induce conformation change resulting in a significant change in their Rg^37,38^. The absolute values of the differences (apo – complex) of the mean values of Rg ranged from 0.1 nm (1nhg_DB01171) to 0.04 nm (2pmn_DB00555). A Z-test of the difference of the Rg means (apo—complex) was conducted to assess the differences in the Rg distributions (Supplementary Table S1). All systems showed a p-value < 0.05 except for the complex 4mvf_DB05812, providing evidence that the mean is not equal in the two Rg distributions (apo and complex) for this system. Most systems, 19 out of 25, showed a small increase in their Rg upon ligand binding. However, the highest value in the mean difference is 0.1 nm (1 Å) for the complex 1nhg_DB01171. Hence, ligand binding did not cause significant change in the protein structures. This is also supported by the RMSD analysis.

**Figure 5:**
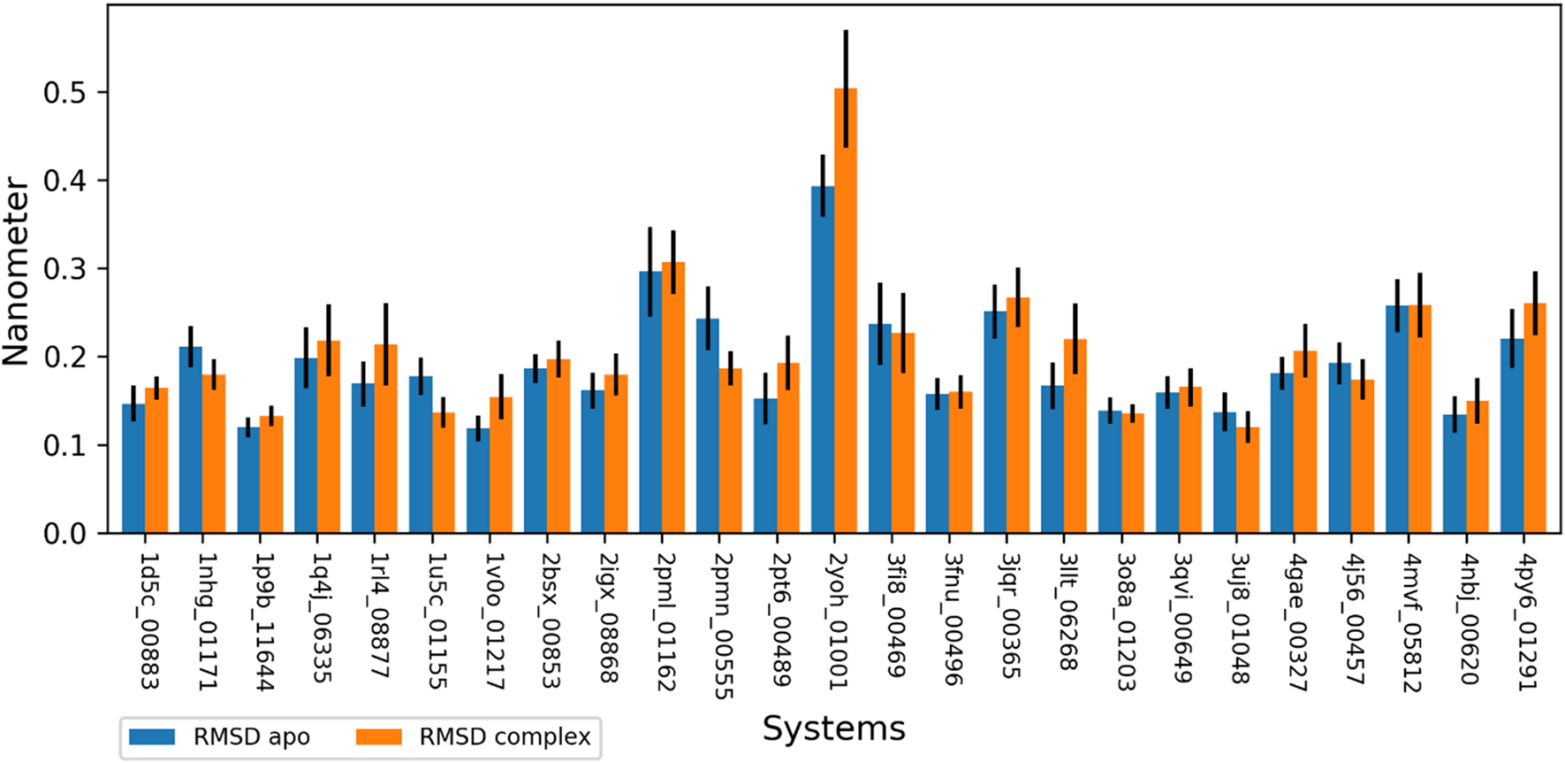
Mean of RMSD of backbone atoms after a least-squares fit to the initial structure for apo proteins and complexes during the 20 ns simulation. Protein–Ligand systems are represented by their PDB IDs and DrugBank IDs (last five digits). Error bars represent the standard deviation of the means (σ). Color code: orange: complexes; blue: apo proteins.

Figure 5 shows the means of RMSD values for apo and complex proteins. RMSD is a similarity metric used to assess structure variation during MD simulation. A value of 3 Ǻ is often put forward as the similarity threshold^39^. RMSD values were in the range of 0.11 nm to 0.47 nm for the apo protein and in the range of 0.20 nm to 0.50 nm for the complexes. The standard deviations ranged from 0.01 nm to 0.15 nm for the apo proteins and from 0.01 nm to 0.06 nm for the complexes. The highest RMSD values were 0.38 nm (2yoh) and 0.5 nm (2yoh_DB01001) for apo proteins and complexes respectively. Hence, the system 2yoh_DB01001 in both apo and complex forms has RMSD values greater than 3 Ǻ. These values are beyond the acceptable threshold for structural similarity despite the low standard deviation values. Indeed, the standard deviations of the RMSD values were 0.035 nm and 0.066 nm for apo and complex forms of 2yoh.

More, we looked at the effects of ligand binding on protein structures variation. In eight out of the 25 systems, the complexes showed decreased structural variation over the apo protein, showing reduced RMSD values for the complex than in the apo case. In the rest, the ligand binding induces structural variation. The highest absolute value of RMSD difference were 0.11 nm for the complex 2yoh_DB01001 followed by 0.05 nm for the complex 3llt_DB06268. These sole differences in protein RMSD may not be significant to affect proteins’ functions.

**Figure 6:**
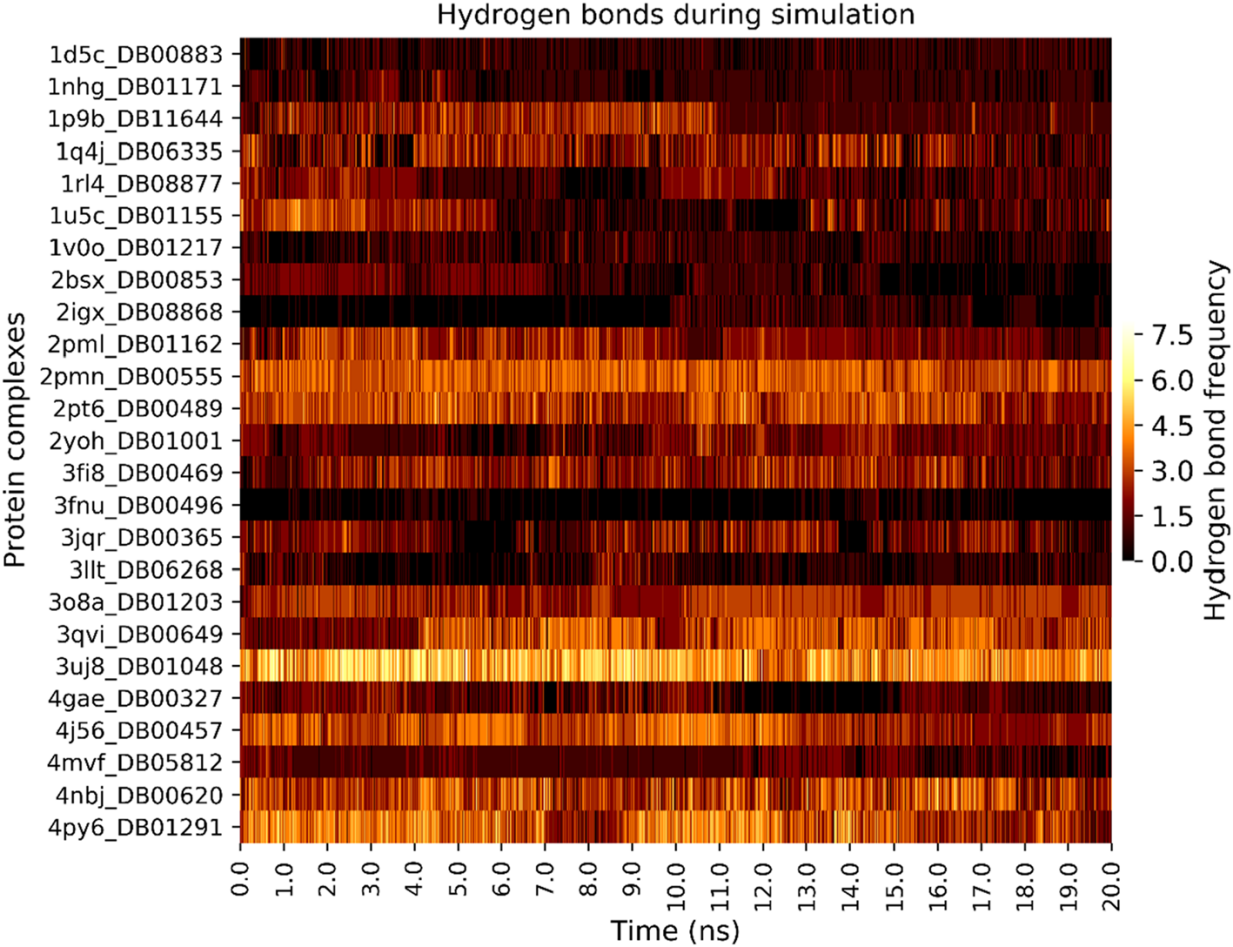
Hydrogen bonds between the protein and the ligand during the 20 ns simulation. Complexes are represented on the y-axis with the PDB ID and DrugBank IDs. The heatmap was generated using Seaborn version 0.9^85^.

To assess ligand stability, the time evolution of the number of hydrogen bond in each complex during simulation was analysed (see Fig. 6). In most systems, ligands maintained the initial number of hydrogen bonds. Darifenacin (DB00496) showed the lowest number of hydrogen bonds (0.22) on average. Its binding to the histo-aspartic protease (PDB ID: 3fnu) is driven by hydrophobic contacts, and as a result, the number of hydrogen bonds is reduced when compared to other systems. Similarly, the complex plasmepsin 2 (2igx)-fingolimod (DB08868) showed an average of 0.39 hydrogen bonds. Fingolimod forms hydrophobic contacts with only one hydrogen bond to ASP121 in the binding site in the docked pose. However, in this system (2igx), it was observed that there was an increase in the number of hydrogen bonds at around 10 ns. Abacavir (DB01048) showed the highest number of hydrogen bonds to 3uj8 compared to any other system. In the docked pose for this compound, a network of five hydrogen bonds was observed. This also correlates with the observed interaction energy for this complex which was the 2nd most favourable of the protein–ligand interaction energies (-261.05 kcal/mol) of all systems (see Supplementary Fig. S3 and Table S2).

Ligand stability was further assessed using ligand RMSD (see Supplementary Fig. S1), by visualization, with the protein–ligand interaction energy (see Supplementary Fig. S3) and the center of mass (COM) distance between the ligand and the protein (see Supplementary Fig. S2). Ligand RMSD fitted to the protein backbone was more sensitive to ligand movements than the ligand RMSD fitted to itself. Indeed, they showed higher values than the ligand RMSD fitted to itself (see Supplementary Fig. S1). However, the high values of ligand RMSD fitted to the protein backbone do not correlate with ligand dissociation as visualized in some systems. Ligand RMSD fitted to the protein captures the movement of the ligand relative to the protein^40^. Thus, the RMSD fitted to the protein backbone and the COM distances were therefore used. COM distances varied from 0.61 nm (2pt6_DB00489) to 2.33 nm (4j56_DB00457) (see Supplementary Fig. S2). Differences in the values are related to the size of the systems. The COM distances are better assessed using their standard deviations, a complex with dissociating ligand will tend to show an increasing COM distance, resulting in a high standard deviation. The highest observed standard deviation was 0.10 nm for the complex 1rl4_DB08877, the lowest value being 0.02 nm for the complex 3o8a_DB01203. Hence, ligands’ stability is also supported by the low COM distances variations. Regarding interaction energy, all systems showed a negative total protein–ligand interaction energy in a range of −140.28 kcal/mol to −331.42 kcal/mol. The system with prazosin (4j56_DB00457) showed the most favourable interaction energy profile with an average value of −331.42 kcal/mol (see Supplementary Table 1 and Fig. S3). The total protein–ligand interaction energy also supports ligand non-dissociation from the protein. The negative energy values indicate favorable interaction with protein residues.

Overall, MD simulations showed stable complexes supported by structures’ RMSD and radius of gyration. Ligand binding did not significantly change the apo proteins. Moreover, hydrogen bonds and interaction energies showed favorable protein–ligand interactions.

The approach here combines binding affinity, molecular interactions, statistical transformations (binding energies standardization, rank transformation and complex ranking) and compound properties through ligand efficiency metrics. Starting with a set of FDA approved compounds, it may be tempting to simply ignore their pharmacological profiles, as approved drugs may imply at least an acceptable drug likeness profile. However, integrating pharmacological properties certainly directs the prioritization of hits and contributes towards fulfilling the multiple objectives-optimization nature of drug discovery. More efficiency indices are highly recommended for evaluation of high-quality hits in medicinal chemistry^23,41–43^. Compounds with low potency may compensate with better molecular and pharmacokinetic properties for improved bioavailability.

Complementary to binding energy, the knowledge-based and topological scoring function GRIM may be used. However, it is noteworthy that it is biased toward the molecular interaction pattern of the co-crystallized ligand. Yet, different inhibitors may present different interaction patterns. This is certainly the case for allosteric inhibitors. However, protein–ligand molecular recognition is often governed by key conserved molecular interactions^44^ as with enzyme substrates. Co-crystallized ligands used as reference ligands in this study to compute the Grscore may still present these conserved interactions.

### Antiplasmodial and human cytotoxicity assays

**Figure 7:**
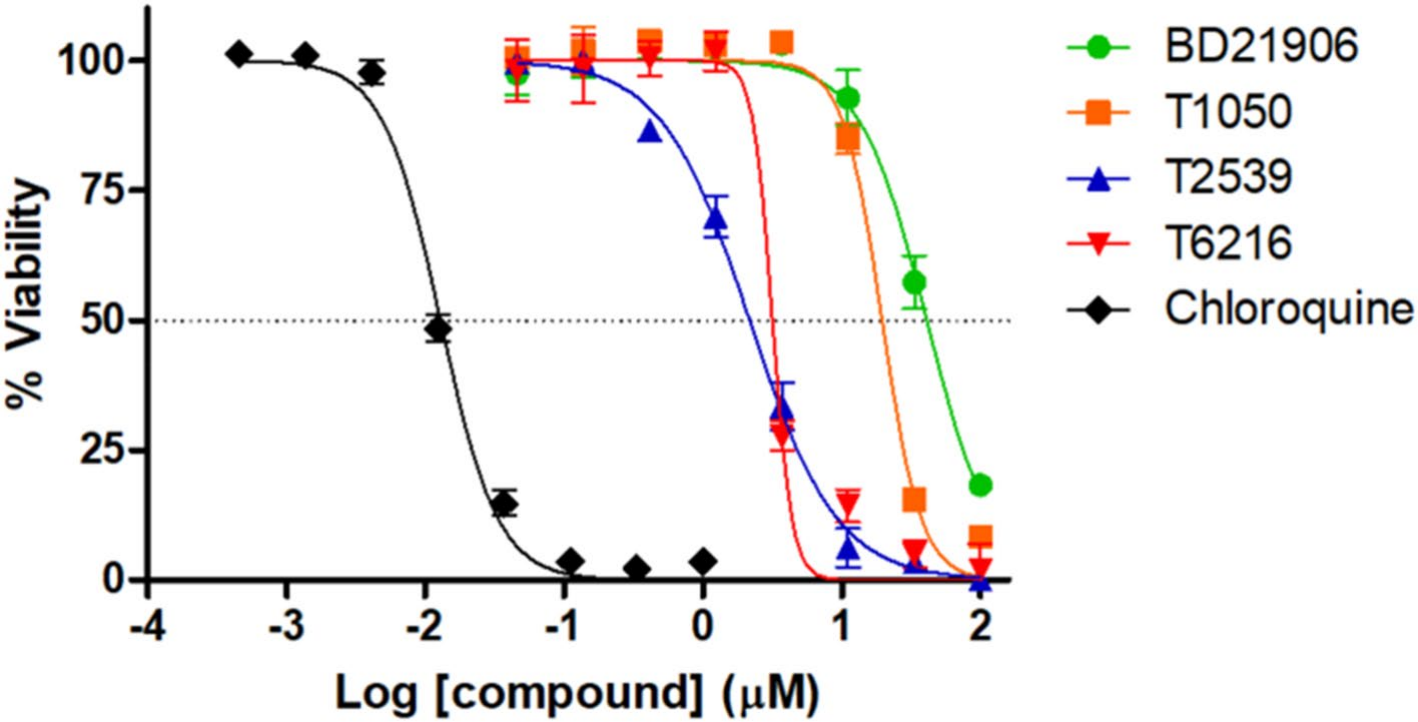
Antiplasmodial dose–response plots. *P. falciparum* viability percentage was plotted against the Log (compound concentration). The IC_50_ (50% inhibitory concentration) was obtained from the curve by non-linear regression. The black curve represents the positive control chloroquine. Compounds were tested in triplicate with the standard deviation (SD) indicated by the error bars. Curves for BD21906, T1050, T2539, T6216 correspond to the following compounds terazosin (DB01162), prazosin (DB00457), fingolimod (DB08868) and abiraterone (DB05812) respectively.

Fingolimod and abiraterone produced IC_50_ values in the single-digit μM range, 2.21 μM and 3.37 μM respectively, against cultured *P. falciparum* (see Fig. 7). Hence these may be promising for further optimization. A total of four compounds showed antiplasmodial activity (see Fig. 7 and Supplementary Figure S37). Prazosin and terazosin showed IC_50_ values of 16.67 μM and 34.72 μM respectively. Active compound targets were Plasmepsin 2, PfPK7, Thioredoxin reductase 2 and Calcium-dependent protein kinase 2 for fingolimod, terazosin, prazosin and abiraterone respectively (see Table 1). Thioredoxin reductase 2 is a putative drug target for liver-stage malaria hits^33^. In a pre-screen at a fixed concentration of 20 μM, salbutamol, lamotrigine and moclobemide decreased cell viability to 71.83%, 72.23%, 61.24% respectively, which was not considered as sufficient to warrant their inclusion as active compounds (see Supplementary Fig. S33). Moclobemide^45,46^ and salbutamol^47^ showed antiplasmodial activity in previous assays^45–47^.

Prazosin and terazosin are two close analogs (Tanimoto similarity 0.7), differing by a furan ring on prazosin while terazosin has a tetrahydrofuran (see Table 1). The two compounds were predicted on two different targets. Terazosin was predicted to bind on PfPK7 while prazosin was on thioredoxin reductase 2 (see Table 1). This may be related to the hit selection process with the complex ranking which selects a hit for each target in the array of protein. Given their structural analogy, both compounds may be binding on the two targets. Hence they may be potential multitarget compounds for these targets. It also possible that one of the targets is the correct one. Despite their structural similarity, prazosin was about two times more active than terazosin. Indeed, prazosin showed an IC50 of 16.67 uM while terazosin had an IC50 of 34.72 uM (see Table 1). A similar difference in the cell viability assay was also observed (see Supplementary Figure S36).

In the human cytotoxicity assays using human cervix adenocarcinoma (HeLa) cells, only fingolimod had a significant cytotoxic effect, reducing HeLa cells viability to below 50% (1.98%) at 20 µM (see Supplementary Figs. S39 and S40). It further showed an IC_50_ of 1.63 µM**.** Fingolimod is an immunosuppressant targeting the sphingosine-1-phosphate receptor on T cell membranes^48^, which may explain its activity on HeLa cells. Interestingly, the compound is also being investigated for COVID-19 treatment^49^.

Regarding the screening pipeline accuracy, the hit rate was 25%. Indeed, 4 hits of the 16 tested hits were active. A 5% hit rate may be considered as successful^50^ with a range between 1 and 40% in prospective screening^51^. It is difficult to measure the contribution of factor to the pipeline GRIM, paired ranking, ligand efficiency metrics. The hit rate is promising given the screening context: protein array, use of only cost-effective methods (ligand efficiency, paired ranking system, rescoring with GRIM, short MD simulation). Future improvements to the workflow may be to use a larger screening library, longer simulation and or binding free energy methods.

## Materials and methods

### Target and ligand preparation

The set of *Plasmodium falciparum* structures in the Screening Protein Data Bank (sc-PDB) (release v.2017 frozen PDB data on 2016–11) was downloaded and only one representative of duplicates (protein having same UniProt ID) from this set was retained. For MD simulation purposes, sc-PDB structures having the least number of missing residues were prioritized. The list of selected structures (PDB ID and co-crystallized ligands) is available in Supplementary Table S3. Sc-PDB structures are designed to suit molecular modelling purposes^52^. Their pdbqt formats were generated using docking using AutoDock Tools^53^. Cofactors were retained in their respective structures. The subset of orally active, Food and Drug Administration (FDA) approved small molecules, compliant with Lipinski’s rule of five, and not affecting and not targeting *Plasmodium spp* was downloaded from DrugBank^21^ (version 5.1.2, released 2018–12-20). This selection was made using DrugBank advanced search menu. For ligand analogs (having a Tanimoto coefficient of similarity greater or equal to 0.8), a single representative was used, chosen according to the best Quantitative estimate of druggability (QED)^54^. Ligands having non-valid AutoDock atom types were removed, leaving a final set containing 796 compounds. These ligands were prepared for docking using AutoDock Tools^53^ and their molecular properties were calculated using RDKit (version 2018.09.1)^55^. cLogP values were calculated using Crippen’s approach available in the RDKit package^55,56^.

### Docking parameters

An alternate version of Autodock Vina^57^, QuickVina-W^19^ was used in a blind docking experiment. QuickVina-W has been reported to achieve an average acceleration factor of 3.60 compared to Vina, without losing accuracy in terms of pose and affinity predictions. The scoring function is identical to that of AutoDock Vina^58^ but the search algorithms are more efficient making QuickVina-W ideal for blind docking^19^. Exhaustiveness was scaled to each target box size using a reference value of 24 for a box size of 303 angstroms (24 is three times the default Autodock Vina exhaustiveness) and ten poses were predicted in each docking. A parallelization scheme with 3 CPU (central processing unit) per docking calculation (internal parallelization) and 8 jobs per computer node (each node having 24 cores) (external parallelization) was used for optimal screening performance as reported in the literature^59^. Co-crystallized ligands were used to validate the docking process. An RMSD value between docked and co-crystallized ligands up to 2.00 Å was considered as good poses as is often reported in posing assessments^11,60^. RMSD values were computed using GROMACS^61^ version 2016 without least-squares fitting of the structures.

### Scoring functions

QuickVina-W and Graph Interaction Matching (GRIM) (belonging to two classes of scoring functions: topological and energetical scoring functions) were used. GRIM was used to re-score the poses generated by QuickVina-W. GRIM is a topological, knowledge-based scoring function that scores interactions pattern similarity between the docked ligand and the co-crystallized one (reference ligand used for comparison). The tool provides a Grscore, ranging from 0.0 to 1.5 and a pose with a Grscore greater than 0.59 has a significant similarity of interaction pattern with the reference ligand^26^. From the D3R Grand Challenge 2, on a dataset of 102 FXR agonists, posing with HYDE^62^ and scoring with GRIM-1 gave a Kendall’s τ ranking coefficient of 0.442, the third most accurate ranking^63^.

### Standardization

Binding affinities may differ significantly across different proteins due to peculiarities of their active sites (volume, depth, hydrophobic character etc.). The so-called interprotein scoring noise has been illustrated in different studies and score normalization strategies have been proposed to eliminate the scoring bias in reverse docking^64–67^. A similar phenomenon has been observed also for ligands which tend to show higher affinity with the increase of molecular weight, leading to false positives in docking. Normalizing this bias has been shown to improve ligand affinity ranking in virtual screening^20, 66, 68^. Here we used the z-score for standardization, subtracting the mean and dividing by the standard deviation (Eq. 1). For the resulting scores from screening, this was applied to each column (series of all ligand docking scores on each particular protein) and then on each row (series of a single ligand’s score across the set of proteins) to obtain the z-score as implemented in the python package SciPy^68^.

1 $$z{ - }score = \frac{x - \mu }{\sigma }$$

The standardization scheme was independently applied to the set of scores on each protein, centring the docking scores around a mean of 0 with a standard deviation of 1. This reduces the noise caused by the differences in binding pockets and scores for different ligands on different proteins can thus be compared.

### Ligand efficiency metrics

The ligand efficiency indices Binding efficiency index^42^ (BEI) (Eq. 2), Surface efficiency index^42^ (SEI) (Eq. 3) and Lipophilic efficiency^41^ (LipE) (Eq. 4) metrics were used. LipE, SEI and BEI are three main ligand efficiency metrics which have recently gained much interest in identifying quality hits and further optimization in medicinal chemistry^42^. Navigating the 2D efficiency plane has been shown to be an effective guide for medicinal chemistry. Selected hits based on high scoring ligand efficiency metrics can lead to high-quality leads^23,41–43^.

2 $$BEI = \frac{pIC50}{{MW\,\left( {{\text{kDa}}} \right)}}$$

3 $$SEI = \frac{pIC50}{{\left( {PSA/100\,\AA ^{2} } \right)}}$$

4 $$LipE = pIC50 - logP$$

IC50, Kd, and Ki are interchangeable. MW: Molecular Weight. PSA: Polar surface area. The binding energies were converted to Ki (dissociation constant of the enzyme-inhibitor complex) using Eq. (5).

5 $$K_{i} \left( {\text{unit in Molar}} \right) = \frac{{e^{{\frac{1,000 \times \Delta G}{{RT}}}} }}{1,000}$$

In Eq. (5), $\Delta$ G is the binding affinity (kcal/mol), $R = 1.98$, $T = 298.15\,Kelvin$ and the final ${\text{ pKi}}$ is given in Eq. (6).

6 $${\text{pKi}} = {\text{log}}10\left( {{\text{Ki}}_{{{\text{Molar}}}} } \right)$$

It is noteworthy that of two distribution coefficients available, LogD is more accurate for charged compounds than the calculated partition coefficients (log P)^69^. However, LogP was used for simplicity since logD requires determining pKa (dissociation constant), and this calculation was not available in RDKit.

A combined metric of SEI and BEI which can be derived from the 2D efficiency plane^42^ is simply the radial coordinate which corresponds to $\sqrt {SEI^{2} + BEI^{2} }$.

Binding energy is implicitly included in LipE, SEI and BEI through the $pIC_{50}$.

### Rank transformation and complex ranking

LipE, SEI, BEI and the Grscore were combined using the rank transformation after score standardization, transforming each standardized score to its rank. For equal scores, a rank that is the average of the ranks of those scores is taken. Complexes (protein–ligand) were ranked using the complex ranking scheme^50^. A complex rank is defined as the sum of the protein rank and ligand rank and calculated using Eq. (7).

7 $${\text{Complex}}_{{{\text{rank}}}} = {\text{ligand}}_{{{\text{rank}}}} + {\text{protein}}_{{{\text{rank}}}}$$

The ${\text{ligand}}_{{{\text{rank}}}}$ relative to a protein, is its rank compared to all other ligands. In a similar way, the ${\text{protein}}_{{{\text{rank}}}}$ relative to the ligand is the rank of the protein compared to all other proteins. Thus, a complex rank is simply the sum of the ligand and protein ranks. This allows revealing protein–ligand having high specificity for each other (top-ranked complexes) contributing to filter out false positives. Top-ranked complexes will have low rank values (the lowest rank value being 2 and the highest 832)^50^.

### Molecular Dynamics

Molecular dynamics (MD) simulation on protein–ligand complexes was conducted as the final screening step to assess our complexes stability and to filter out some false positives. Proteins having missing residues in structures were first modelled using Prime version 5.4 (r012) (Schrodinger2018-4)^70^. Metal ions (MG in 1d5c, MG 1p9b, MN in 2pml and MG in 3fi8) were not included in the simulations. All systems were simulated during 20 ns in a dodecahedron box with a distance between the solute and the box set to 1.0 nm. The tip3p water model was used with a concentration of 0.15 M (Na + (sodium) and Cl- (chloride) ions). Systems’ energies were minimized using the steepest descent method with a maximum force set at < 1,000.0 kJ/mol/nm with a maximum number of steps of 50,000 followed by equilibration at 300 K and 1 atm with 50 ps of MD simulation in the isothermal-isobaric ensemble and subsequently in the canonical one. For the Lennard–Jones and the short-range electrostatic interactions, a cutoff of 10 Å was used. For the long-range electrostatic interactions, the smooth particle mesh Ewald method and a fourth-order interpolation scheme were used. Simulations were done using the leap-frog algorithm for integration. Hydrogen mass repartitioning (HMR) was applied to the proteins and ligands: masses of hydrogens bound to heavy atoms were repartitioned allowing an accelerated 4-fs time step. HMR has been shown to accelerate MD simulations without loss of accuracy^71–74^. Ligand topologies were generated using ACPYPE^75^ with their charges obtained from Discovery Studio Visualizer V1.7, also used to analyse protein–ligand interactions. HMR was also applied to the ligand: increasing the hydrogen-mass by a factor of four and subtracting the added mass from the bonded heavy atom as described in GROMACS^76^ documentation, thus, conserving the total mass of the system. Simulations were conducted on a remote machine at CHPC (Center for High-Performance Computing) with GROMACS^76^ version 2018.2 using the Amber ff99SB-ILDN^77^ force field. After simulation, the GROMACS module trjconv was used to correct for periodicity. Analysis consisted of first assessing the stability of the proteins’ structures through their root mean square deviation (RMSD) and the radius of gyration (Rg) calculated using the GROMACS^76^ package. The stability of the ligand binding pose was measured using the RMSD of the ligand heavy atoms after least-squares fitted to the protein Backbone and also by the interactions between the ligands and the protein through the number of hydrogen bonds and the total protein–ligand interaction energy. Protein rotation and translation were first removed by fitting it to the starting structure. Trajectories were visualized in the Jupyter Notebook^78^ using Nglview^79^ and the Pytraj package^80^.

### Antiplasmodial assay

Antiplasmodial assessment of the compounds against the 3D7 strain of *Plasmodium falciparum* was carried out as described previously^81^. Briefly, compounds were incubated with cultured parasites at a final concentration of 20 μM for 48 h and percentage parasite viability relative to untreated control parasites determined using the plasmodium lactate dehydrogenase (pLDH) assay^82^. Compounds that decreased parasite viability > 50% in this initial screen were subjected to dose–response assays to determine their IC_50_ values. The 48-h incubation followed by the pLDH assay was repeated with threefold serial dilutions of the test compounds and IC_50_ values determined by non-linear regression analysis of % parasite viability vs. log[compound] plots.

### Human cytotoxicity assay

To assess the overt cytotoxicity of the test compounds, they were incubated at a single concentration of 20 µM or three-fold serial dilutions (100 to 0.0457 μM) in 96-well plates containing HeLa cells (human cervix adenocarcinoma, maintained in a culture medium made of Dulbecco’s Modified Eagle’s Medium (DMEM) with 5 mM L-glutamine (Lonza), supplemented with 10% fetal bovine serum (FBS) and antibiotics (penicillin/streptomycin/ amphotericin B) at 37 °C in a 5% CO_2_ incubator for 24 h. The numbers of cells surviving drug exposure were counted using the resazurin based reagent and resorufin fluorescence quantified (Excitation560/Emission590) in a SpectraMax M3 plate reader (Molecular Devices). Fluorescence readings obtained for the individual wells were converted to % cell viability relative to the average readings obtained from untreated control wells (HeLa cells without test compounds), after subtracting background readings obtained from wells without cells. Plots of % cell viability vs. Log(compound concentration) were used to determine IC_50_ values by non-linear regression using GraphPad Prism (v. 5.02)^83,84^.

## Conclusions

For malaria elimination, an accelerated drug discovery pipeline within the context of drug resistance is required. In this context, we propose an in-silico strategy using drug repurposing through the screening of a set of DrugBank compounds against a set of *P. falciparum* structures. This workflow combines ligand efficiency indices and molecular interaction similarity and statistical transformations. Hits showed good ligand efficiency indices and have shown stability in MD simulations. Fingolimod, abiraterone, prazosin and terazosin showed IC_50_ values against cultured *P. falciparum* of 2.21 μM, 3.37 μM, 16.67 μM and 34.72 μM respectively. Further investigation of these hits could lead to new antimalarials for prophylaxis, transmission-blocking, efficient cure and disease eradication.

## Supplementary Information

Supplementary Information 1.

## Abbreviations

ACT: Artemisinin-based combination therapy
ADP: Adenosine diphosphate
AMP: Adenosine monophosphate
ATP: Adenosine triphosphate
BEI: Binding Efficiency Index
CHPC: Center for High-Performance Computing
COM: Center of mass
COPD: Chronic obstructive pulmonary disorder
DHNCA: 3,7-dihydroxynaphthalene-2-carboxylic acid
FAD: Flavin adenine dinucleotide
FDA: Food and Drug Administration
FXR: Farnesoid X receptor
GRIM: GRaph Interaction Matching
GROMACS: GROningen MAchine for Chemical Simulations
HMR: Hydrogen mass repartitioning
HYDE: HYdrogen bond and DEhydration
IMO: Inosine monophosphate
IMP: Inosine monophosphate
MAOI: Monoamine oxidase inhibitor
MD: Molecular dynamics
MG: Magnesium
MMPBSA: Molecular mechanics Poisson–Boltzmann surface area
MW: Molecular weight
NAD: Nicotinamide adenine dinucleotide oxidized
NADH: Nicotinamide adenine dinucleotide reduced
PSA: Polar surface area
QED: Quantitative estimate of druggability
RMSD: Root mean square deviation
RMSF: Root mean square fluctuation
RNA: Ribonucleic acid
SAR: Structure–activity relationship
SEI: Surface Efficiency Index
WHO: World Health Organization

## References

[1] World Health Organization. *WHO Malaria report 2019*. *Malaria report 2019* https://www.who.int/publications-detail/world-malaria-report-2019 (2019).

[2] Lunev, S., Batista, F. A., Bosch, S. S., Wrenger, C. & Groves, M. R. *Identification and Validation of Novel Drug Targets for the Treatment of Plasmodium falciparum Malaria: New Insights*. *Current Topics in Malaria* (InTech, 2016). 10.5772/65659.

[3] Hemingway J (2016). Tools and strategies for malaria control and elimination: what do we need to achieve a grand convergence in Malaria?. PLoS Biol..

[4] Ma D-L, Chan DS-H, Leung C-H (2013). Drug repositioning by structure-based virtual screening. Chem. Soc. Rev..

[5] Vora P, Somani R, Jain M (2016). Drug repositioning: an approach for drug discovery. Mini. Rev. Org. Chem..

[6] Barratt MJ, Frail D (2012). Drug Repositioning: Bringing New Life to Shelved Assets and Existing Drugs.

[7] Verlinden BK, Louw A, Birkholtz L-M (2016). Resisting resistance: is there a solution for malaria?. Expert Opin. Drug Discov..

[8] Ashburn TT, Thor KB (2004). Drug repositioning: identifying and developing new uses for existing drugs. Nat. Rev. Drug Discov..

[9] Kumar A, Naguib YW, Shi YC, Cui Z (2016). A method to improve the efficacy of topical eflornithine hydrochloride cream. Drug Deliv..

[10] Li YY, Jones SJM (2012). Drug repositioning for personalized medicine. Genome Med..

[11] Gathiaka S (2016). D3R grand challenge 2015: Evaluation of protein-ligand pose and affinity predictions. J. Comput. Aided. Mol. Des..

[12] Li Y, Han L, Liu Z, Wang R (2014). Comparative assessment of scoring functions on an updated benchmark: 2. Evaluation methods and general results. J. Chem. Inf. Model..

[13] Williams CJ (2018). MolProbity: More and better reference data for improved all-atom structure validation. Protein Sci..

[14] Robien MA (2005). Crystal structure of glyceraldehyde-3-phosphate dehydrogenase from *Plasmodium falciparum* at 2.25 Å resolution reveals intriguing extra electron density in the active site. Proteins Struct. Funct. Bioinf..

[15] Nasamu AS, Polino AJ, Istvan ES, Goldberg DE (2020). Malaria parasite plasmepsins: More than just plain old degradative pepsins. J. Biol. Chem..

[16] Ash J, Fourches D (2017). Characterizing the chemical space of ERK2 kinase inhibitors using descriptors computed from molecular dynamics trajectories. J. Chem. Inf. Model..

[17] Oliver JC, Linger RS, Chittur SV, Davisson VJ (2013). Substrate activation and conformational dynamics of guanosine 5′-monophosphate synthetase. Biochemistry.

[18] Ballut L (2015). Active site coupling in *Plasmodium falciparum* GMP synthetase is triggered by domain rotation. Nat. Commun..

[19] Hassan NM, Alhossary AA, Mu Y, Kwoh CK (2017). Protein-ligand blind docking using QuickVina-W with inter-process spatio-temporal integration. Sci. Rep..

[20] Shityakov S, Förster C (2014). In silico predictive model to determine vector-mediated transport properties for the blood-brain barrier choline transporter. Adv. Appl. Bioinf. Chem..

[21] Lo EJ (2017). DrugBank 5.0: a major update to the DrugBank database for 2018. Nucleic Acids Res..

[22] Trott O, Olson AJ (2010). AutoDock Vina: improving the speed and accuracy of docking with a new scoring function, efficient optimization, and multithreading. J. Comput. Chem..

[23] Mignani S (2018). Present drug-likeness filters in medicinal chemistry during the hit and lead optimization process: how far can they be simplified?. Drug Discov. Today.

[24] Kumar M, Kaur T, Sharma A (2017). Role of computational efficiency indices and pose clustering in effective decision making: an example of annulated furanones in Pf-DHFR space. Comput. Biol. Chem..

[25] Mendez D (2019). ChEMBL: towards direct deposition of bioassay data. Nucleic Acids Res..

[26] Desaphy J, Raimbaud E, Ducrot P, Rognan D (2013). Encoding protein-ligand interaction patterns in fingerprints and graphs. J. Chem. Inf. Model..

[27] Boss C (2006). Achiral, cheap, and potent inhibitors of plasmepsins I, II, and IV. ChemMedChem.

[28] Barratt E (2006). Thermodynamic penalty arising from burial of a ligand polar group within a hydrophobic pocket of a protein receptor. J. Mol. Biol..

[29] Friedman R, Caflisch A (2009). Discovery of plasmepsin inhibitors by fragment-based docking and consensus scoring. ChemMedChem.

[30] Merckx A (2008). Structures of *P. falciparum* protein kinase 7 identify an activation motif and leads for inhibitor design. Structure.

[31] Cabrera DG (2018). Plasmodial kinase inhibitors: license to cure?. J. Med. Chem..

[32] Fritz-Wolf K (2013). Crystal structure of the *Plasmodium falciparum* thioredoxin reductase-thioredoxin complex. J. Mol. Biol..

[33] Derbyshire ER, Prudêncio M, Mota MM, Clardy J (2012). Liver-stage malaria parasites vulnerable to diverse chemical scaffolds. Proc. Natl. Acad. Sci. USA.

[34] Kim S (2019). PubChem 2019 update: improved access to chemical data. Nucleic Acids Res..

[35] Färber PM, Graeser R, Franklin RM, Kappes B (1997). Molecular cloning and characterization of a second calcium-dependent protein kinase of *Plasmodium falciparum*. Mol. Biochem. Parasitol..

[36] Lobanov MY, Bogatyreva NS, Galzitskaya OV (2008). Radius of gyration as an indicator of protein structure compactness. Mol. Biol..

[37] Seeliger D, De Groot BL (2010). Conformational transitions upon ligand binding: Holo-structure prediction from apo conformations. PLoS Comput. Biol..

[38] Donev, R. *Personalized Medicine, Volume 102 - 1st Edition*.

[39] Maiorov, V. N. & Crippen, G. M. *Size‐independent comparison of protein three‐dimensional structures*. *Proteins: Structure, Function, and Bioinformatics* vol. 22. 10.1002/prot.340220308 (1995). 10.1002/prot.340220308 7479700

[40] Lemkul J (2019). From proteins to perturbed hamiltonians: a suite of tutorials for the GROMACS-2018 molecular simulation package [Article v1.0]. Living J. Comput. Mol. Sci..

[41] Leeson PD, Springthorpe B (2007). The influence of drug-like concepts on decision-making in medicinal chemistry. Nat. Rev. Drug Discov..

[42] Abad-Zapatero C (2007). Ligand efficiency indices for effective drug discovery. Expert Opin. Drug Discov..

[43] Freeman-Cook KD, Hoffman RL, Johnson TW (2013). Lipophilic efficiency: the most important efficiency metric in medicinal chemistry. Future Med. Chem..

[44] Slynko I, Da Silva F, Bret G, Rognan D (2016). Docking pose selection by interaction pattern graph similarity: application to the D3R grand challenge 2015. J. Comput. Aided. Mol. Des..

[45] Trager W (1976). Human malaria parasites in continuous culture. Science (80-).

[46] Plouffe D (2008). In silico activity profiling reveals the mechanism of action of antimalarials discovered in a high-throughput screen. Proc. Natl. Acad. Sci. USA.

[47] Prado-Prado FJ, García-Mera X, González-Díaz H (2010). Multi-target spectral moment QSAR versus ANN for antiparasitic drugs against different parasite species. Bioorg. Med. Chem..

[48] Brody, T. Clinical Trials 2nd edition. https://www.elsevier.com/books/clinical-trials/brody/978-0-12-804217-5 (2016).

[49] Fingolimod in COVID-19 - Full Text View - ClinicalTrials.gov. https://clinicaltrials.gov/ct2/show/NCT04280588.

[50] Li YY, An J, Jones SJM (2011). A computational approach to finding novel targets for existing drugs. PLoS Comput. Biol..

[51] Zhu T (2013). Hit identification and optimization in virtual screening: practical recommendations based upon a critical literature analysis. J. Med. Chem..

[52] Desaphy J, Bret G, Rognan D, Kellenberger E (2015). Sc-PDB: A 3D-database of ligandable binding sites-10 years on. Nucleic Acids Res..

[53] Huey, R. & Morris, G. M. *Using AutoDock with AutoDockTools: A Tutorial*. http://mgltools.scripps.edu/downloads/previous-releases/downloads/tars/releases/DocTars/DOCPACKS/AutoDockTools/doc/UsingAutoDockWithADT.pdf.

[54] Bickerton GR, Paolini GV, Besnard J, Muresan S, Hopkins AL (2012). Quantifying the chemical beauty of drugs. Nat. Chem..

[55] Landrum, G. RDKit: open-source cheminformatics software. (2016).

[56] Wildman SA, Crippen GM (1999). Prediction of physicochemical parameters by atomic contributions. J. Chem. Inf. Comput. Sci..

[57] Trott, O. & Olson, A. J. AutoDock Vina: Improving the speed and accuracy of docking with a new scoring function, efficient optimization, and multithreading. *J. Comput. Chem.* **31**, NA-NA (2009). 10.1002/jcc.21334 PMC3041641 19499576

[58] Li H, Leung K-S, Wong M-H, Ballester PJ (2015). Improving AutoDock vina using random forest: the growing accuracy of binding affinity prediction by the effective exploitation of larger data sets. Mol. Inform..

[59] Jaghoori MM, Bleijlevens B, Olabarriaga SD (2016). 1001 Ways to run AutoDock Vina for virtual screening. J. Comput. Aided. Mol. Des..

[60] Affonso RS, Guimarães AP, Oliveira AA, Slana GBC, França TCC (2013). Applications of molecular modeling in the design of new insect repellents targeting the odorant binding protein of anopheles gambiae. J. Braz. Chem. Soc..

[61] Abraham MJ (2015). GROMACS: High performance molecular simulations through multi-level parallelism from laptops to supercomputers. SoftwareX.

[62] Schneider N, Lange G, Hindle S, Klein R, Rarey M (2013). A consistent description of HYdrogen bond and DEhydration energies in protein–ligand complexes: methods behind the HYDE scoring function. J. Comput. Aided. Mol. Des..

[63] Da Silva Figueiredo Celestino Gomes P, Da Silva F, Bret G, Rognan D (2018). Ranking docking poses by graph matching of protein–ligand interactions: lessons learned from the D3R Grand Challenge 2. J. Comput. Aided. Mol. Des..

[64] Luo Q (2017). The scoring bias in reverse docking and the score normalization strategy to improve success rate of target fishing. PLoS ONE.

[65] Vigers GPA, Rizzi JP (2004). Multiple active site corrections for docking and virtual screening. J. Med. Chem..

[66] Jacobsson M, Karlén A (2006). Ligand bias of scoring functions in structure-based virtual screening. J. Chem. Inf. Model..

[67] Fukunishi Y, Kubota S, Nakamura H (2006). Noise reduction method for molecular interaction energy: application to in silico drug screening and in silico target protein screening. J. Chem. Inf. Model..

[68] Virtanen P (2020). SciPy 1.0: fundamental algorithms for scientific computing in Python. Nat. Methods.

[69] Arnott, J. A., Kumar, R. & Planey, S. L. Lipophilicity indices for drug development. *J. Appl. Biopharm. Pharmacokinet.* (2013).

[70] Jacobson MP (2004). A hierarchical approach to all-atom protein loop prediction. Proteins Struct. Funct. Genet..

[71] Pines G (2018). Genomic deoxyxylulose phosphate reductoisomerase (DXR) mutations conferring resistance to the antimalarial drug fosmidomycin in *E. coli*. ACS Synth. Biol..

[72] Hopkins CW, Le Grand S, Walker RC, Roitberg AE (2015). Long-time-step molecular dynamics through hydrogen mass repartitioning. J. Chem. Theory Comput..

[73] Maláč K, Barvík I (2013). Complex between Human RNase HI and the phosphonate-DNA/RNA duplex: Molecular dynamics study. J. Mol. Graph. Model..

[74] Gu S (2017). Phosphoantigen-induced conformational change of butyrophilin 3A1 (BTN3A1) and its implication on Vγ9Vδ2 T cell activation. Proc. Natl. Acad. Sci. USA.

[75] Sousa da Silva AW, Vranken WF (2012). ACPYPE—AnteChamber PYthon Parser interfacE. BMC Res. Notes.

[76] Pronk S (2013). GROMACS 4.5: a high-throughput and highly parallel open source molecular simulation toolkit. Bioinformatics.

[77] Lindorff-Larsen K (2010). Improved side-chain torsion potentials for the Amber ff99SB protein force field. Proteins.

[78] Kluyver, T. *et al.* Jupyter Notebooks-a publishing format for reproducible computational workflows. in *ELPUB* 87–90 (2016).

[79] Nguyen H, Case DA, Rose AS (2018). NGLview–interactive molecular graphics for Jupyter notebooks. Bioinformatics.

[80] Nguyen H, Roe DR, Swails J, Case DA (2016). PYTRAJ: Interactive Data Analysis for Molecular Dynamics Simulations.

[81] Lunga MJ (2018). Expanding the SAR of nontoxic antiplasmodial indolyl-3-ethanone ethers and thioethers. ChemMedChem.

[82] Makler MT, Hinrichs DJ (1993). Measurement of the lactate dehydrogenase activity of *Plasmodium falciparum* as an assessment of parasitemia. Am. J. Trop. Med. Hyg..

[83] Borra RC, Lotufo MA, Gagioti SM, Barros FM, Andrade PM (2009). A simple method to measure cell viability in proliferation and cytotoxicity assays. Braz. Oral Res..

[84] Riss, T. L. *et al.* Cell viability assays. in *Assay Guidance Manual [Internet]* (Eli Lilly & Company and the National Center for Advancing Translational Sciences, 2016). 22553861

[85] seaborn: statistical data visualization — seaborn 0.11.0 documentation. https://seaborn.pydata.org/.

[86] Wishart DS (2005). DrugBank: a comprehensive resource for in silico drug discovery and exploration. Nucleic Acids Res..

[87] O’Boyle NM (2011). Open Babel: an open chemical toolbox. J. Cheminform..

