# Supplementary material for: Potential repurposing of four FDA approved compounds with antiplasmodial activity identified through proteome scale computational drug discovery and in vitro assay
Source: Sci Rep. 2021 Jan 14;11:1413. doi: 10.1038/s41598-020-80722-2 (PMC7809352; doi:10.1038/s41598-020-80722-2)
Supplement: Supplementary file 1 — Supplementary Information 1. [file 41598_2020_80722_MOESM1_ESM.docx]

Potential repurposing of four FDA approved compounds with antiplasmodial activity identified through proteome scale computational drug discovery and *in vitro* assay

Bakary N’tji Diallo^1^, Tarryn Swart^3^, Heinrich C. Hoppe^3^, Özlem Tastan Bishop^1^, and Kevin Lobb^1,2^,*

1 Research Unit in Bioinformatics (RUBi), Department of Biochemistry and Microbiology. Rhodes University, Grahamstown, 6140, South Africa.

2 Department of Chemistry, Rhodes University, Grahamstown, 6140, South Africa.

3 Department of Biochemistry & Microbiology, Rhodes University, Grahamstown 6140, South Africa

* Correspondence: K.Lobb@ru.ac.za; Tel.: +27 (0) 46 603 8683 (K.L.)

Contents

[1. Supplementary Data 3](#_Toc55300173)

[1.1 Complex hits ligand RMSDs during simulation 3](#_Toc55300174)

[1.2 Complex hits protein Ligand COM distance during simulation 3](#_Toc55300175)

[1.3 Complex hits protein Ligand interaction energy during simulation 4](#_Toc55300176)

[1.4 Top predicted complexes binding poses 5](#_Toc55300177)

[1.5 Z-test mean apo Rg vs mean complex Rg 40](#_Toc55300178)

[1.6 Scoring schemes assessment through the Mean Ranking Error (MRE) 42](#_Toc55300179)

[1.7 Top predicted complexes 47](#_Toc55300180)

[1.8 Targets information 48](#_Toc55300181)

[1.9 In vitro assays 50](#_Toc55300182)

[2. References 56](#_Toc55300183)

1. Supplementary Data

## Complex hits ligand RMSDs during simulation


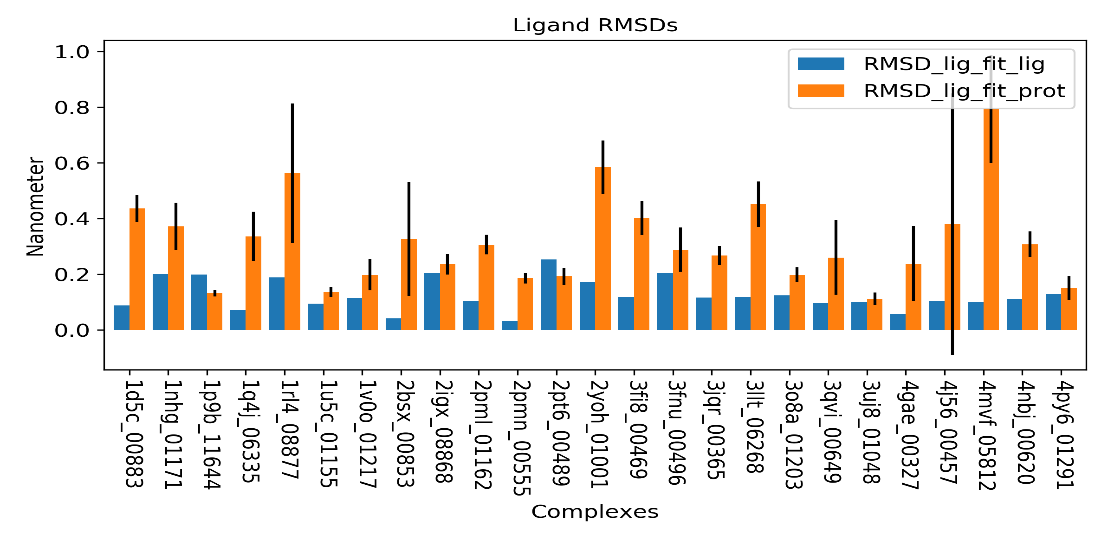


Figure S1 Ligand RMSDs. Blue (RMSD_lig_fit_lig): of the ligand fitted to itself. Orange RMSD (RMSD_lig_fit_prot): RSMD of ligand fitted to the protein backbone.

## Complex hits protein Ligand COM distance during simulation


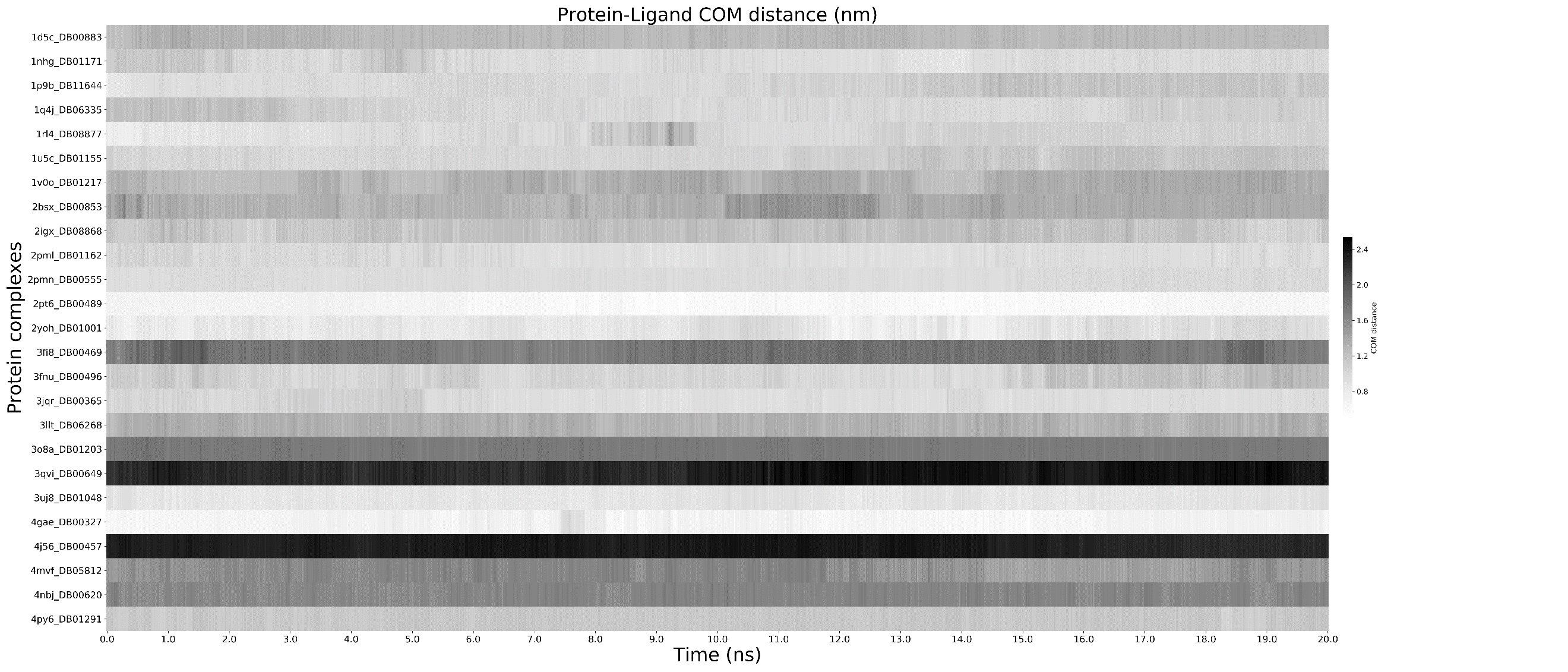


Figure S2 Top Complexes Protein Ligand COM distance. The heatmap was generated using Seaborn version 0.9^1^

## Complex hits protein Ligand interaction energy during simulation


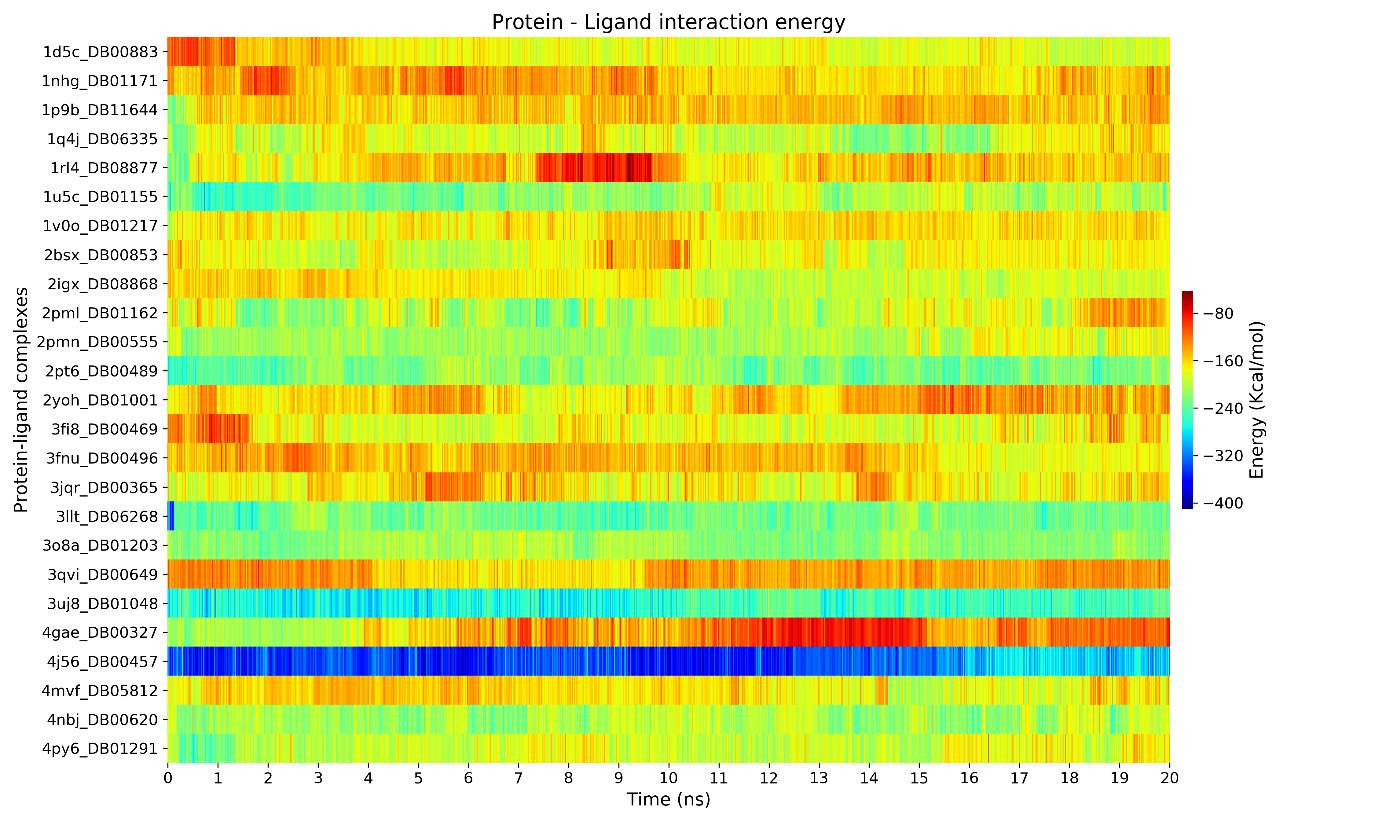


Figure S3 Top Complexes interaction energy. The heatmap was generated using Seaborn version 0.9^1^.

## Top predicted complexes binding poses


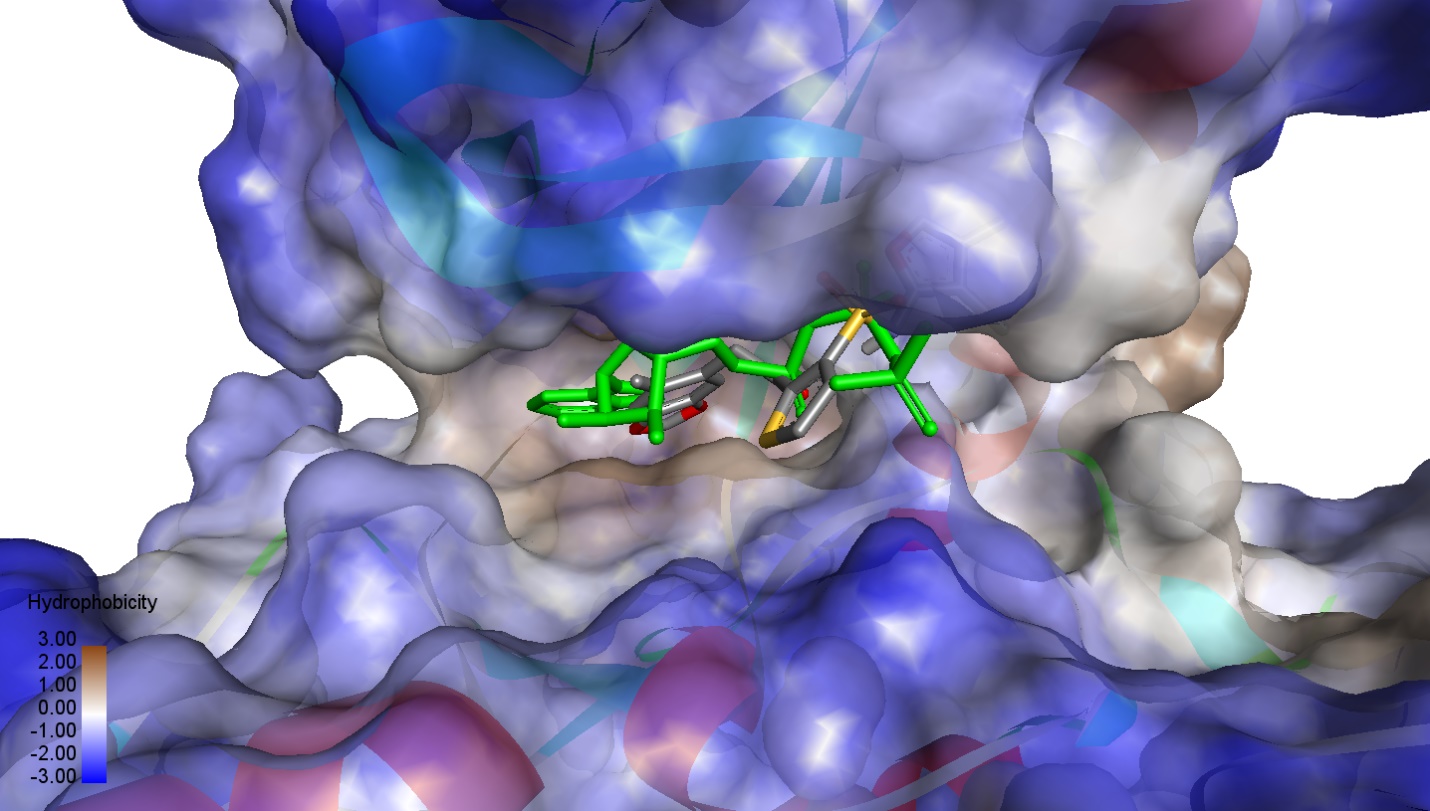


Figure S4 Binding modes of sitaxentan (atom type) and ANP (green) in protein serine/threonine kinase-1 active site. A surface with the hydrophobicity color scheme is created around the protein. Color scheme code: brown = hydrophobic, blue = hydrophilic and white. The image is rendered using Discovery Studio Visualizer 2017 R2.


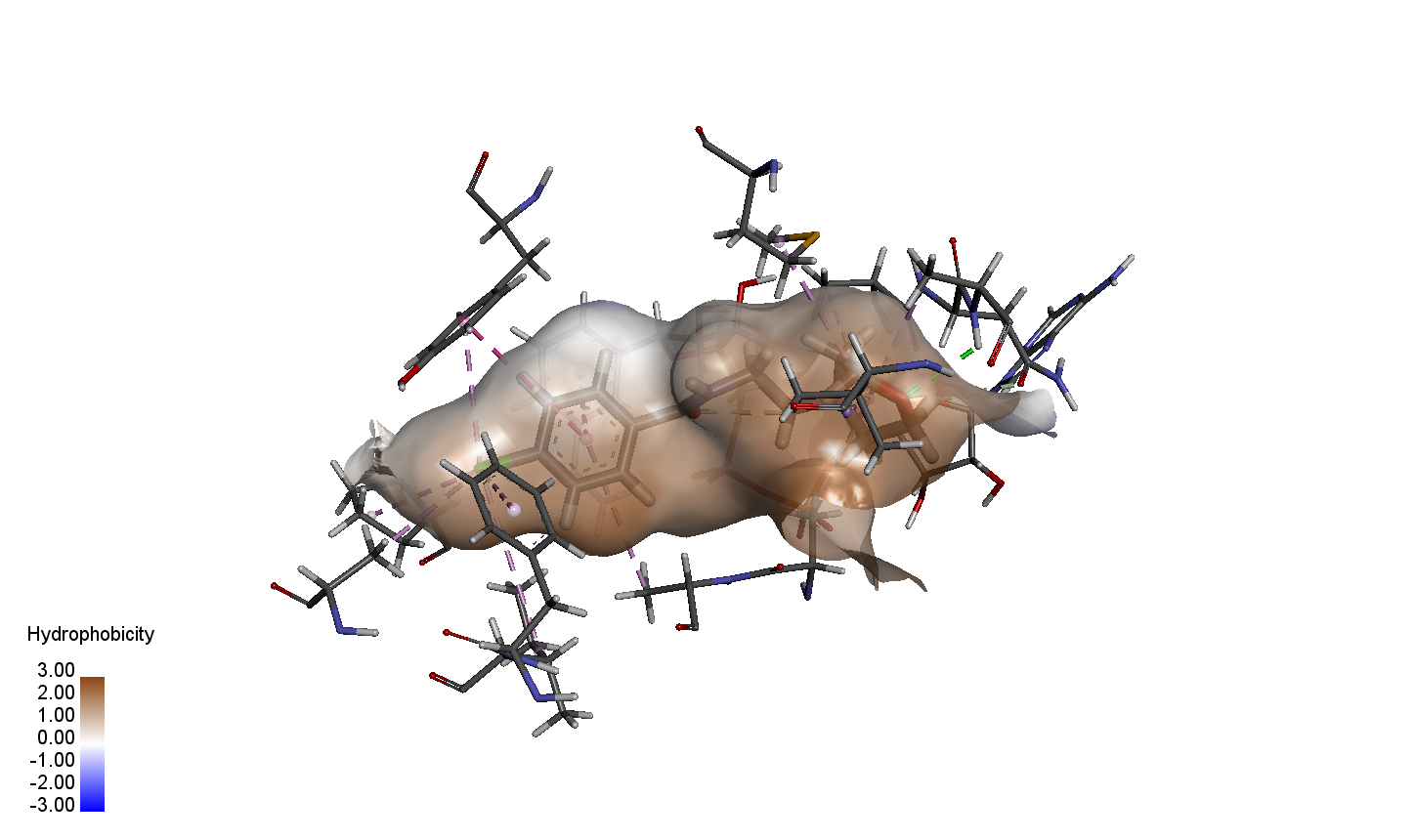


Figure S5 Moclobemide binding in triclosan hydrophobic binding site. A surface colored with the hydrophobicity color scheme is created around moclobemide. Color scheme code: brown = hydrophobic, blue = hydrophilic and white = intermediate. Interacting residues are shown. The image is rendered using Discovery Studio Visualizer 2017 R2.


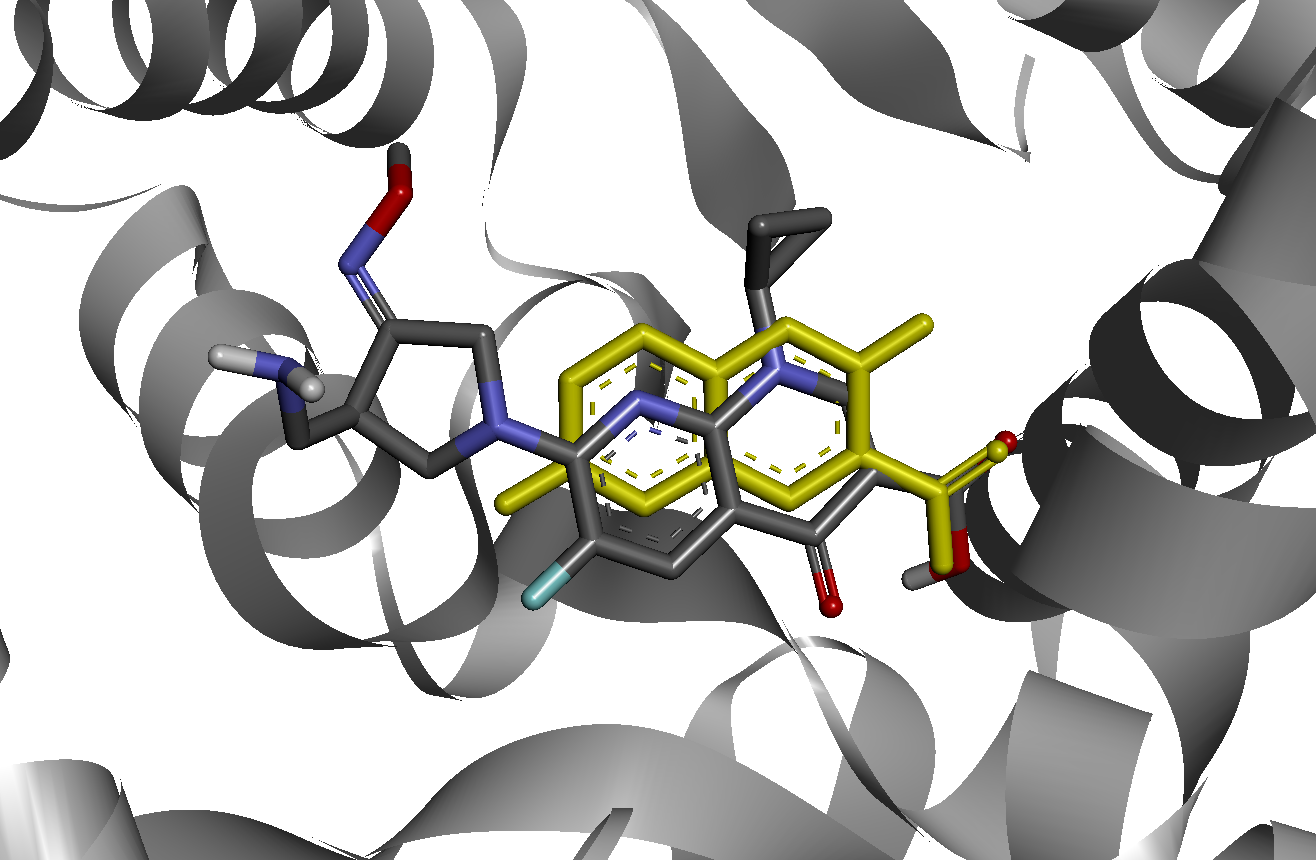


Figure S6 Gemifloxacin (atom type) docked pose with the co-crystallized ligand DHNCA (yellow) in PfLDH binding site. The protein is represented as grey cartoons. The image is rendered using Discovery Studio Visualizer 2017 R2.

**Purine nucleoside phosphorylase (PDB ID: 2bsx) - Inosine (DB00853):** 2bsx is the structure of purine nucleoside phosphorylase crystalized with its natural substrate inosine (NOS1248). Temozolomide is an oral alkylating agent used for the treatment of refractory anaplastic astrocytoma. It is only active when converted at physiologic pH. The compound binds in a polar pocket having as common interacting residues with NOS1248: VAL181 and GLU93 (Figure S8). Its imidazotetrazine ring and inosine purine ring have comparable binding poses (Figure S7). The compound does not share significant similarity with known purine nucleotide phosphorylase inhibitors (ChemBL target ID: CHEMBL5648), the highest Tanimoto similarity being 0.3 (CHEMBL37519).


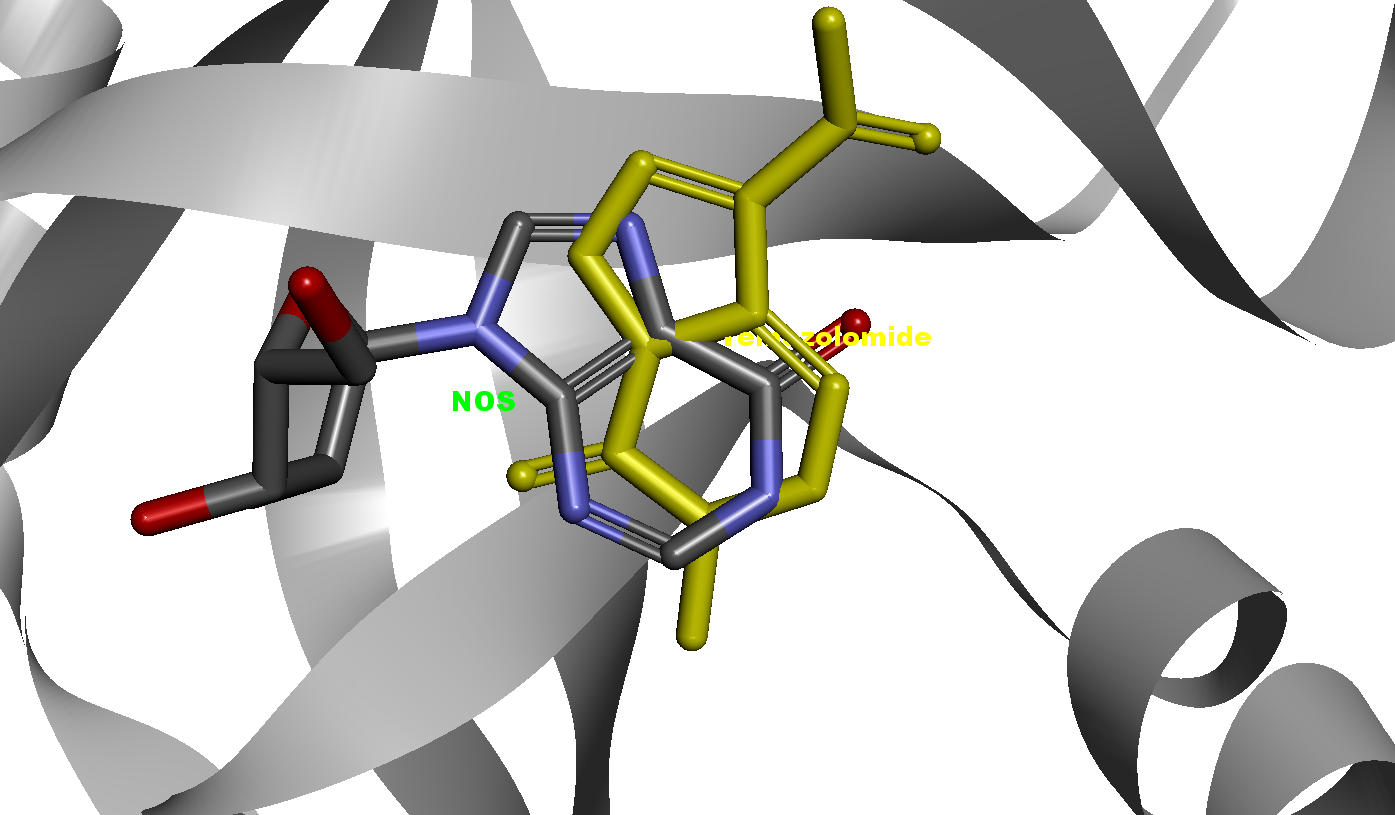


Figure S7 Inosine and NOs1248 binding poses. The image is rendered using Discovery Studio Visualizer 2017 R2.


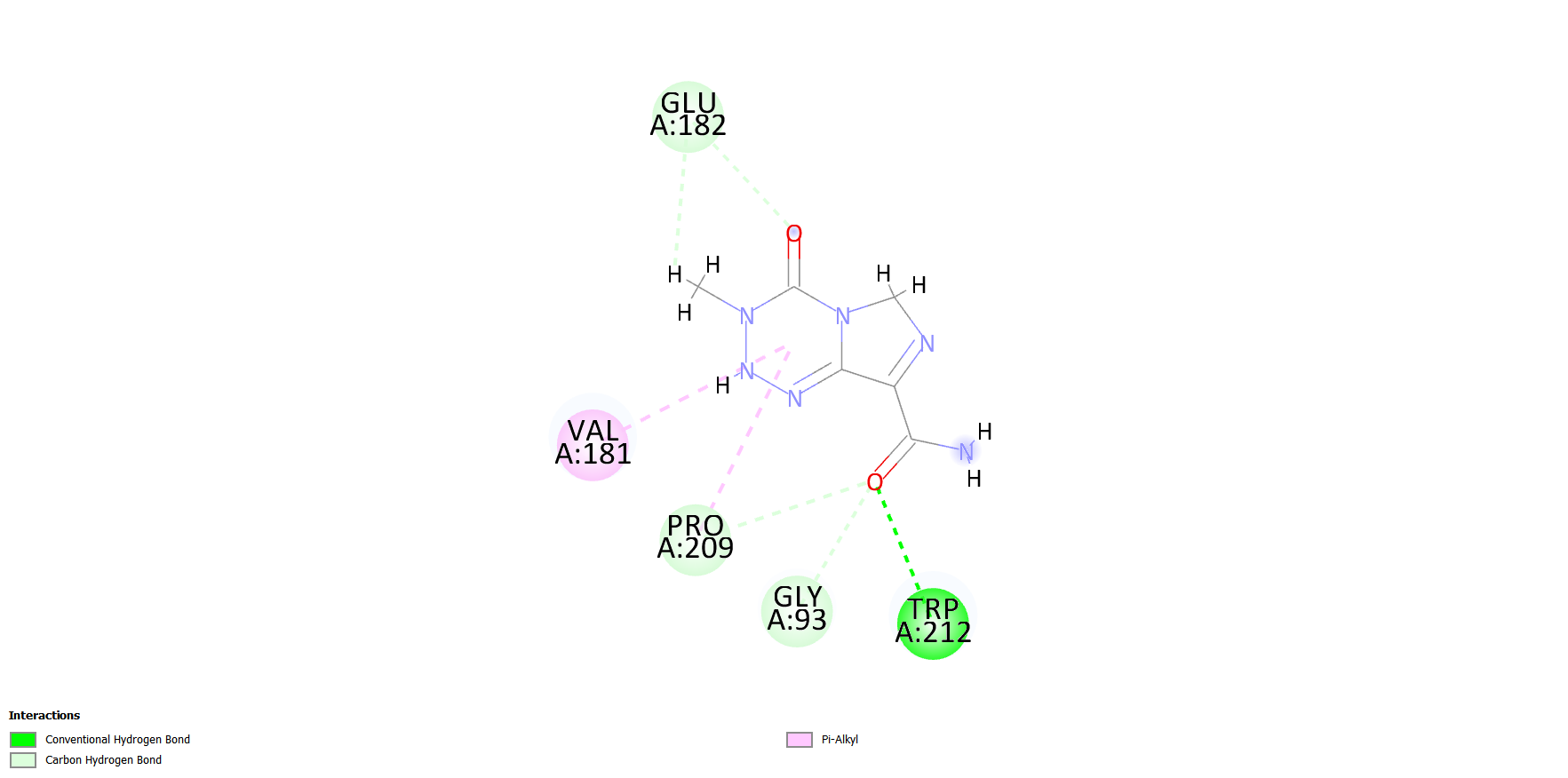


Figure S8 Inosine and 2bsx intermolecular interactions. The image is rendered using Discovery Studio Visualizer 2017 R2.

**Plasmepsin 2 (PDB ID: 2igx) - Fingolimod (DB08868):** Plasmepsin 2 belongs to a family of aspartic proteinases involved in the process of haemoglobin degradation for the parasite, crystallized here with a potent achiral inhibitor (HET CODE: A1T)^2^. Fingolimod modules sphingosine 1-phosphate receptor activity and is indicated for the treatment of relapsing-remitting multiple sclerosis. The compound binds at A1T binding site where it is buried in a hydrophobic pocket, interacting with ASP121 though one of its hydroxyl group and forming a hydrogen bond in the depth of the pocket. Fitting in that pocket showed to be crucial for high-affinity inhibitors^2^. Fingolimod has its polar groups (hydroxyls and amino) fitting in the depth of the depth of the hydrophobic pocket which may have associated energetic cost^3^. On the contrary, A1T presents a long aliphatic chain in it. Through its aromatic ring, fingolimod makes hydrophobic contacts with TYR77, PHE111, ILE123 and VAL82, and has Pi-Pi interactions with TYR77 and PHE111 (Figure S9). The carbon chain engages in alkyl contacts with PHE120 and PHE111 (Figure S10). Also, unlike A1T, fingolimod which presents a different scaffold (Tanimoto similarity 0.22) does not show a large expansion in the vast trench area outside the pocket. Though this region may offer an ideal possibility for scaffold growing. Halofantrine is a common antimalarial targeting Plasmepsin 2^4^.


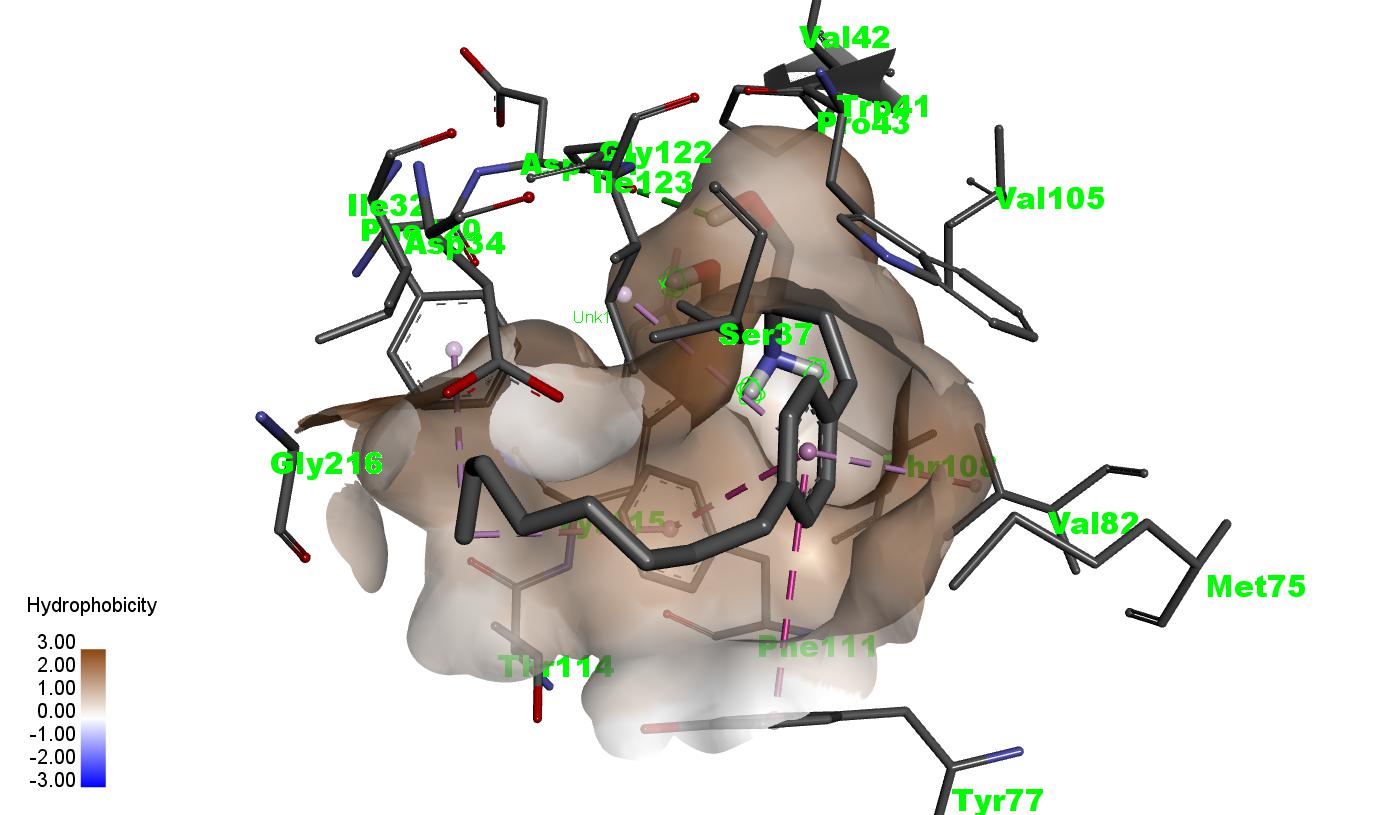


Figure S9 Fingolimod binding pose. The image is rendered using Discovery Studio Visualizer 2017 R2.


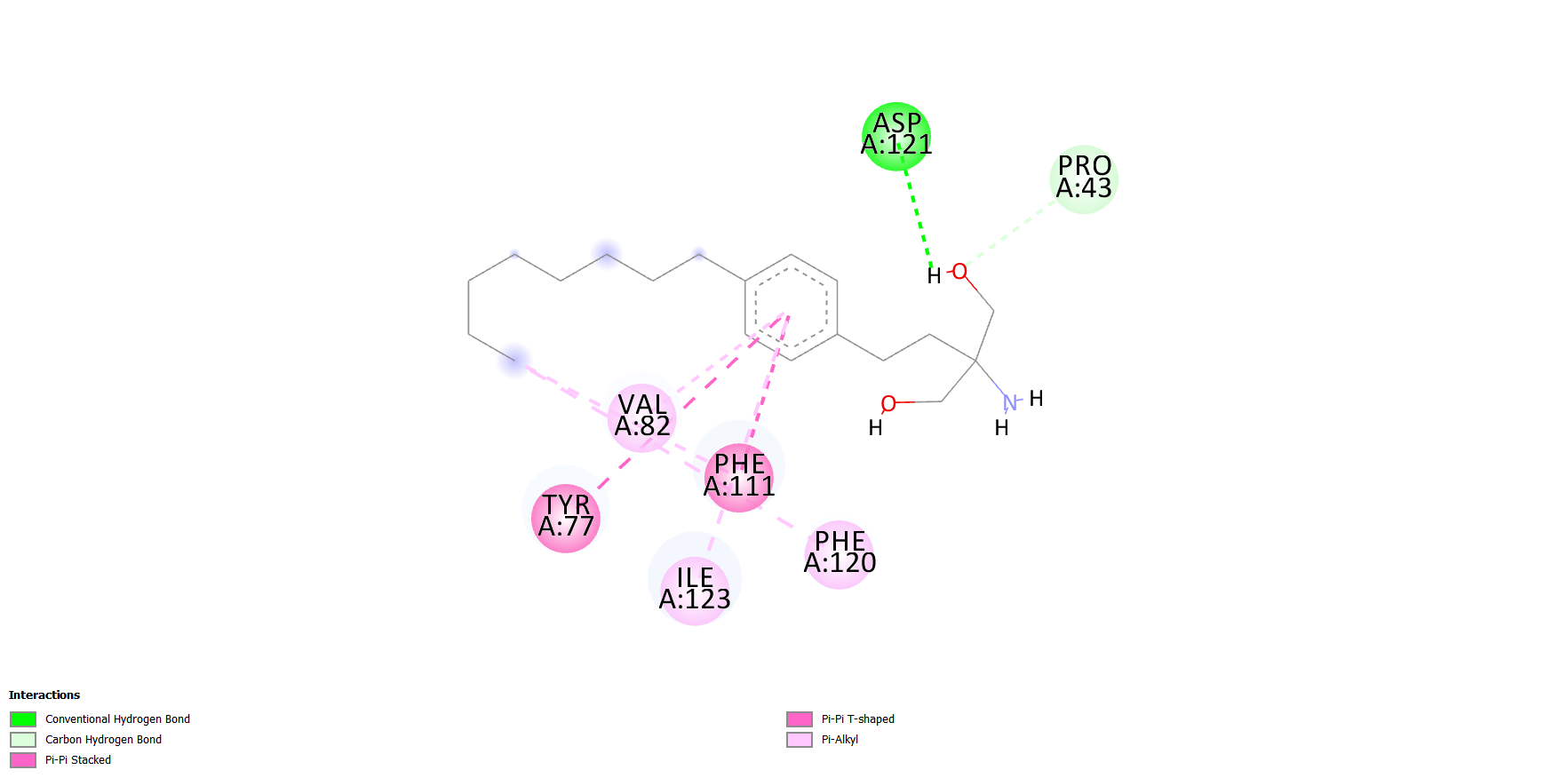


Figure S10 Fingolimod interactions. The image is rendered using Discovery Studio Visualizer 2017 R2.

**Histo-aspartic protease (PDB ID: 3fnu) - Darifenacin (DB00496):** Like 2igx, 3fnu is also a Plasmepsin which also participates in human haemoglobin degradation. Its structure has a unique feature with an active site containing several significant deviations from the standard pepsin-like aspartic proteases PMs (PMI, PMII, and PMIV), despite the high sequence identity (60%)^5^. The protein here is in its closed conformation complexed with KNI-10006 and a Zn ion coordinated by HIS32, ASP215 and GLU278. Darifenacin is an antagonist muscarinic acetylcholine receptor indicated for urinary incontinence. The docked compound interacts with HIS32 among the active site metal coordinating residues which could disrupt the metal coordination sphere. It also shows a comparable binding mode to KNI-10006, interacting with PHE111, ALA217, ILE80, TRP39, VAL120, VAL12 through its aromatic rings (Figure S11). The compound has low structural similarity with KNI-10006 (Tanimoto similarity:0.33). It does not show any polar contact, making only hydrophobic contacts through its rings, well suited for the highly hydrophobic binding site (Figure S12). Interestingly, KNI-10006 presents many polar groups, not involved in any interactions and tend to be oriented toward a more exposed area of the binding site. To date, KNI-10006 is the only reported inhibitor for the target in ChEMBL^6^ (ChembL ID: CHEMBL6075).


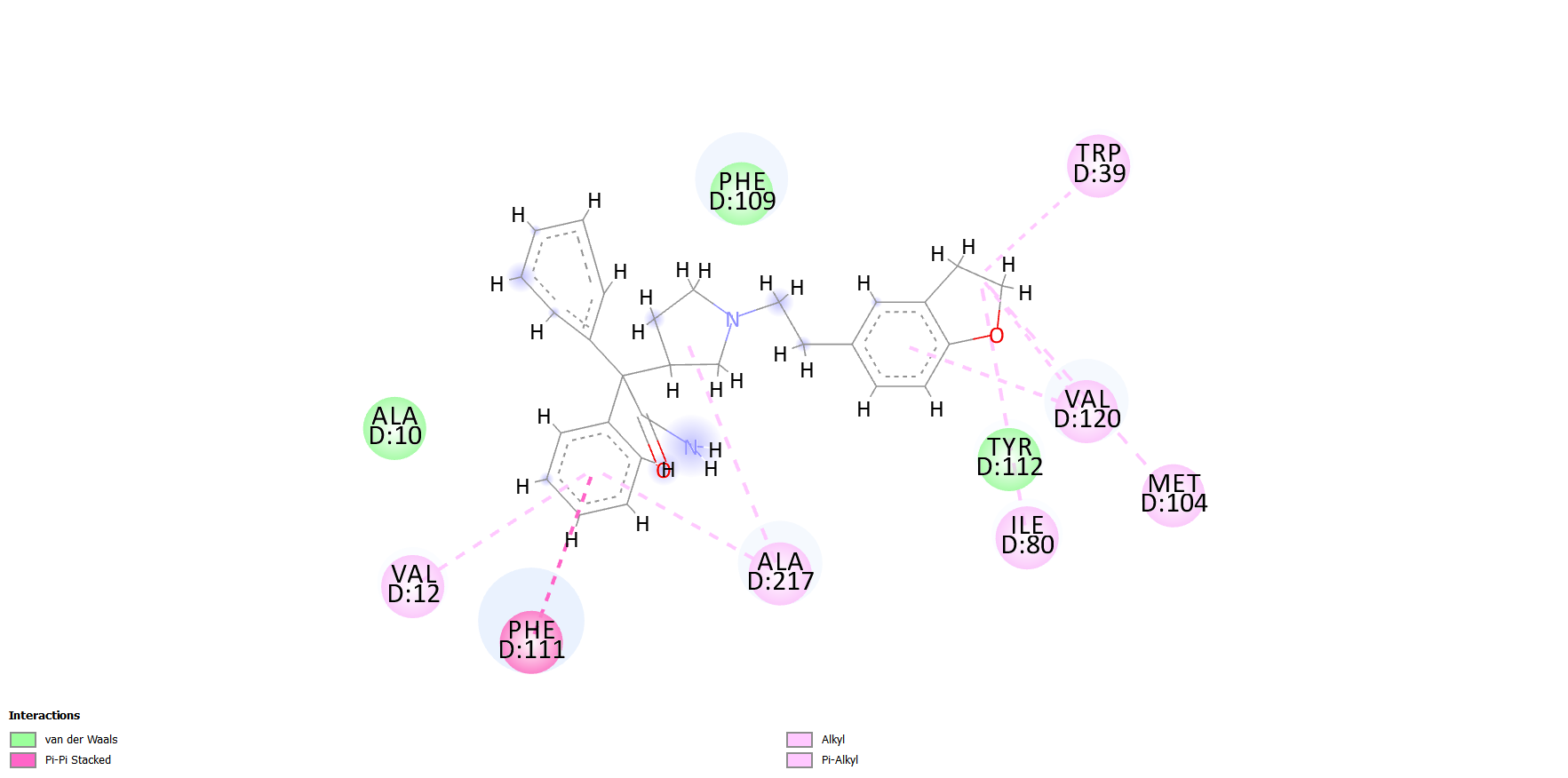


Figure S11 Darifenacin interactions. The image is rendered using Discovery Studio Visualizer 2017 R2.


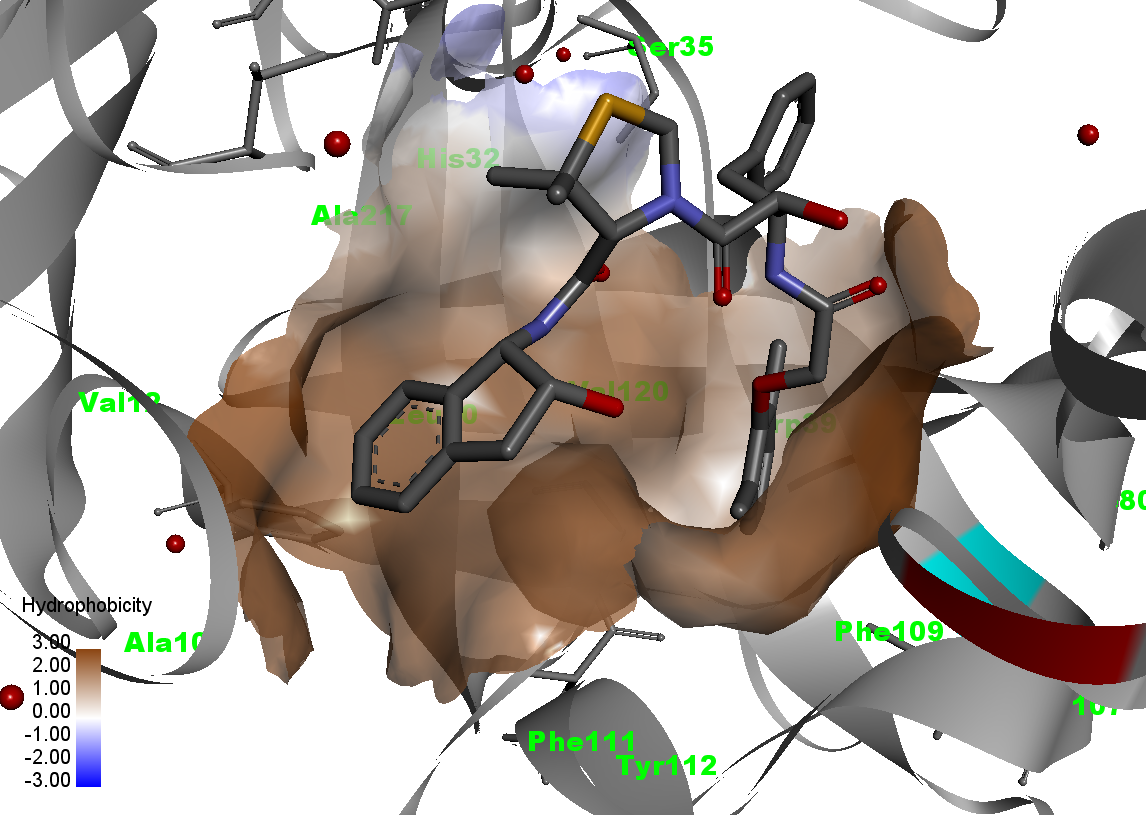


Figure S12 KNI_10006 binding pose. The image is rendered using Discovery Studio Visualizer 2017 R2.


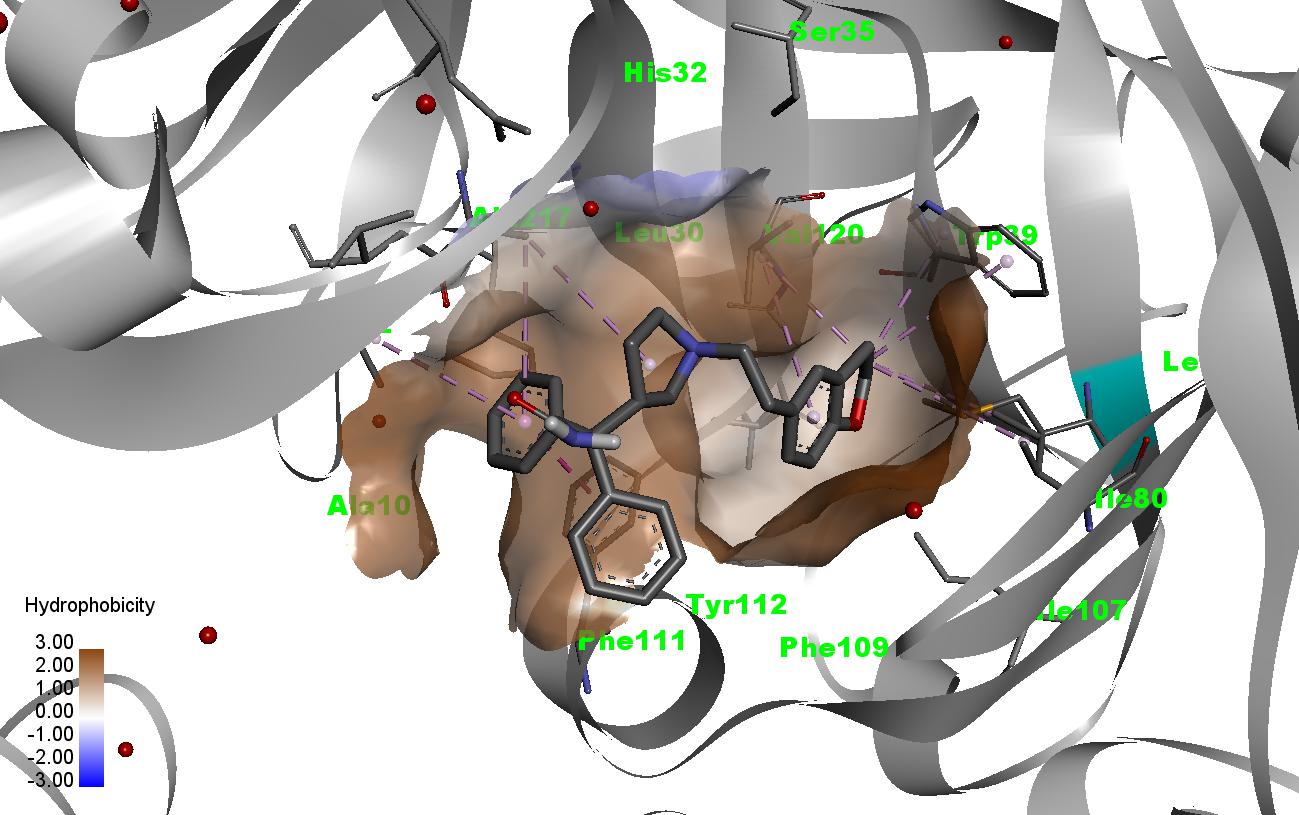


Figure S13 Darifenacin binding pose. The image is rendered using Discovery Studio Visualizer 2017 R2.

**Dihydroorotate dehydrogenase (quinone) mitochondrial (PfDHODH, PDB ID: 3o8a) - DB01203 (Nadolol):** PfDHODH, Atovaquone molecular target^7^, catalyzes the rate-limiting step for pyrimidine synthesis. It showed to be a relevant target for both liver and blood stages in malaria therapy^8^. 3o8a presents the structure complexed here with Genz-667348. Both nadolol and Genz-667348 fit deeply in the binding pocket (Figure S14) and present similar Pi-Sigma interactions with LEU172 and a hydrogen bond with HIS185. PfDHODH inhibitors often showed the H-bond forming with HIS185 and ARG265^9^. The compound also interacts with PHE171 which conformational flexibility allows substituents of variable size to be accommodated into the hydrophobic pocket^9^. The cyclopropyl group on Genz-667348 and the isobutyl group on nadolol both overlap and fit the binding site depth. While the co-crystallized ligand forms a hydrogen bond with ARG265 and makes a hydrophobic contact with PHE188, these interactions are absent in the docked compound which on the other hand interacts with MET536 and CYS175. Nadolol has a significant different scaffold from dihydroorotate dehydrogenase (target ID: CHEMBL3588732) inhibitors, the highest Tanimoto similarity being 0.29 (CHEMBL3689083). Regarding Genz-667348 selectivity for PfDHODH, LEU46 and MET43 in human DHODH are substituted for CYS175 and LEU172 in PfDHODH while Nadolol also interacts with these two later residues, good indicator for its selectivity.


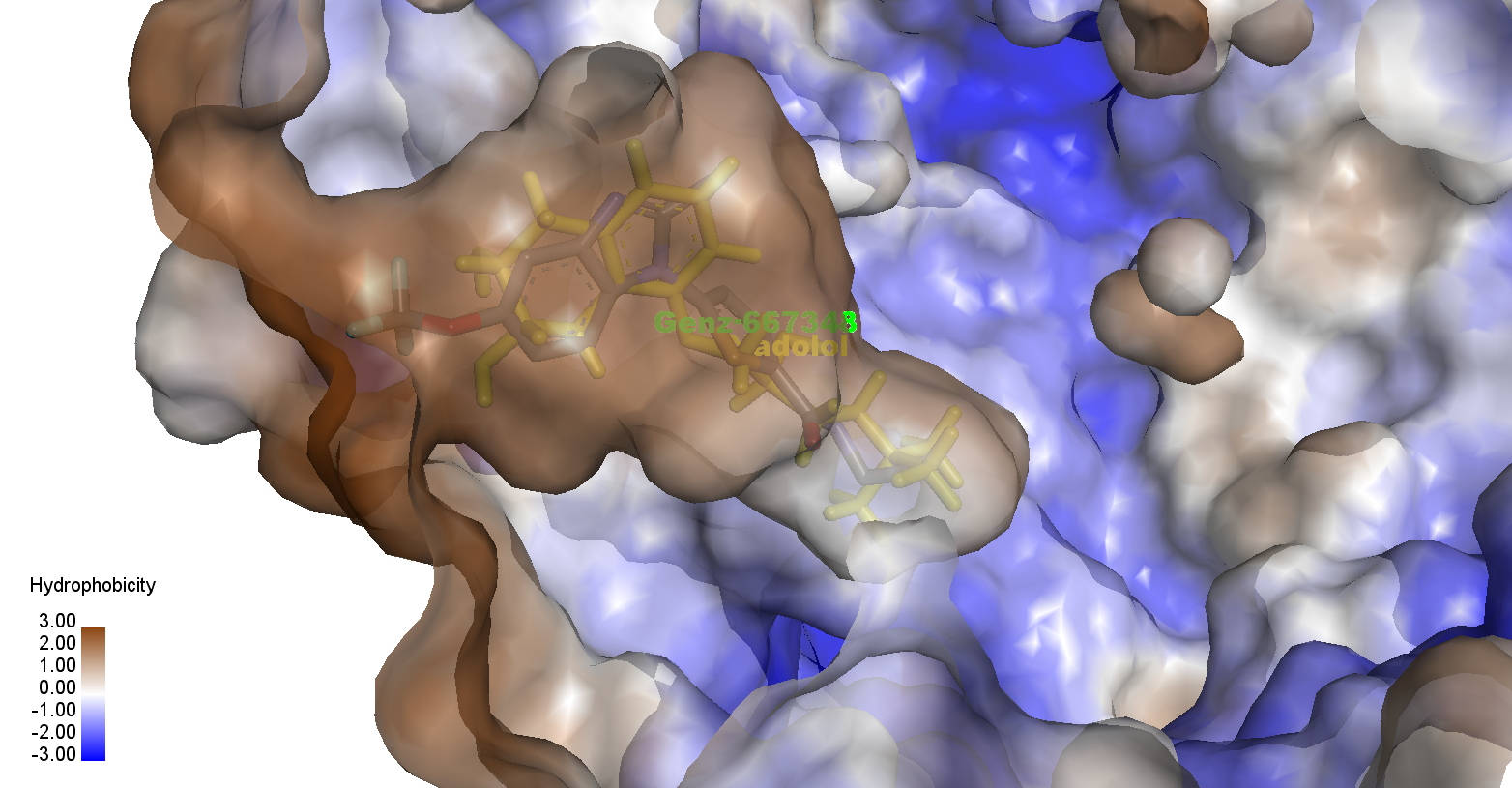


Figure S14 Nadolol Genz-667348 binding poses: Nalodol in yellow. Genz-667348 in atom type. The image is rendered using Discovery Studio Visualizer 2017 R2.

**Phosphoethanolamine N-methyltransferase (*PfPMT*, PDB ID:3uj8) - Abacavir (DB01048):** PfPMT is the target for amodiaquine^10^ and is involved in malaria lipid biosynthesis and do not possess human homolog^11^. Here the structure is crystallized with an inhibitor: Sinefungin. Abacavir and Sinefungin in their binding modes have their purine rings showing the same pose and interaction pattern (Figure S16). They both form hydrogen bonds with ILE86, ILE111, ASN109, and ASP10. Except for a nitrogen on its purine ring, all Abacavir polar atoms are involved in hydrogen bonding (Figure S15). The common purine rings also interact with ILE86 and LEU111. Abacavir scaffold is very common among Phosphoethanolamine N-methyltransferase inhibitors and has a Tanimoto similarity of 0.35 with Sinefungin. However, the latter makes efficient use of its polar groups to make more hydrogen bonds especially on its dihydroxyoxolan ring and following amine and acid groups. Its high LipE (8), low MW (286 Kda) and a predicted clogP of 1.09 leaves room for scaffold growing and optimization although this may be challenging as the compound fully occupies the pocket. Abacavir may serve as starting for repositioning as antimalarial and it may be interesting to study Sinefungin on Abacavir antiviral target. Though the compound was reported to be inactive in a screen for delayed death inhibitors of the malarial parasite plastid (ChEMBL assay IDs: CHEMBL1201862), the target here, phosphoethanolamine methyltransferase, is located in the Golgi apparatus^12^.


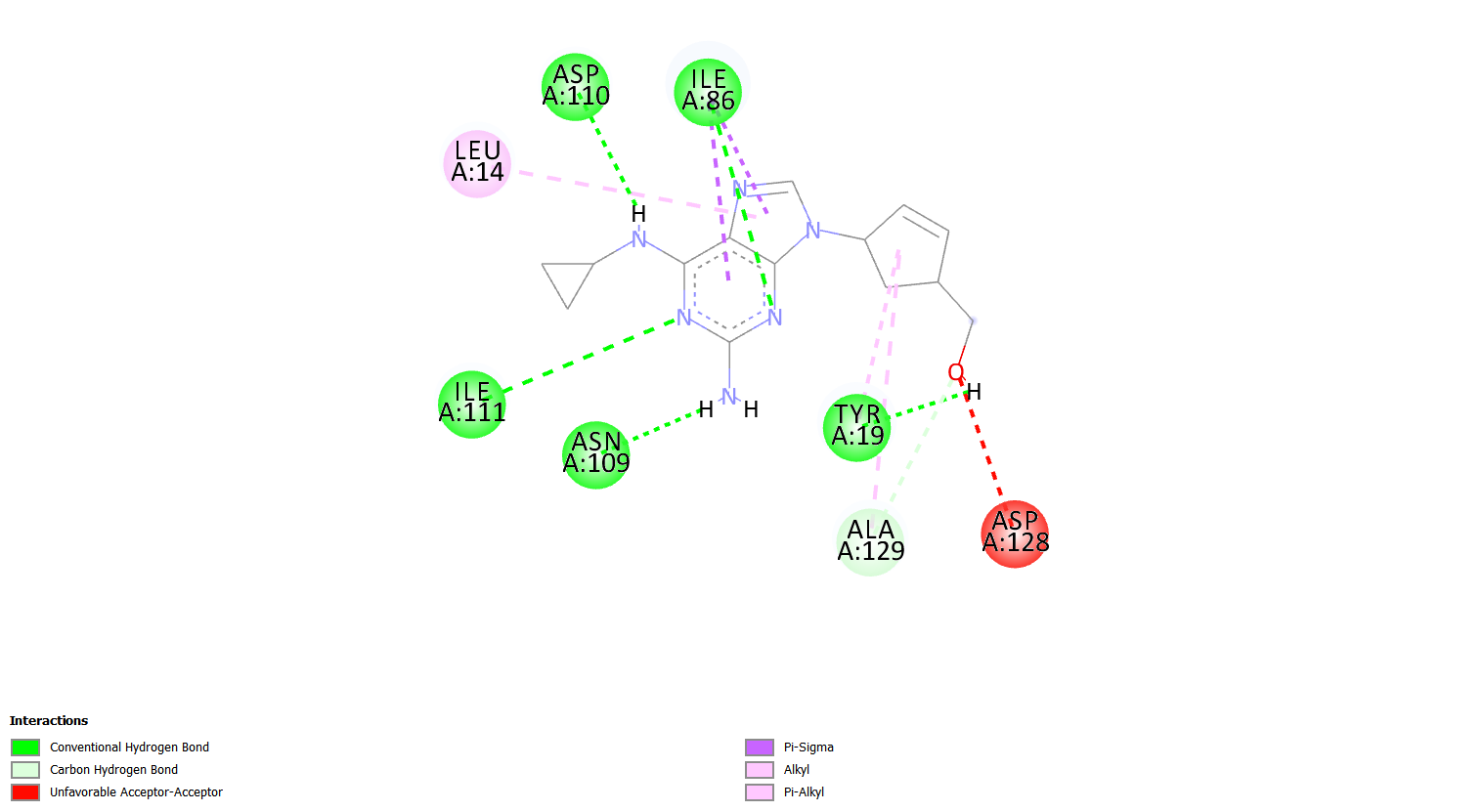


Figure S15 Abacavir interactions. The image is rendered using Discovery Studio Visualizer 2017 R2.


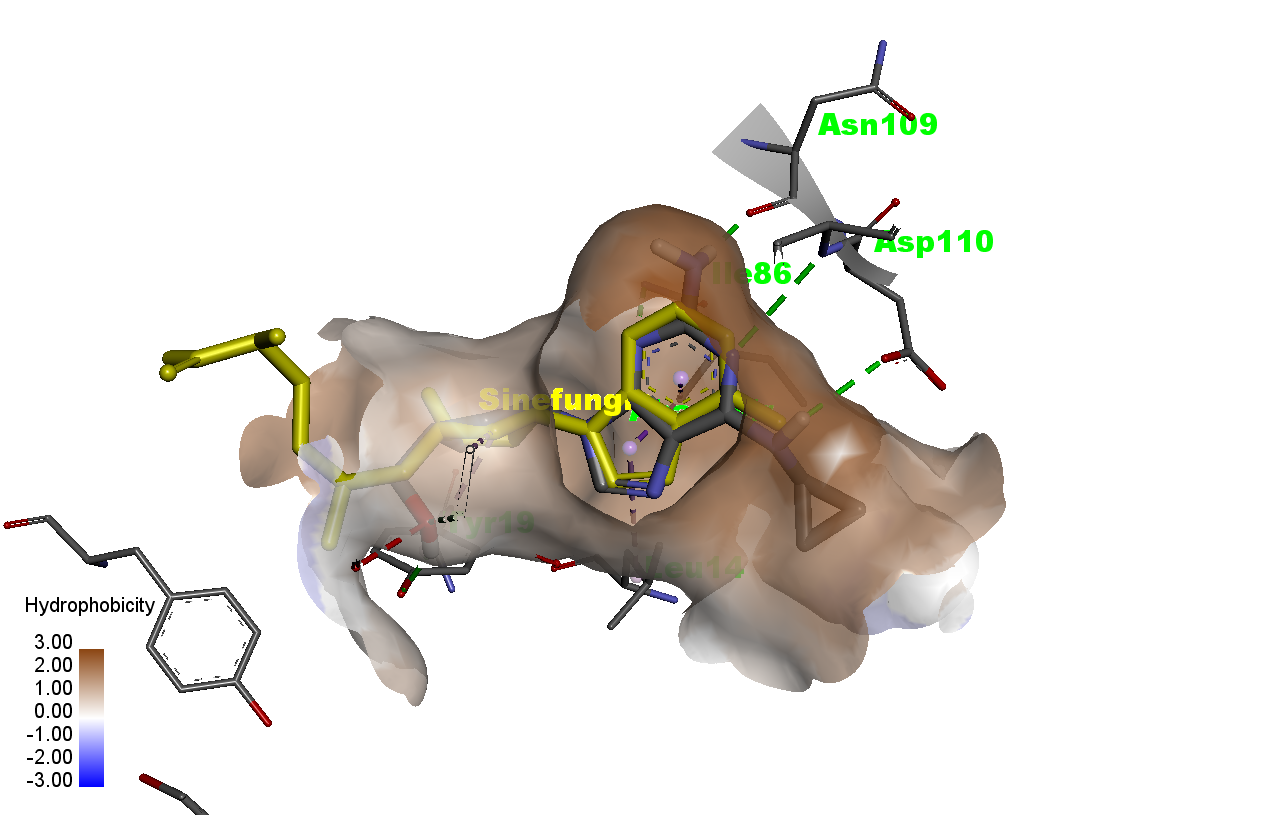


Figure S16 Abacavir binding pose

**Choline kinase (PDB ID: 3fi8) - Tenoxicam (DB00469):** Choline kinase (CK) catalyzes the first phosphorylation reaction in the CDP-choline pathway for the synthesis of phosphatidylcholine and phosphatidylethanolamine. Inhibition of the enzymes of the pathway arrest parasite’s growth and cures malaria in a mouse model^13,14^. Tenoxicam is an anti-inflammatory agent with analgesic and antipyretic properties, used to treat osteoarthritis and control acute pain. The crystal structure has a cofactor ADP and a MG ion in the active site. Tenoxicam binds a polar pocket, OP603 binding site and overlaps well with this latter but also extends outside the pocket as it is a larger compound. The best Grim pose (Grscore 0.74) binds to TRP395, ASP288, ARG399, TYR329, ASP305, GLY114, GLU307, THR116, TYR308, GLN290, ASP305 the cofactor ADP and the MG ion in the active site (Figure S17). Similar interactions with TYR329, GLU307 and TRP395 were observed when two potent inhibitors BR23 and BR25 were docked on 3fi8^14^. Though these compounds do not interact with the metal ion and their structures differ from tenoxicam which is also significantly different from OP603 (Tanimoto similarity 0.35).


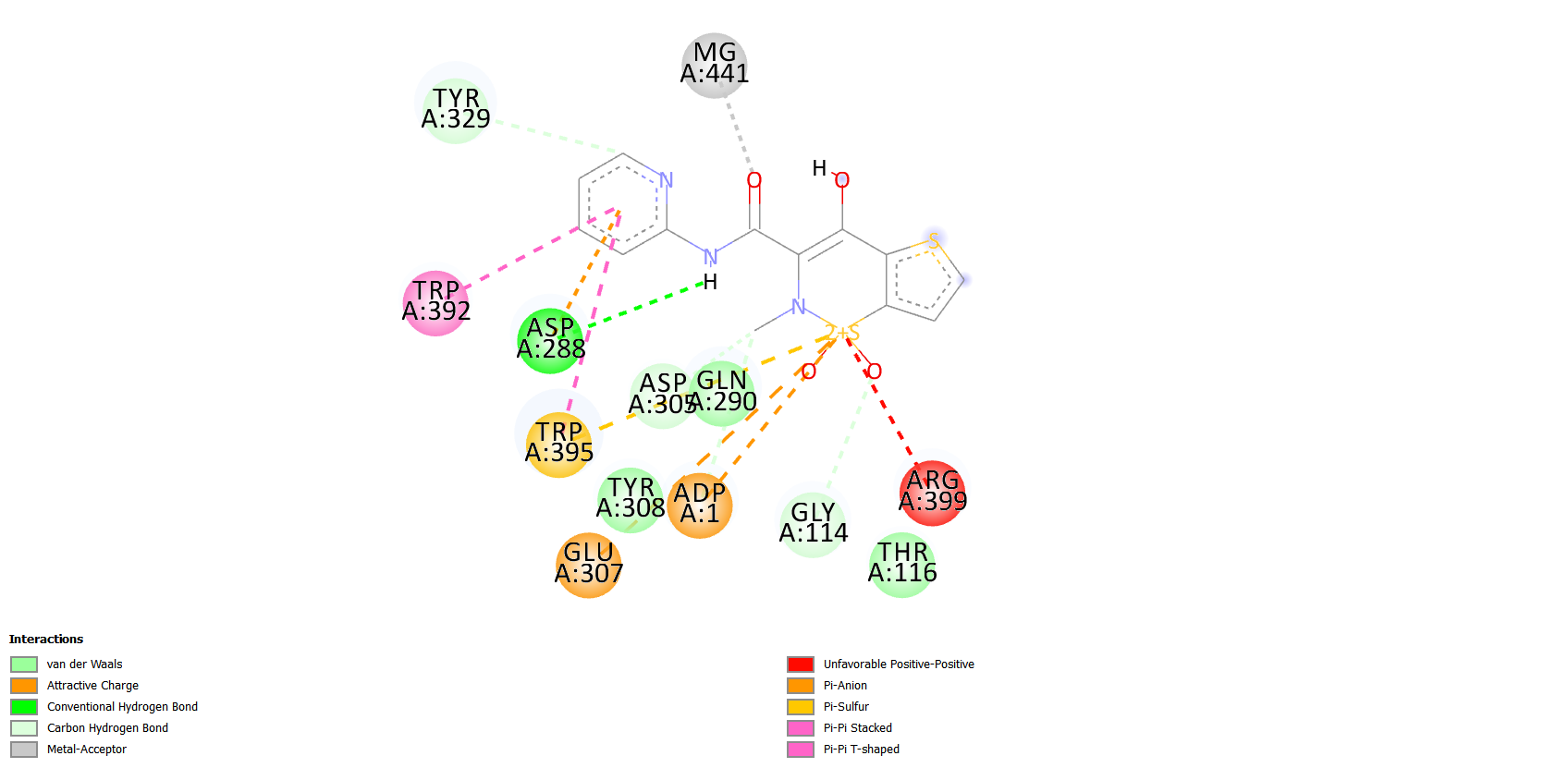


Figure S17 Tenoxicam interactions. The image is rendered using Discovery Studio Visualizer 2017 R2.

**GTPase rab6 (PDB ID: 1d5c) - Isosorbide dinitrate (DB00883):** 1d5c is the structure of rab6 nucleotide-binding domain, a protein involved in intracellular vesicle trafficking, bound to Guanosine-5’-Diphosphate (GDP)^15^. Isosorbide dinitrate is a vasodilator used in the treatment of angina pectoris^16^. The compound is rather polar with a predicted negative cLogP of -1.06 and a PSA of 123 Å2 resulting in a low SEI score 7. However, it binds a polar pocket (Figure S18) with a similar binding mode to GDP diphosphate group. Its two nitrate groups make charged interactions with LYS24 and LYS125 on the two ends of the compound (Figure S19). It does not interact with SER26, THR25 which coordinate the magnesium ion^15^. SER26, THR25, VAL22, GLY23 and SER42 form a total of six hydrogen bonds with the oxygens groups on its tetrahydrofuran rings. The same interactions are present in Guanosine-5’-Diphosphate (GDP) which in addition interact with the MG atom and numerous other interactions on its purine ring. The hit compound could be expanded into that region where GDP purine ring forms Pi-sigma with LYS125, Pi-Pi T-shaped interactions with PHE36 and also engages in hydrogen bonding with ALA155, LYS156 and ASP127. To date, no active compound against the protein (UniProt ID: Q26000) was reported in ChEMBL^6^.


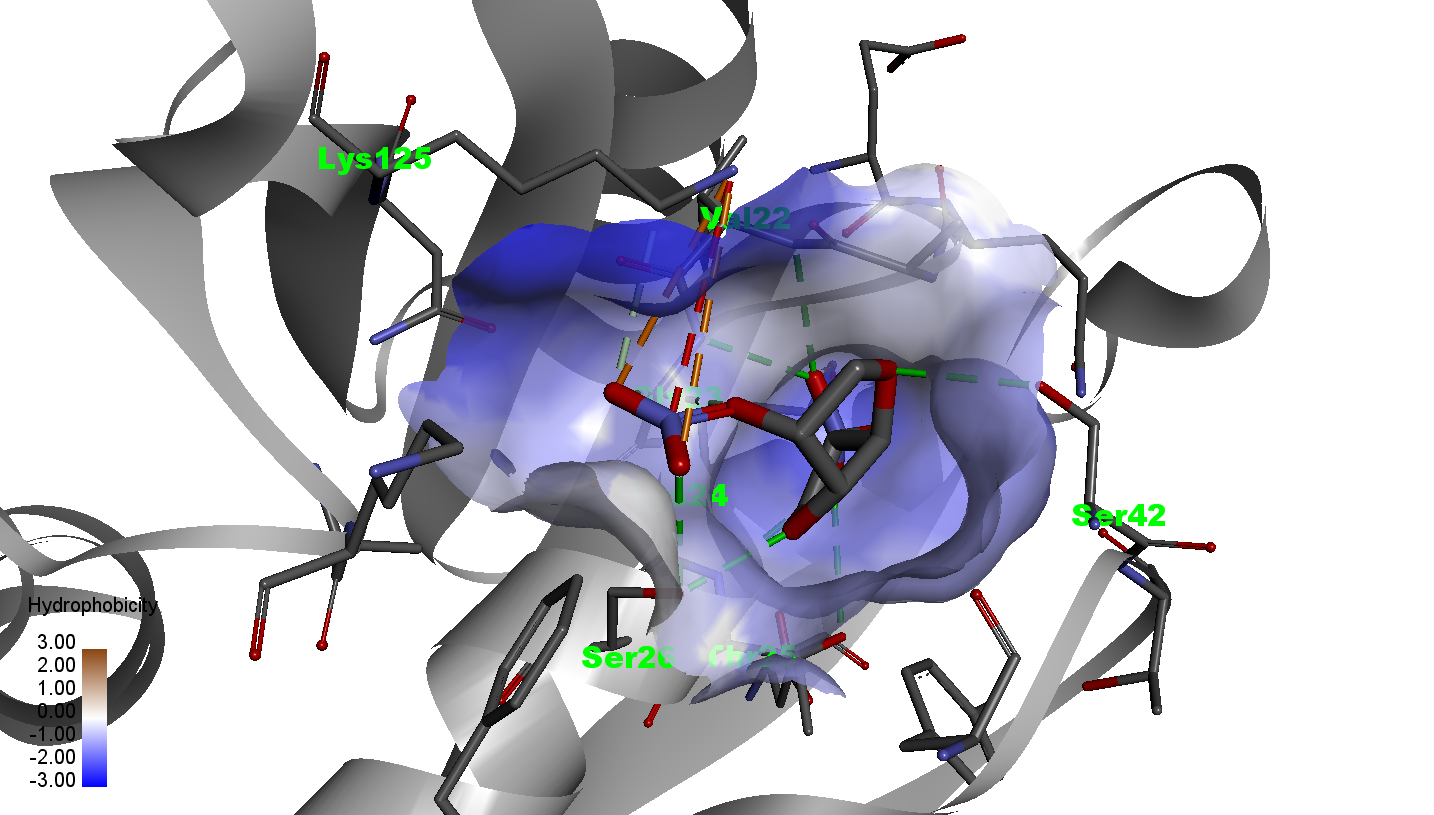


Figure S18 Isosorbide dinitrate binding mode. The image is rendered using Discovery Studio Visualizer 2017 R2.


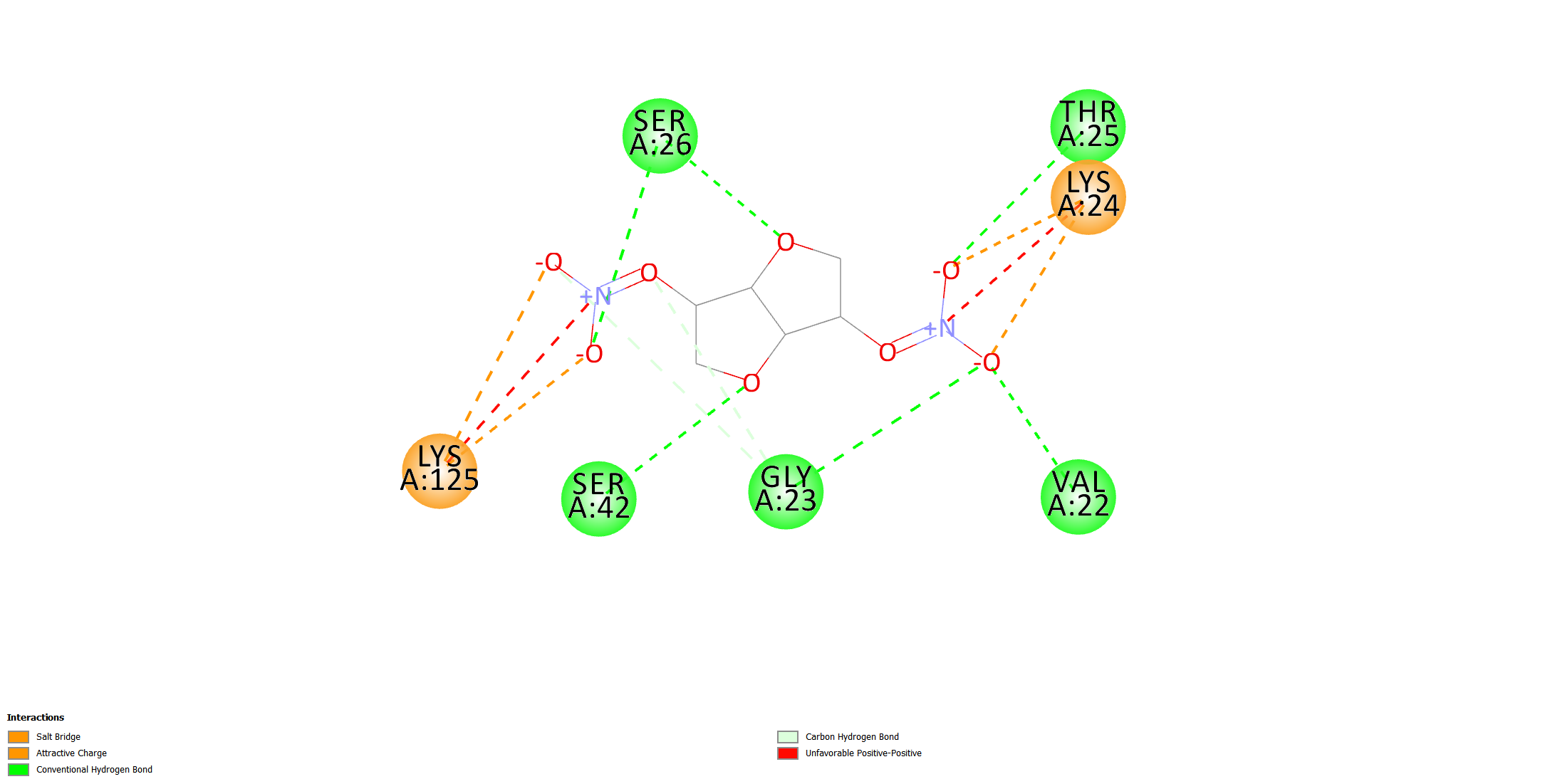


Figure S19 DB00883 interactions. The image is rendered using Discovery Studio Visualizer 2017 R2.

**Ferredoxin–NADP reductase (PDB ID: 3jqr) - Grepafloxacin (DB00365):** Ferredoxin reductase is an apicoplastic protein which produces reduced ferredoxin for essential biosynthetic pathways in the apicoplast^17^. Grepafloxacin is an oral broad-spectrum fluoroquinolone antibacterial. It presents several common interacting residues with the large Flavine Adenine Dinucleotide (FAD) the co-crystalized ligand (ARG101, GLU314, HOH317, LEU102, SER104, THR53, TYR103, TYR316). A noteworthy difference in the binding is FAD extension toward a more polar region of the binding site notably through its phosphate groups. While grepafloxacin only fit in the hydrophobic region of the pocket in which it 4-quinolone core and FAD quinoxaline ring show very similar binding mode (Figure S20). Evaluating 25 quinolones and fluoroquinolones for their antimalaric activity, Mahmoudi et al. found that only grepafloxacin had an IC50 in the 10–15 micro molar range^18^. The compounds has activity against liver and blood stage *Plasmodium spp*^19^. Several studies established the compound antimalaric activity^19–21^. Tafenoquine a *Plasmodium* liver stage active drug is hypothesized to act through ferredoxin-NADP+ reductase. Indeed, 8-aminoquinolines, tafenoquine derived metabolites are redox cycled by *P. falciparum* ferredoxin-NADP+ reductase and diflavin reductase enzymes generating hydrogen peroxide and hydroxyl radicals which eventually kill the parasite^22^. One can expect grepafloxacin to also be liver stage active.


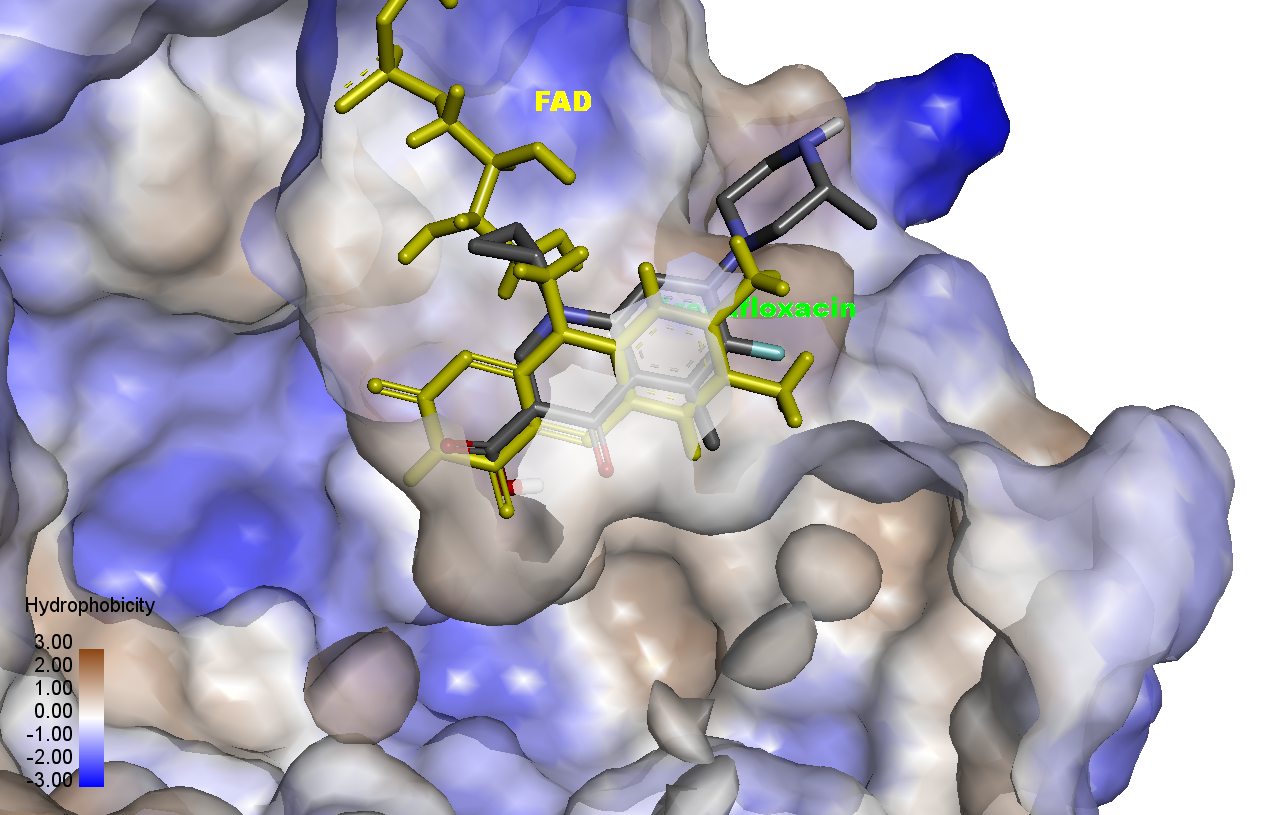


Figure S20 FAD and Grepafloxacin binding poses. The image is rendered using Discovery Studio Visualizer 2017 R2.

**Thioredoxin reductase (PfTrxR, PDB ID: 4j56) - Prazosin (DB00457):** 4j56 is the first thioredoxin reductase-thioredoxin structure complexed with its substrate and the prosthetic group FAD. The enzyme is essential for *Plasmodium falciparum* and is involved in redox homeostasis^23^. It was identified as a putative liver stage target^24^. Prazosin is a selective $\alpha$-1-adrenergic receptor antagonist used to treat hypertension, binding in FAD binding site in a complete buried pocket. It interacts with some water molecules, ALA191, ALA369, ASP357, CYS88 CYS93, GLY52, LYS96, PRO51, SER212, THR87, VAL233 among which CYS93, CYS88 form the redox centers for the protein function^23^. The compound also forms hydrogen bonds with LYS96 and ASP357. FAD binding pocket is large with a mitigated polar character, in which prazosin occupies a large portion. Its scaffold is characterized by an aromatic heteropolycyclic also common on *Plasmodium* thioredoxin reductase inhibitor (CHEMBL4547). The two rings present numerous hydrophobic interactions. The compound has a high lipophilic efficiency (9), a molecular weight of 383 and BEI of 29. Interestingly, prazosin was found to be inactive in a mammalian thioredoxin reductase (Pubchem^25^ BioAssay ID 488772, 488773, 588453) good indication of its selectivity profile.

**Cell division control protein 2 homolog (PfPK5, PDB ID: 1v0o) - Anastrozole (DB01217):** PfPK5 is the first structure of a *P. falciparum* protein kinase (CDK) with a selective and potent ATP-competitive CDK inhibitor^26^. The protein is co-crystallized with indirubin-5-Sulphonate (INR) which anastrozole occupies the hydrophobic binding site. Anastrozole is an aromatase inhibitor used in the treatment of breast cancer and also can be used as an adjuvant. The compound has good binding energy (-10.1 Kcal/mol) and lipE (7.5) with a Grscore of 0.69. It molecular properties (MW:293 logP:2.9 PSA:78) allow for optimization. It has two carbonitriles functions and makes numerous alkyl interactions on its benzene ring with ILE10, VAL18, VAL63, ALA142, ALA30, ASP85 and PHE79. These residues were previously reported as interacting with an inhibitor: 6-cyclohexylmethoxy-2-(4-sulfamoylanilino) purine (NU6102)^26^. The compound also forms a hydrogen bond with LEU132 and pi-anion with LYS32. LYS32 orientates the ATP $\alpha$ and $\beta$ phosphate groups for phosphotransfer and INR interacts with PHE79, GLU80, LEU82, LYS32^26^. Anastrozole structure is significantly different from INR (Tanimoto similarity of 0.14) and also from other PfPK5 inhibitors from CHEMBL (Target ID: CHEMBL2189145), the highest Tanimoto similarity being 0.47.

**Thymidylate kinase (PfTMPK, PDB ID: 2yoh) - Salbutamol (DB01001):** PfTMPK is an enzyme of the dTTP synthesis pathway for DNA synthesis and catalyzes the phosphorylation of thymidine 5’-monophosphate (dTMP) to form thymidine 5’-diphosphate (dTDP. ). Due to its implication in DNA synthesis, the target is likely to be essential for the parasite liver stage^27,28^. Its crystal structure here is complexed with an inhibitor, urea-alpha- deoxythymidine. Salbutamol is a short-acting, selective beta2-adrenergic receptor agonist used in the treatment of asthma and chronic obstructive pulmonary disorder (COPD). The compound binds in the active site with its benzene ring having a comparable pose to urea-alpha-deoxythymidine and makes hydrophobic interactions with PHE74 and hydrogen bond with ARG78, GLU151 and ARG78. Compared to other PfTMPK (NCBI Protein: Q8I4S1) inhibitors’ structures, salbutamol presents a different scaffold, the most similar active compounds having a Tanimoto similarity of 0.41 (PubChem cid: 71457249). From its binding pose, salbutamol scaffold can be extended toward the more exposed surface of the binding site. This region may present good potentiality for good interaction as urea-alpha-deoxythymidine also extends in that area. Its low MW and logP (MW 239 logP 1.3 with a single aromatic ring) allows for developability. The compound was previously reported for its antimalarial activity^11^.

**Calcium-dependent protein kinase 2 (PfCDPK2, PDB ID: 4mvf) - Abiraterone (DB05812):** 4mvf is the Structure of *Plasmodium falciparum* CDPK2 complexed with an inhibitor, staurosporine. Absent from vertebrates, PfCDPK2 presents an interesting target for antimalarial therapy^29^. Abiraterone is a steroid and an innovative drug that offers clinical benefit to patients with hormone refractory prostate cancer. Abiraterone binds in a trench-like hydrophobic site and interacts with ALA99, ASP213, CYS149, ILE212, LEU199, LYS101, MET146, VAL130, VAL86. The compound presents a hydrophobic character (low PSA: 33.12 with high logP: 5.39) with interactions dominated by alkyl contacts. A polar contact exists with THR82 on a more exposed area of the binding site. It does not share significant similarity with the enzyme (Target ID CHEMBL1908387) inhibitors, the highest being 0.5 for CHEMBL602580.

**Protein kinase 7 (PFPK70, PDB ID:2pmn) - Lamotrigine (DB00555):** 2pmn is the structure of PFPK7, known as an “orphan” kinase with no human homolog complexed with K510^26^ and important for mosquito transmission^30^. Lamotrigine is an antiepileptic drug belonging in the phenyltriazine class used in the treatment of epilepsy and bipolar disorder. The promiscuous compound (15 targets in DrugBank) This co-crystallized ligand, K510, is a competitive ATP inhibitor which binds in the active site cleft and lamotrigine binds in the same site as K510 and interacts with ALA53, ASP190, ILE42, LEU101, LEU179, LYS55, SER189, TYR117 while having hydrogen bonding with GLU118, MET120 and ASP123. Common interacting residues with K510 were MET120, LEU179, LEU101, ILE42, TYR117. Lamotrigine presents a novel scaffold as PFPK7 inhibitors as its highest Tanimoto similarity to other PFPK7 inhibitors (Target ID: CHEMBL6169) was 0.50 for compound CHEMBL602580.

**Bromodomain protein putative (PDB ID:4py6) - Pirbuterol (DB01291):** 4py6 is a bromodomain of *P. falciparum* complexed with a potent inhibitor R78 (PubChem CID: 11364421). The protein showed to be crucial for the coordinate regulation of genes involved in erythrocyte invasion^31^. Pirbuterol is a hydroxypyridine used as a bronchodilator and targetting beta-2 adrenergic. Despite a relatively low binding affinity (-6.7 Kcal/mol), the compound counterbalance with a low clogP (0.7) for a final LipE of 7. Interestingly, pirbuterol has a deeper fit in the binding pocket than R78, with two hydroxyl on its benzene ring making hydrogen bonds with THR1219 and MET1243 (Figure S22). More the pocket doesn’t present a high hydrophobic character in that region which could energetically cost to the hydroxyl groups. R78 doesn’t interact with those two residues but binds on a more exposed area of the pocket. Pirbuterol has good potential for developability as it scaffold can be extended toward the pocket exposed area where R78 is binding. More the compound is rather small with a MW of 240 and low clogP (0.7). Also, it interacts with ASN1273, ASN1273, ASN1278, ASP1244, CYS1274, ILE1227, ILE1231, ILE1287, MET1243, PHE1217, PHE1277, TYR1235, VAL1221 as does R78 with similar residues (Figure S21).


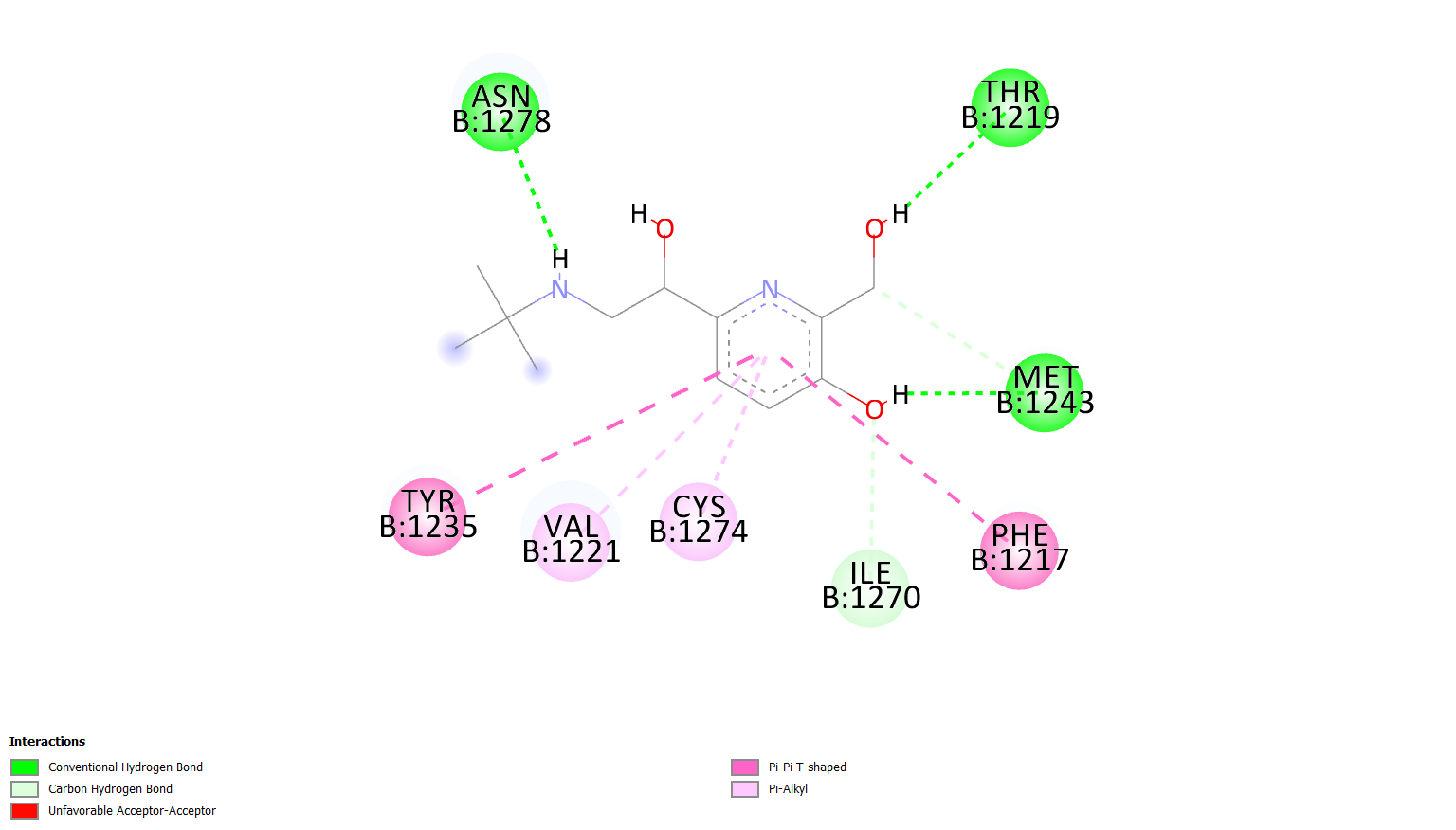


Figure S21 Pirbuterol interactions. The image is rendered using Discovery Studio Visualizer 2017 R2.


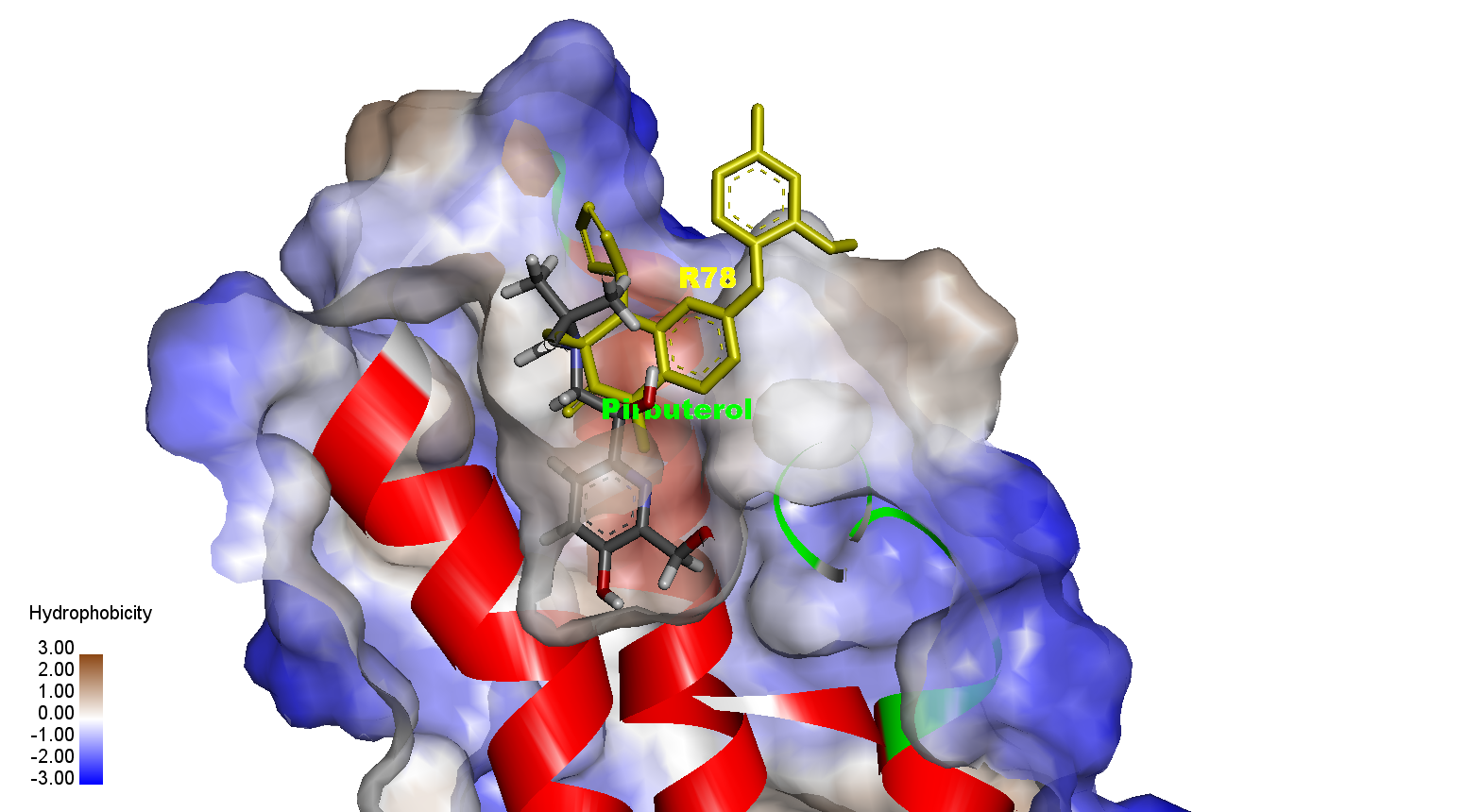


Figure S22 Pirbuterol Binding pose. The image is rendered using Discovery Studio Visualizer 2017 R2.

**Peptide deformylase (PfPDF, PDB ID:1rl4) - Ruxolitinib (DB08877):** *Plasmodium* peptide deformylase (PfPDF) is an apicoplast protein, most likely involved in the deformylation of the amino terminal fMet residue in newly synthesized proteins^32^. 1rl4 is the structure of the metalloenzyme co-crystallized with a submicromolar inhibitor: BRR. Ruxolitinib is a janus-associated kinase inhibitor indicated to treat bone marrow cancer. The drug binds on BRR binding site and interacts with GLN112, GLU154, GLU163, GLU199, HIS198, ILE106, ILE195, LEU157 (Figure S23, Figure S24). A notable difference in the binding modes is ruxolitinib extension outside the binding site forming amide-pi stacking contacts with GLY155 through its pyrrolo(2,3-d)pyrimidine ring. Also, the compound is clashing with GLY107 and ILE106. The two compounds remain structurally different, with a low Tanimoto similarity of 0.14. Therefore, ruxolitinib can present a new structural scaffold for the development of peptide deformylase inhibitors. Ruxolitinib did not interact with any of the cobalt coordinating residues: CYS155, HIS196, and HIS200 nor does BRR.


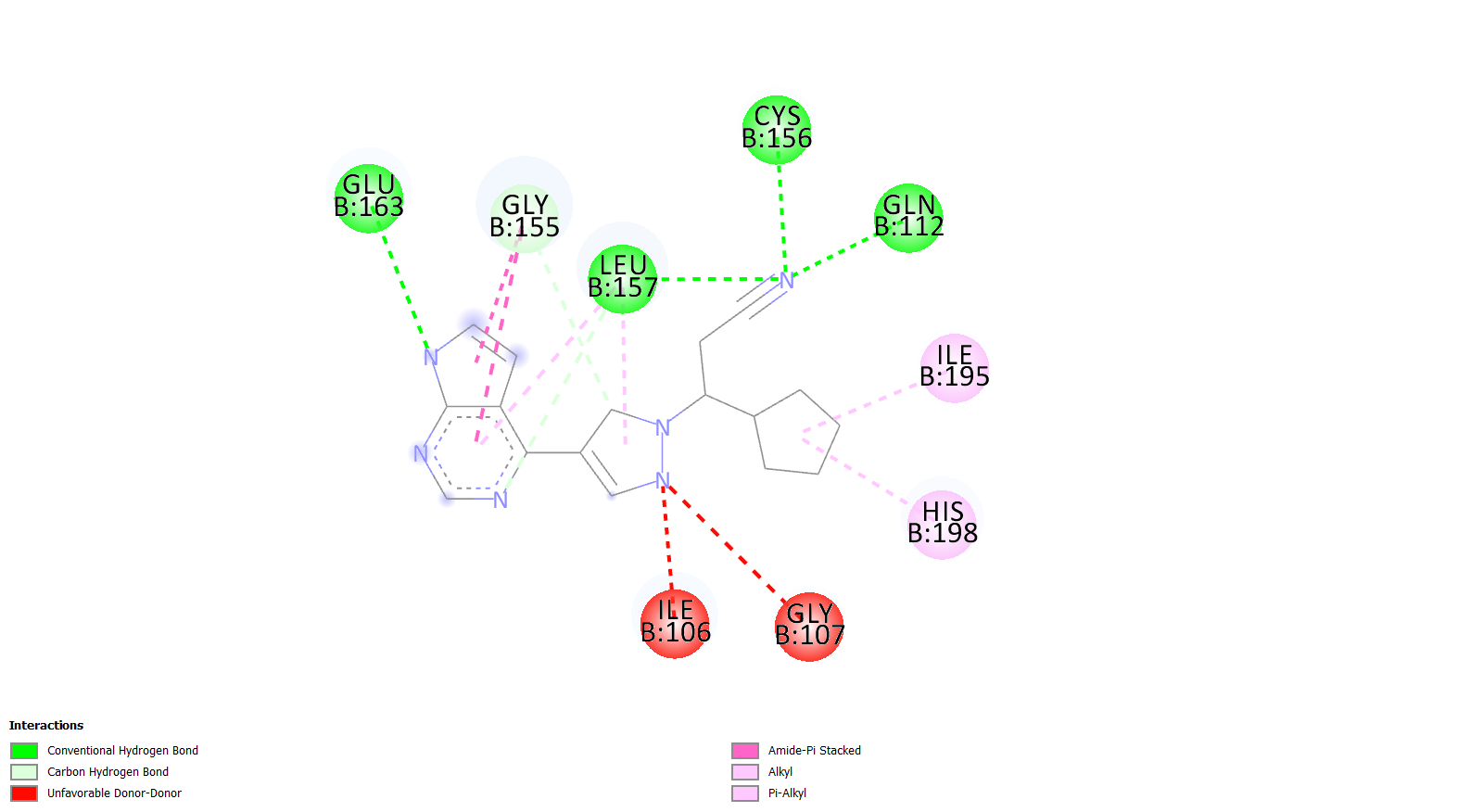


Figure S23 Ruxolitinib interactions in 1rl4. The image is rendered using Discovery Studio Visualizer 2017 R2.


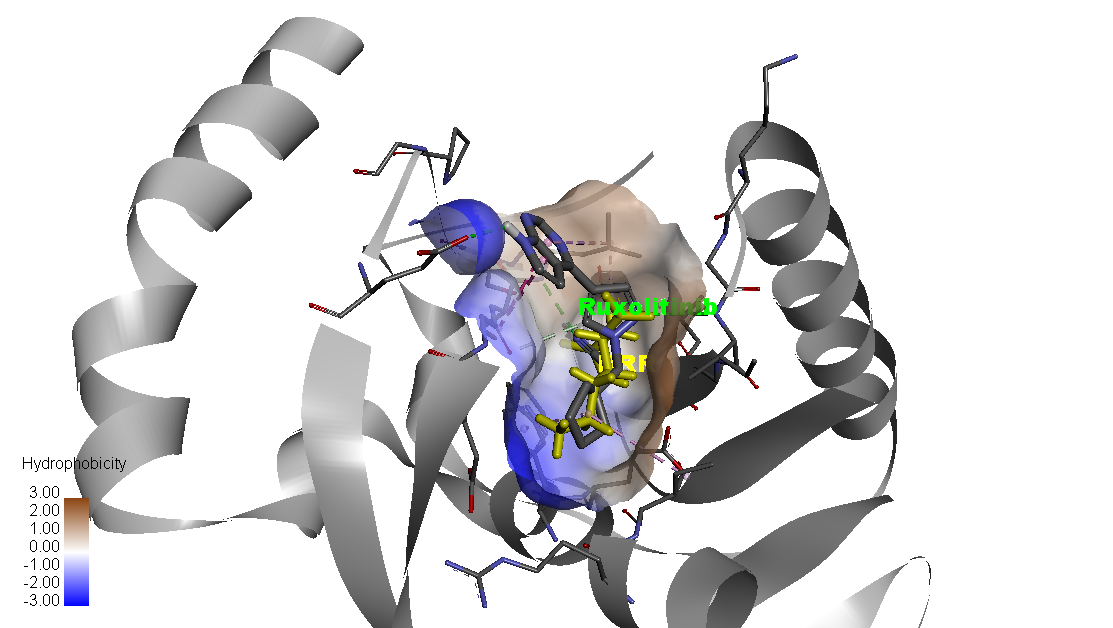


Figure S24 Ruxolitinib binding pose in 1rl4. The image is rendered using Discovery Studio Visualizer 2017 R2.

**1-deoxy-D-xylulose 5-phosphate reductoisomerase (PfDXR, PDB ID: 4gae)- Hydromorphone (DB00327):** 1-deoxy-D-xylulose 5-phosphate reductoisomerase is an attractive apicoplastic target without human homolog present in the non-mevalonate pathway. This latter produces isopentenyl pyrophosphate, critical metabolites for both liver-stage and blood-stage parasite development^33^. The hydrophilic nature of the substrate binding pocket made challenging DXR inhibitor design. Fosmidomycin-clindamycin and fosmidomycin-piperaquine are in Phase II clinical trials^34^. Hydromorphone is a derived morphine analgesic binding mainly in the cofactor site, showing similar binding mode to its nicotinamide moiety. This region of the pocket presents a more lipophilic character and the compound make hydrophobic interactions with ILE302 and MET360. Other interacting residues include ASP182, GLY299, HOH1552, ILE89, SER88 through polar contacts (Figure S29). NADP+ is also a cofactor for codeinone reductase which converts codeinone to codeine, a close analogue to hydromorphone^35^. Hydromorphone scaffold differs from current DXR inhibitors which are mainly fosmidomycin analogues mimicking the protein natural substrate and thus binding in the active site^36^. The compound can be extended toward the cofactor diphosphate binding region but more interestingly toward the substrate binding site, around the metal coordination sphere for a bisubstrate inhibitor.


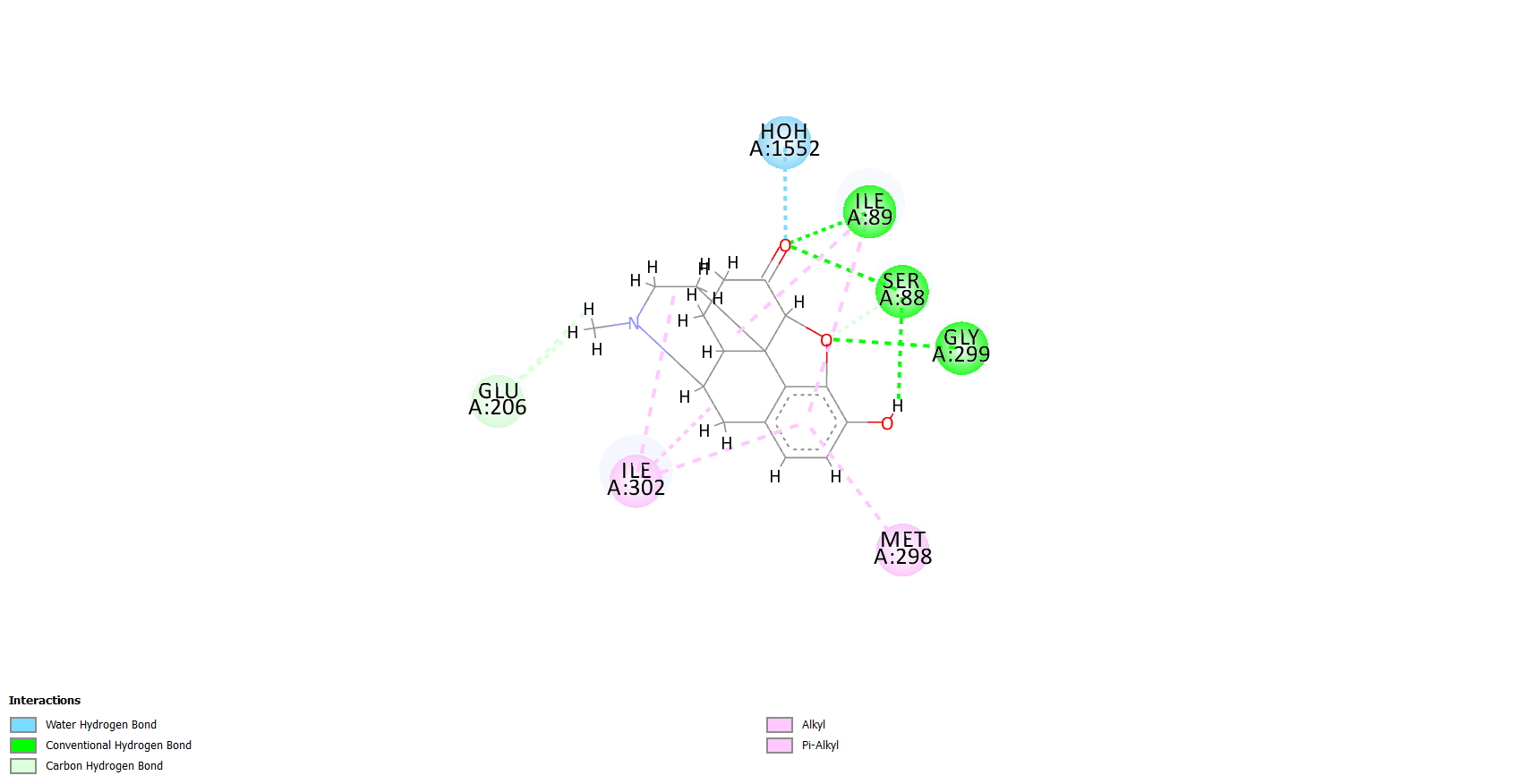


Figure S25 Hydromorphone interactions. The image is rendered using Discovery Studio Visualizer 2017 R2.

**Glutathione S-transferase (Pf-GST1, PDB ID: 1q4j) - Saxagliptin DB06335:** 1q4j is the structure of the glutathione S-transferase (Pf-GST1) in complex with an inhibitor: S-hexyl-glutathione (GTX). The enzyme plays a role in non-polymerized heme sequestration and may also serve as an in vivo buffer for the parasitotoxic ferriprotoporphyrin IX^37^. Chloroquine, primaquine and artemisinin showed weak inhibition effect on Pf-GST1^38,39^. The inhibitor in the structure (GTX) interacts with TYR9, PHE10, LYS15, GLN58, VAL59, GLN71, SER72, ASN111, PHE116, LYS117, THR121, TYR211, HOH216. Saxagliptin (rINN) is an orally active hypoglycemic (anti-diabetic drug). Its most energetically favourable pose (choosen in this case) showed an interesting pose as it fit deeper in a hydrophobic sub-pocket in the binding site and interacts with ASN111, GLN104, GLN73, GLY14, HIS107, LEU18, LYS15, PHE100, TYR108, VAL103 (Figure S27). The compound forms a hydrogen bond with LYS15, reported to play a role in the further stabilization of GSH or the electrophilic co-substrate as it is in close proximity to the S-hexyl-GSH sulfur. The residue might be promising for structure-based drug design^37^. However, three other residues were reported to be important for inhibitor recognition. The three invariant G-site ligands, TYR9, GLN71, ASP105 form polar contact with the substrate or inhibitor (GTX) and are conserved in Mu-class GSTs^37^. The pose is mostly favoured by the deep and enclosed hydrophobic subpocket where it hydroxytricyclo settles, making numerous hydrophobic contacts. However, despite the absence of interaction with key catalytic residues the pose may still prevent GHS from binding. An interesting optimization strategy would be the merging of GTX and saxagliptin (Figure S26), thus, extending this saxagliptin toward the GTX site, which could result in a hybrid compound with stronger potency.


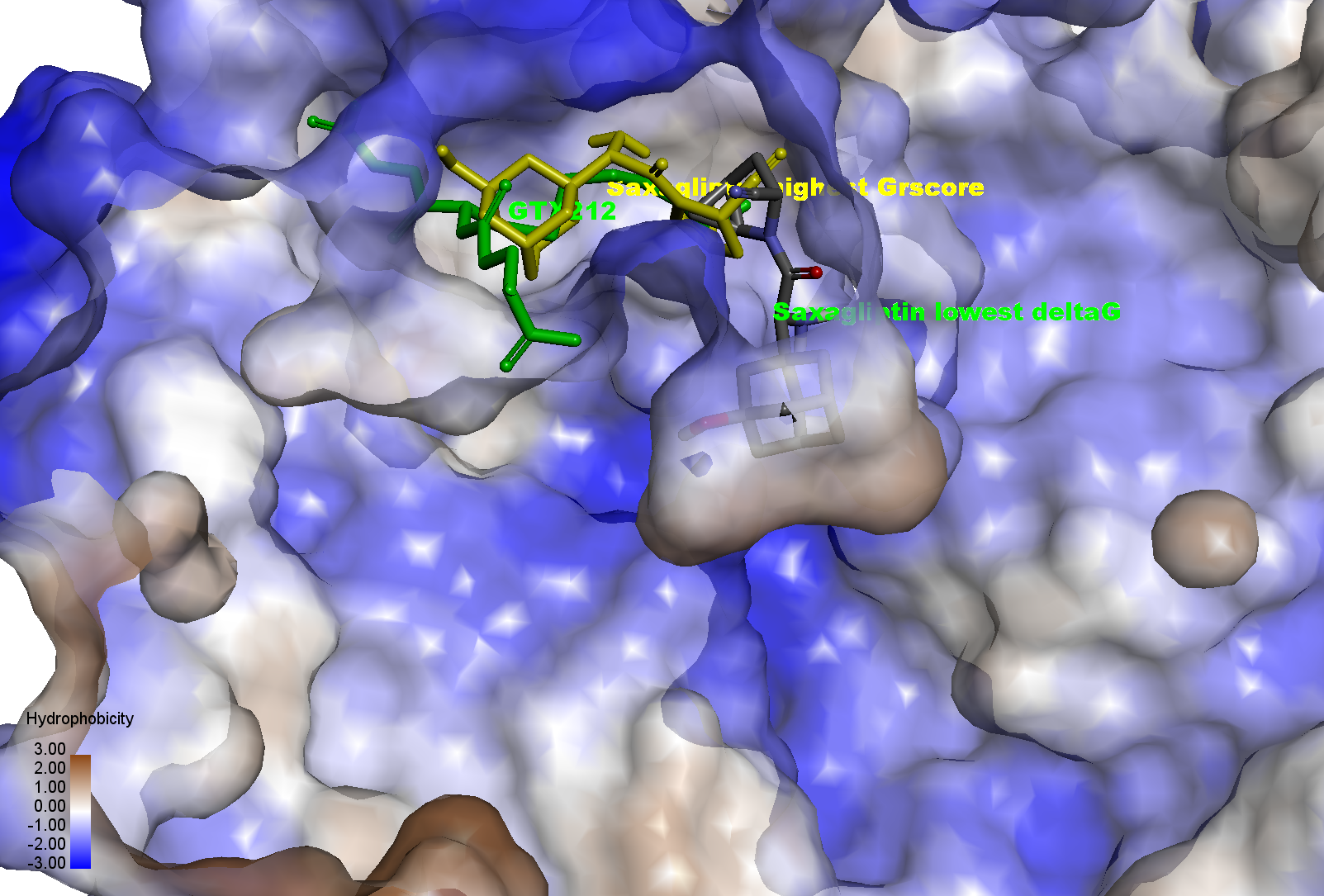


Figure S26 Saxagliptin and GTX binding poses. The image is rendered using Discovery Studio Visualizer 2017 R2.


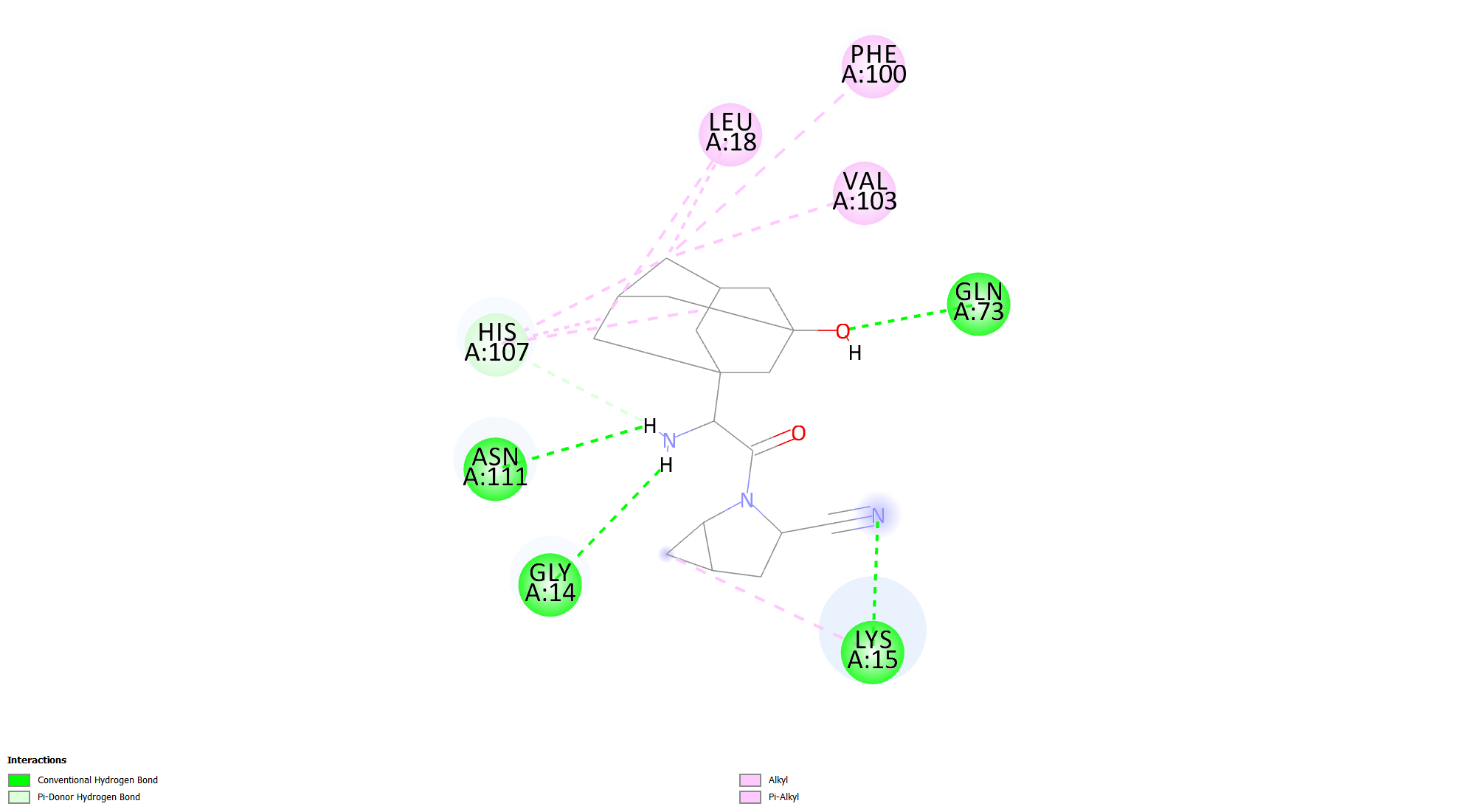


Figure S27 Saxagliptin interactions. The image is rendered using Discovery Studio Visualizer 2017 R2.

**D-aminoacyl-tRNA deacylase (DTD, PDB ID: 4nbj) - Triamcinolone (DB00620):** D-aminoacyl-tRNA deacylase (DTD) prevents D-amino acids to be incorporated into proteins. The structure is in complex with a substrate-mimicking analogue D-tyrosyl-3’-aminoadenosine^40^. Its role in protein translation might make it essential for the parasite liver stage. Triamcinolone is an agonist of glucocorticoid receptors indicated in the management of various disorders in which corticosteroids are indicated. The docked pose presents a molecular interactions pattern with efficient use of the polar groups. Four oxygens engage in hydrogen bonding with GLY138, ASN139, GLN88, PRO150, and the compound also makes a hydrophobic contact with PHE89 (Figure S29). This later residue and GLY138 play a role in the protein substrate accommodation. GLY138 but also SER87 side chain nitrogen and hydroxyl respectively and GLY149, GLN88 are also implied in substrate binding but were not playing a catalytic role^40^. A difference in the two binding modes (docked compound and co-crystallized ligand) is that D3Y adenine ring presents a deeper fit in the pocket while the docked pose binds on a more exposed area of the pocket (Figure S28). The adenosine-binding pocket is highly conserved and represents the protein functional site^40^. While not interacting with the depth of the pocket, Triamcinolone can be expected to competitively inhibit the natural substrate by its competitive pose. A development strategy could merge the adenine ring to the hydroxyl group to extend triamcinolone into the pocket. So far, DTD has not been an attractive target, few inhibitors are reported so far (no assay in Chembl for *P. falciparum*). D-amino acids used in combination with known inhibitors showed improved inhibitory activity^41^. Triamcinolone has previously reported activity against *P. falciparum* (ChemBL assay ID: 646859)^11^.


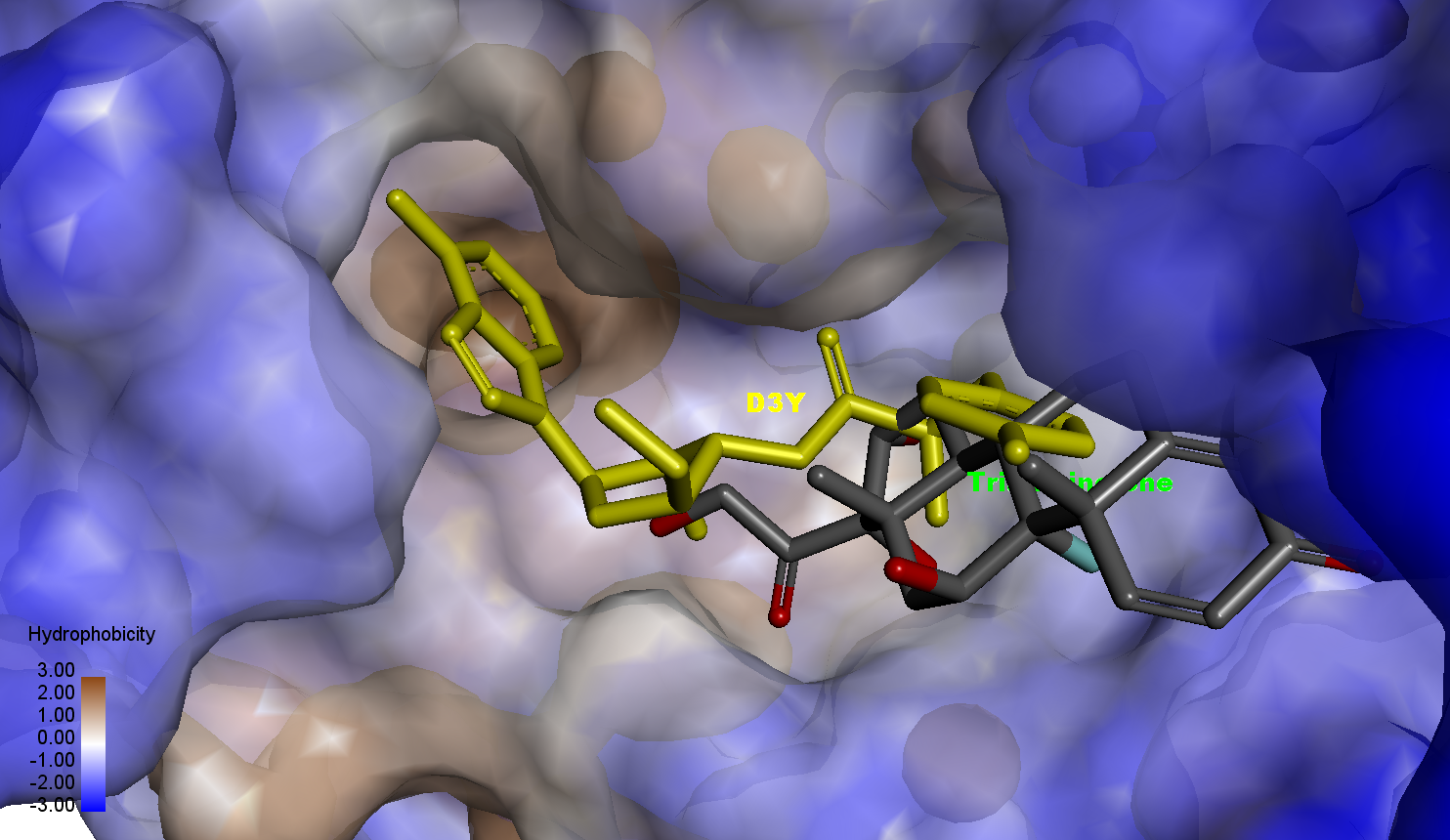


Figure S28 D3Y and Triamcinolone pose. The image is rendered using Discovery Studio Visualizer 2017 R2.


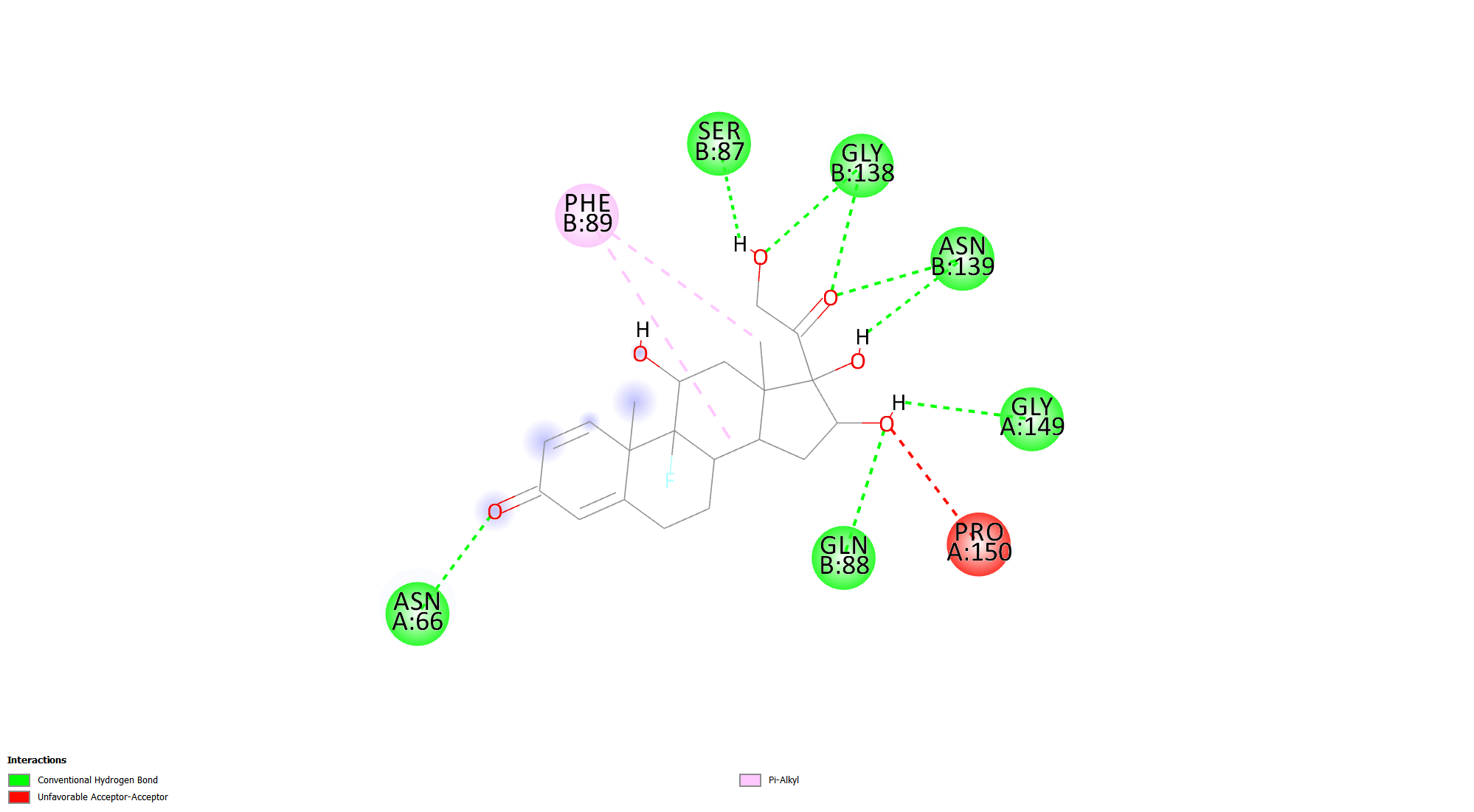


Figure S29 Triamcinolone interactions. The image is rendered using Discovery Studio Visualizer 2017 R2.

**Spermidine synthase (PfSpdSyn, PDB ID: 2pt6) - Sotalol (DB00489):** Spermidine synthase is an enzyme of the polyamine biosynthesis pathway here in complex with the substrate S-adenosylmethionine decarboxylase (dcAdoMet). The protein catalyses putrescine synthesis through the transfer of an aminopropyl moiety from decarboxylated S-adenosylmethionine (dcAdoMet) and finally the formation of spermidine^42^. Sotalol is a highly soluble compound with a sulfanilide moiety, adrenergic beta-antagonist that is used in the treatment of life-threatening arrhythmias. The ligand is completely enclosed in the pocket where its sulfanilide moiety make hydrogen bonds with SER198, GLN72 and the hydroxamate moiety interacts with ASP196, GLN93 while having a clash with TYR264. ASP196 has been suggested to play a role in the proper orientation of the substrate^42^. The compound also interacts with GLN229, LEU86, LEU88, , TYR102 and VAL152. Tough sotalol was inactive in different ChEMBL assays (CHEMBL3214945, CHEMBL3214813, CHEMBL3214842, CHEMBL3215001). The two compounds are different in their scaffold and sotalol does not show any overlap with the purine moiety on dcAdoMet. PfSpdSyn is one of the targets for the promiscuous artemisinin^43^.

**Uncharacterized protein (kinase) (PDB ID: 2pml) - Terazosin (DB01162):** 2pml is a kinase structure in complex with an ATP analogue inhibitor, Adenosine 5’-(beta,gamma-Imino)triphosphate. Terazosin is a quinazoline, selective alpha1-antagonist used for the treatment of symptoms of benign prostatic hyperplasia (BPH). It has a similar binding mode to Adenosine 5’-(beta,gamma-Imino)triphosphate interacting with ASN35, ASP123, ASP190, ILE42, LEU101, LEU179, LEU34, LYS55, SER189, TYR117. The interaction pattern is dominated by hydrophobic contacts with only one hydrogen bond with ARG32. The ATP analogue also interacts with similar residues in the structures. The two compounds are different in their binding modes with terazosin extending toward a more superficial are of the pocket while having a moiety fitting more deeply in the binding site. The ATP analog lies in the pocket, though not in the deepest part, crossing terazosin. Also, the two structures are significantly different (Tanimoto similarity 0.23) and terazosin does not show significant similarity with available inhibitors (Target ID: CHEMBL6169) the highest being 0.50 for CHEMBL602580. Terazosin could thus present a new scaffold for the enzyme inhibitors.

**Histo-aspartic protease (HAP) (PDB ID: 3qvi) - Stavudine (DB00649):** PfHAP is involved in the host hemoblin degradation process by the parasite. 3qvi represents the mature enzyme structure with a potent peptidomimetic inhibitor. Stavudine is a dideoxynucleoside analogue that inhibits reverse transcriptase and has in vitro activity against HIV. It binds in the inhibitor KNI-10395 binding site and interacting with LEU73, LEU138, ASN37, SER35, TRP39, HIS32, VAL120, PHE279 while clashing with SER36. ASP32 and HIS32 are catalytic residues ^44^. KNI-10395 also interacts with LEU73, PHE279, SER35, VAL120^44^. Stavudine is a small molecule compared to KNI-10395 (MW: 224) and only occupied a small region of the large hydrophobic pocket. KNI-10006 is the only reported in ChemBL (Target ID: CHEMBL6075). Stavudine may be a good starting point for fragment growing in the large pocket (Figure S 30) given its low MW(224) and high LipE (9).


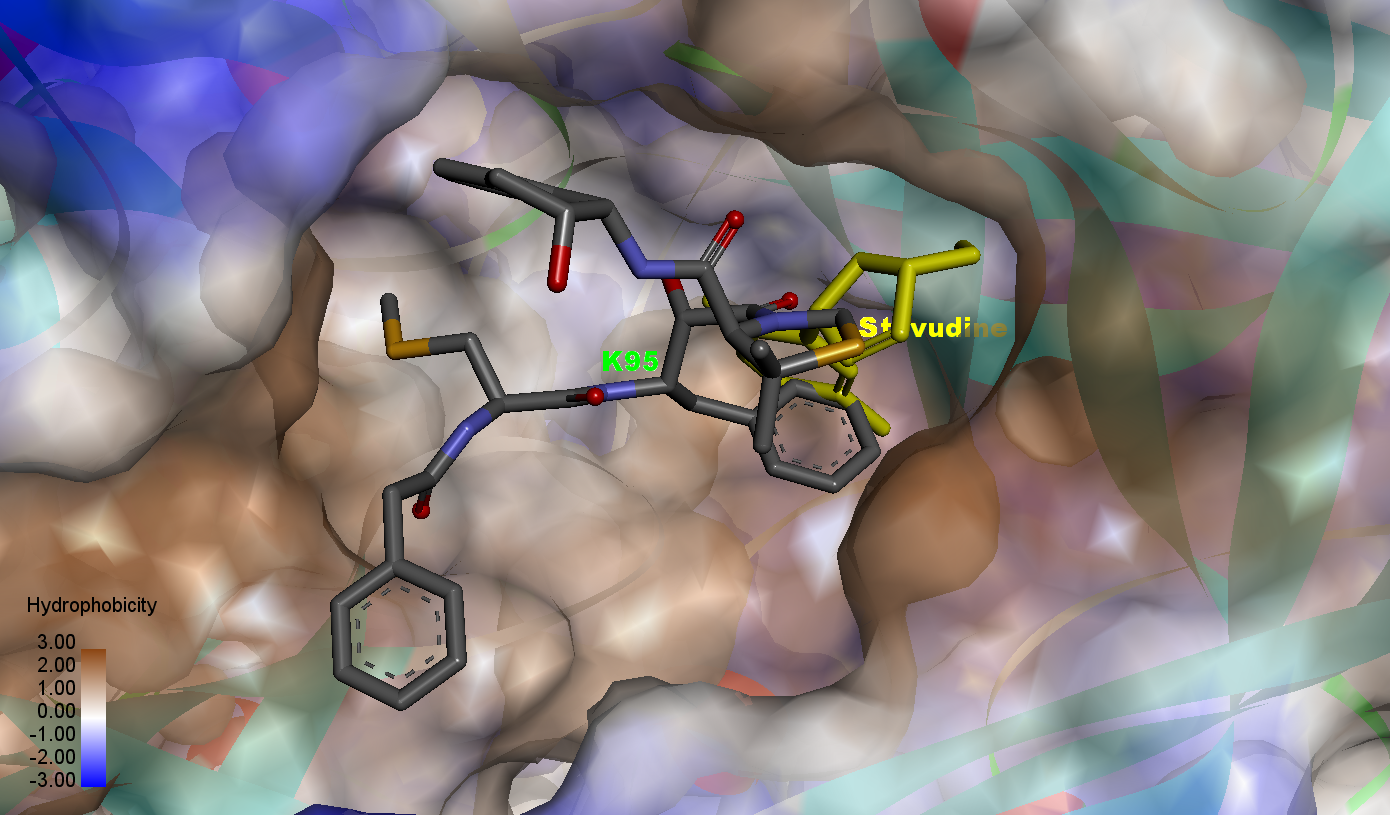


Figure S30 K95 and stavudine pose. The image is rendered using Discovery Studio Visualizer 2017 R2.


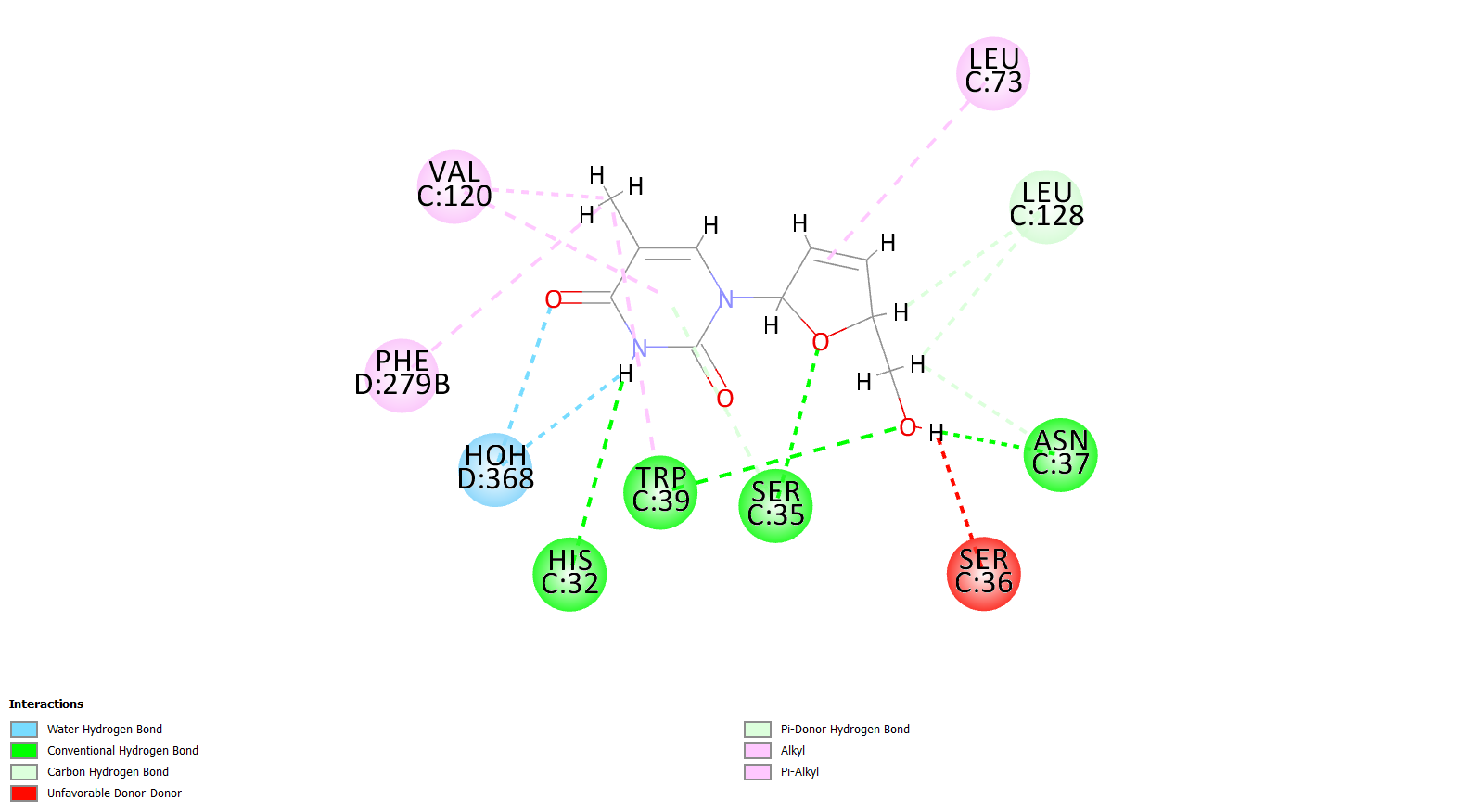


Figure S31 Stavudine interactions. The image is rendered using Discovery Studio Visualizer 2017 R2.

**Enoyl-acyl carrier reductase (PfENR, PDB ID:1ngh) - Moclobemide (DB01171):** PfENR is an essential enzyme in the final step of the fatty acid biosynthesis pathway. It converts trans-2-enoyl-ACP (acyl carrier protein) to acyl-ACP in an NADH-dependent manner^20,45^. In 1ngh, its structure is crystallized with ATP. The first predicted hit from these results for the protein was triclosan, a known Enoyl-[acyl carrier protein] reductase inhibitor for different bacteria with a reported nanomolar potency^46^. But triclosan is not annotated as targeting *P. falciparum* in DrugBank^47^, so it was present in out ligand set. Interestingly, the protein was also crystallized with triclosan, which explains the high Grscore (0.82) and a similar docked pose with a binding energy of -9.3 kcal/mol. This finding supports the ability of the screening pipeline for inhibitor identification. Because the first predicted compound for the target is a known inhibitor. The second hit for the enzyme was moclobemide (DB01171). The compound is known to be a reversible monoamine oxidase inhibitor (MAOI) used to treat major depressive disorder. It fits well in the binding site and presents a comparable binding mode to triclosan with similar interacting residues. Both compounds have two connected benzene rings with a chloride substituent on one of the rings. The chloride has many alkyl contacts with ILE369, PHE368, ALA372 and PRO314. It fits deep in the pocket in a very hydrophobic region (see Figure S5). Moclobemide also forms a hydrogen bond with ALA219 (absent in triclosan's binding mode), and one of its benzene rings has π-π interactions both with TYR267 and the cofactor nicotinamide adenine dinucleotide oxidized (NAD) (Figure S 32c). These π-π interactions are also observed with triclosan. While the nitrogen on the connecting chain in moclobemide has an unfavourable van der Waals interaction with TYR277, it is notable that the corresponding chain in triclosan is much shorter. Indeed, it only consists of an ether while in moclobemide it is a four-atom chain increasing the likelihood of clashes. In terms of the life cycle of *P. falciparum*, PfENR is a liver-stage essential enzyme and is not a target for any approved antimalarial. Further triclosan has been found to target both wild-type and pyrimethamine-resistant *P. falciparum* and *P. vivax* dihydrofolate reductases^48^. Moclobemide has previously been shown to show antimalarial activity^49,50^.

**Adenylosuccinate synthetase (PfAdSS, PDB ID:1p9b) - Tafamidis (DB11644)**: Adenylosuccinate synthetase (AdSS) catalyzes the synthesis of adenosine monophosphate (AMP) from inosine monophosphate (IMP) in the salvage pathway for purine nucleotide biosynthesis. Protozoa rely exclusively on the salvage pathway for purine nucleotide synthesis^51,52^. To date, the enzyme has very few inhibitors reported (ChEMBL, PubChem ^25^, BindingDB^53^, DrugBank^47^), hadacidin being the only reported one for PfAdSS^54^. Tafamidis is indicated for the treatment of transthyretin amyloidosis. Here, in the observed binding of tafamidis, the compound fully occupies a complete buried pocket where 6-O-phosphoryl inosine monophosphate (IMO) also binds and interacts with an MG ion. However, tafamidis also shows an unfavourable interaction with the metal ion. The binding pocket is polar, especially around the metal coordination region; however, tafamidis is hydrophobic (MW:306 Da; logP: 4.4; PSA: 63 Å^2^). It makes hydrophobic contacts with ILE138, LEU145, while also forming hydrogen bonds with HIS54, ASN232 and GLY53 on the carboxylic acid group (Figure S 32b). ASN232 is reported to be a catalytic residue for this target, while GLY53 coordinates the metal ion^52^. The IMO scaffold is characterized by two terminal phosphate groups which are significantly different from tafamidis. These groups form a network of hydrogen bonds with ASN51, THR247, THR141, LYS29, ASP26, GLY53, HIS54 ASN232, which is more extensive than that observed with tafamidis.

**L-lactate dehydrogenase (PfLDH, PDB ID: 1u5c) - gemifloxacin (DB01155):** 1u5c is the structure of *Plasmodium falciparum* lactate dehydrogenase (PfLDH), an essential protein in its lifecycle for energy generation. It catalyzes the conversion of (S)-lactate to pyruvate^55^. The structure 1u5c is complexed with 3,7-dihydroxynaphthalene-2-carboxylic acid (DHNCA) and NAD+. Gemifloxacin is an oral broad-spectrum quinolone antibacterial agent used in the treatment of acute bacterial exacerbation of chronic bronchitis and mild-to-moderate pneumonia. It was observed to bind to 1u5c in the large substrate-binding site. This latter does not show a pronounced hydrophobic character, neither does nor does gemifloxacin (cLogP: 0.966, PSA:123.04 Å^2^). Gemifloxacin diazanaphthalene moiety binds in the hydrophobic groove showing the same binding arrangement as the naphthalene moiety in DHNCA (see Figure S6). Naphthalene-based compounds are a core scaffold of the enzyme inhibitors^55^. Gemifloxacin is larger than DHNCA and contains a pyrrolidine ring that extends and was observed to interact with ASN140. It also interacted with ALA236, ARG171, ASN140, MET325, PRO141, THR101 among which ARG171 and ASN140 are important catalytic residues^55^. The cyclo-propyl ring of gemifloxacin was observed to make a hydrophobic contact with the cofactor NAD336 and two hydrogen bonds through its carboxylic acid group with ARG171 (Figure S 32a). The carboxylic acid functionality in 3,7-dihydroxynaphthalene-2-carboxylic acid (DHNCA) also forms a hydrogen bond with ARG171. It is noteworthy that gemifloxacin also has an alternate binding mode in which the acid group is a reverse orientation. The binding pocket is large and the gemifloxacin could be extended as optimization strategy in two possible sub pockets, toward THR322 or ASP168.

**Protein serine/threonine kinase-1 (PDB ID: 3llt) - Sitaxentan (DB06268):** 3llt is the structure of a protein serine/threonine kinase-1 in complex with an adenosine triphosphate (ATP) analog, phosphoaminophosphonic acid-adenylate ester (ANP). Sitaxentan is an endothelin receptor antagonist used for pulmonary arterial hypertension. ANP binds in its binding site, a trench-like pocket (see Figure S4) and interacts with THR562, GLY564, LYS581, GLU596, ASP720, VAL566, ILE719, PHE563, PHE630, ALA593, VAL566 and VAL583. Sitaxentan was observed to binds the same site, interacting with similar residues, making polar contacts through its aminosulfonyl group to GLY564, PHE563, LYS581, THR562 and ASP720, in doing so setting up 5 hydrogen bond interactions (Figure S 32d). This compound also makes hydrophobic contacts with VAL583, ALA593, ILE719, VAL566 and PHE630. Sitaxentan has a benzodioxole group which shows a similar binding mode and interactions to the ANP purine ring although ANP forms more hydrogen bonds. This benzodioxole moiety engages in a π-σ interaction with VAL566 and a π- π T-shaped interaction with PHE630. In contrast to the phosphate groups on ANP, sitaxentan has aminosulfonyl and thiophene groups. Further, sitaxentan has an isoxazole ring which extends in a further cavity in the pocket interacting with ALA593, and VAL583. Hence, it presents a significantly different scaffold from ANP. This serine/threonine kinase plays a role in both mosquito and liver stages of parasite growth in the rodent malaria parasite, *P. berghei* ANKA (PbA)^56^. To date, this target has no reported inhibitor in ChEMBL^6^.


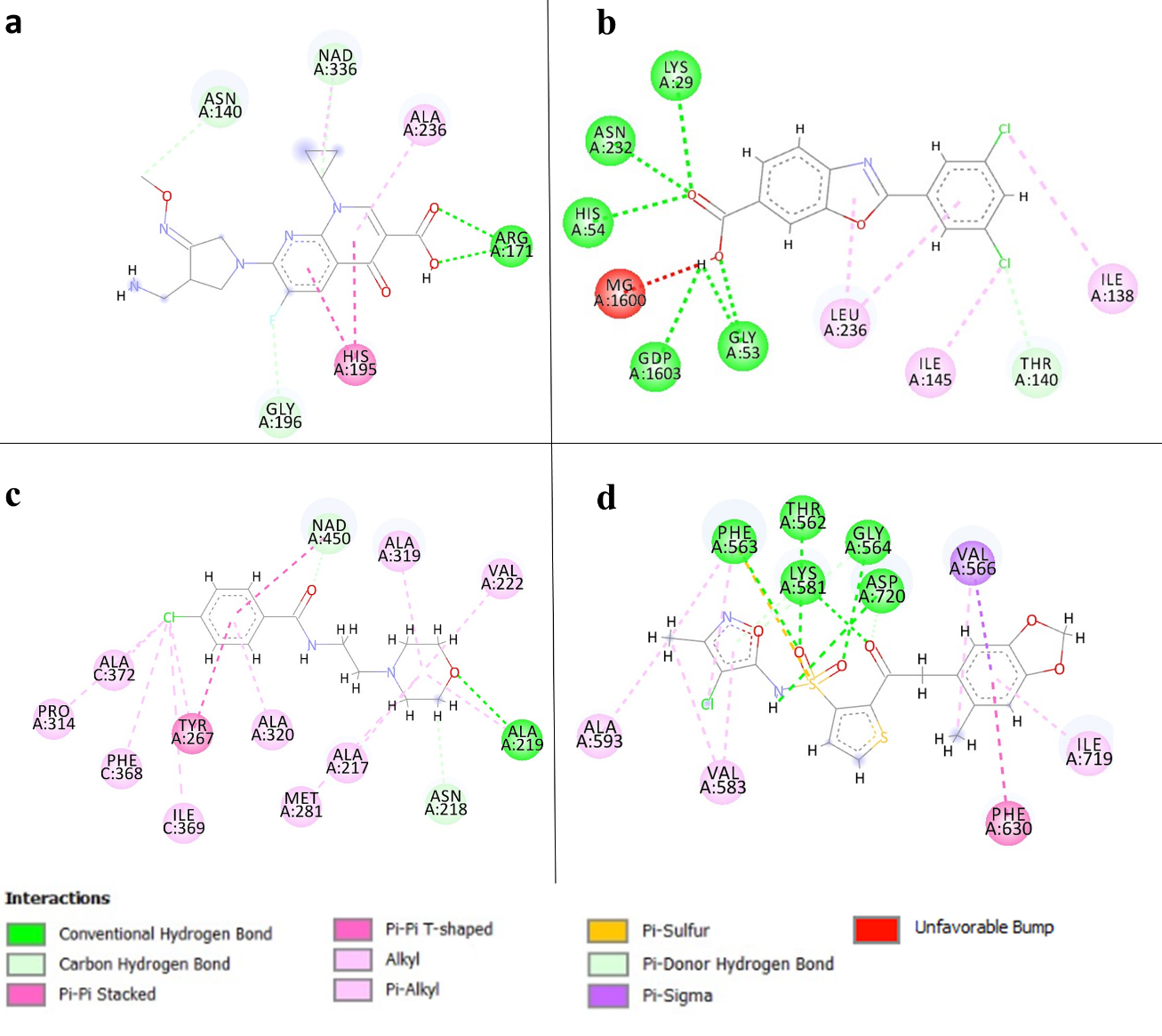


Figure S32 2D plot of intermolecular interactions depicted using Discovery Studio Visualizer 2017 R2. a) gemifloxacin and PfLDH b) Tafamidis and PfAdSS c) moclobemide and PfENR d) Sitaxentan and PfENR. Dashed lines represent the different interactions and their color the interaction type. Colored circles represent residues with their the tree letter code, chain identifier and residue number.

## Z-test mean apo Rg vs mean complex Rg

Table 1 Z-test of the differences of Rg (apo - complexes)

| **Complexes** | **tstat** | **pvalue** | **mean_comp** | **mean_apo** |
| --- | --- | --- | --- | --- |
| **3fi8_DB00469** | -4.91 | 0.0 | 0.23 | 0.24 |
| **2pmn_DB00555** | -44.06 | 0.0 | 0.19 | 0.24 |
| **2igx_DB08868** | 18.67 | 0.0 | 0.18 | 0.16 |
| **4gae_DB00327** | 22.63 | 0.0 | 0.21 | 0.18 |
| **4j56_DB00457** | -17.32 | 0.0 | 0.17 | 0.19 |
| **1v0o_DB01217** | 37.64 | 0.0 | 0.15 | 0.12 |
| **2pml_DB01162** | 5.6 | 0.0 | 0.31 | 0.3 |
| **4py6_DB01291** | 25.77 | 0.0 | 0.26 | 0.22 |
| **1u5c_DB01155** | -47.79 | 0.0 | 0.14 | 0.18 |
| **2yoh_DB01001** | 46.17 | 0.0 | 0.5 | 0.39 |
| **2pt6_DB00489** | 29.54 | 0.0 | 0.19 | 0.15 |
| **3o8a_DB01203** | -5.5 | 0.0 | 0.14 | 0.14 |
| **3jqr_DB00365** | 11.21 | 0.0 | 0.27 | 0.25 |
| **3uj8_DB01048** | -19.16 | 0.0 | 0.12 | 0.14 |
| **3qvi_DB00649** | 6.2 | 0.0 | 0.16 | 0.16 |
| **1d5c_DB00883** | 23.16 | 0.0 | 0.16 | 0.15 |
| **4nbj_DB00620** | 14.68 | 0.0 | 0.15 | 0.13 |
| **3llt_DB06268** | 34.95 | 0.0 | 0.22 | 0.17 |
| **1nhg_DB01171** | -34.72 | 0.0 | 0.18 | 0.21 |
| **4mvf_DB05812** | 0.45 | 0.66 | 0.26 | 0.26 |
| **1rl4_DB08877** | 26.84 | 0.0 | 0.21 | 0.17 |
| **1q4j_DB06335** | 11.9 | 0.0 | 0.22 | 0.2 |
| **2bsx_DB00853** | 13.17 | 0.0 | 0.2 | 0.19 |
| **3fnu_DB00496** | 3.04 | 0.0 | 0.16 | 0.16 |
| **1p9b_DB11644** | 24.62 | 0.0 | 0.13 | 0.12 |

Differences between means were evaluated with double-sided Z-test. The p-value of the t-test was used to assess the difference in the mean values. Differences were considered statistically significant at P<0.05.

## Scoring schemes assessment through the Mean Ranking Error (MRE)

In an all-vs-all 1296 (36X36) docking experiments, all co-crystallized ligands were docked in the proteins giving a matrix of scores S[i,j] (i being the ligands and j the proteins). S[i,j] refers to the score of ligand i docked in protein j. QuickVina-W^57^, RF-Score^58^, GRIM ^59^, ligand efficiency metrics and the effect of standardization and complex ranking scheme were assessed using the Mean Ranking Error (MRE), previously described and used by Vigers et al ^60^ in a similar experiment. Given a matrix with the ligands on columns and proteins in rows, a ranking error (Errj) is calculated for each row (series i of ligands on protein j) using eq. 1. Sjj is the score for the co-crystallized ligand j on its respective protein j, Sjbest and Sjworst are respectively the scores for the top and the worst ligands on protein j. (Sjbest − Sjworst) is the range of scores on protein j. Sjbest − Sjj is the score difference between the best ligand and the co-crystallized ligand. This should be zero in a correct scoring. The mean of all Errj (across all proteins) gives the MRE. A perfect scoring will yield an MRE of zero while a random one will approximatively give 0.5 and one is the worst case.

$$Errj=\frac{S_{jbest}-S_{jj}}{S_{jbest}-S_{jworst})} (1)$$

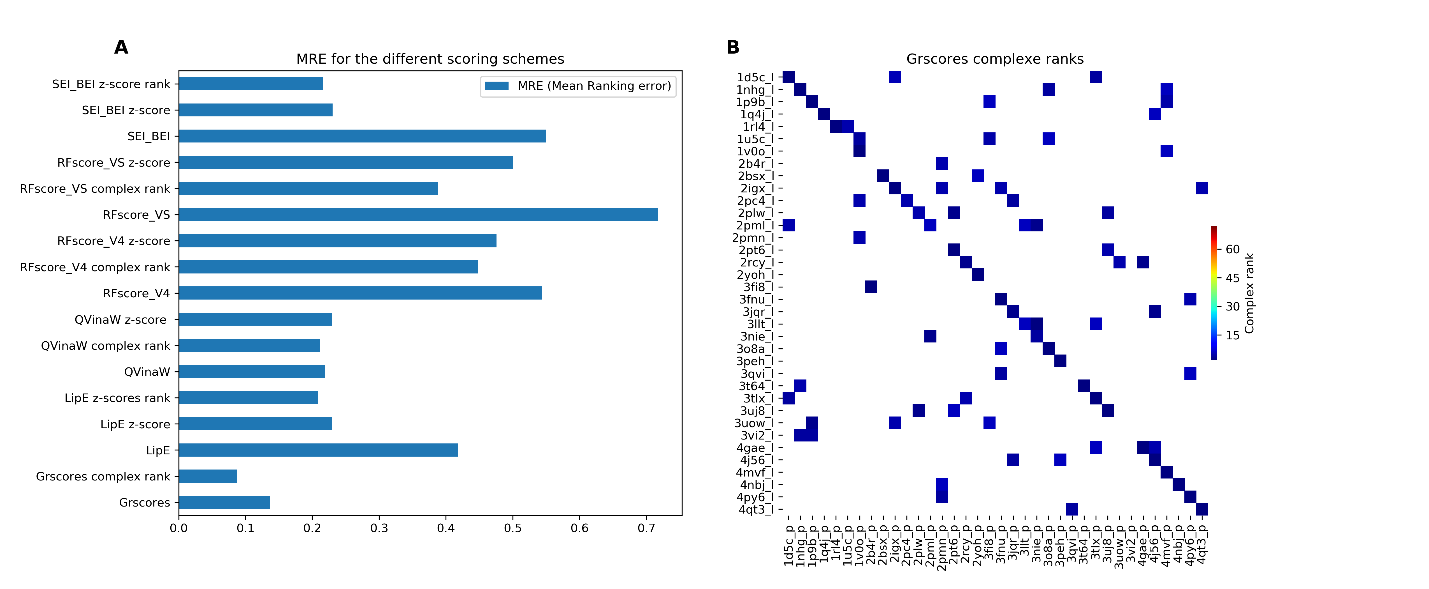


Figure S33 MRE values and Complexes ranks from Grscores. A) Bar chart of the MRE values for the different scoring schemes. B). Heatmap of the Grscores complex ranks described in the methods section (only complexes with a rank value ≤ 6 are shown for clarity. On the heatmaps, rows (ligands) and columns (proteins) are alphabetically ordered.

SEI_BEI correspond to the the radial coordinate ($\sqrt{SEI^{2}+BEI^{2}}$) as described in the methodology section. SEI_BEI z-score corresponds to the standardized value of SEI_BEI. SEI_BEI z-score rank is obtained after applying the complex ranking scheme applied on the SEI_BEI z-score. The same approach is applied to the other scoring scheme (RFscore_VS, RFscore_V4, QVinaW, and LipE). Grscores are not standardized as they are similarity values.

Except QvinaW, MRE improvement through scores’ standardization and complexing ranking is consistent in the different scoring schemes (Figure S 33 A). For example one can note a decrease in the MRE from SEI_BEI to SEI_BEI z-score and finally to SEI_BEI z-score rank. Also a similar trend is observed in RFscoreVS and RFscore_V4 and LipE. Complex ranking provided the lowest MREs while the highest values are observed for the scores before standardization and complex ranking (Figure S 33 A). Surprisingly, the machine learning scoring function RFscore yielded the highest MRE values. Indeed, both RF-score-vs 1.0 and RF-Score 4 were found to poorly rank the co-crystallized ligands with an MRE of 0.71 and 0.544 respectively while QuickVina-W showed a better MRE of 0.21. Both versions, RF-score VS and V4 had an MRE greater than 0.5, worse than random, therefore, they were not included in the subsequent experiments.

Lipophilic efficiency and the radial coordinates in the SEI, BEI 2D efficiency plane had MRE of 0.41 and 0.54 respectively, reduced to 0.2 and 0.21 respectively after standardization and complexes ranking. This MRE value is comparable to the QuickVina-W one. The ligand efficiency indices include the energy term which could explain their similar MREs. Grscore after complex ranking gave the best MRE of 0.08.

Some co-crystalized ligands had a Grscore lower than 0.594 when docked in their respective proteins. This value is the threshold for classification similar/dissimilar to co-crystallized ligand interaction pattern ^59^. Indeed, ligands in the following structures: 3qvi, 3uow, 3vi2, 1u5c showed a Grscore of 0.57, 0.56, 0.57 and 0.58 respectively. This is explained by the absence of good binding poses in the first place with high RMSD underlying the dependency of the Grscore on a good predicted pose. Interestingly, ligands in 2pc4, 2rcy, 3jqr had RMSD above 2 Å, 5, 2.1, 5 Å respectively. Still, they have low complex ranks, 5, 3, and 3 respectively (Figure S 33 B, for clarity only complexes having a rank <= 6 are shown). It is noteworthy that their RMSD is low enough to expect them binding in the reference compound binding sites. On the other hand, 3fi8 despite having a good ligand pose, had a complex rank of 16, a protein rank of 11 and a ligand rank of 5. The protein rank is the main contributor to the poor complex rank considering that this is a pair of protein and its co-crystalized ligand. The protein 3fi8 had the highest average Grscore, thus showing the highest degree of promiscuity for the different ligands. The co-crystallized ligand has simple a simple interaction pattern consisting of hydrogen bonds with GLN290, ASP288 and a conserved water molecule in the binding site (HOH456). This interaction pattern may be easily reproduced by other compounds leading the protein to have high Grscores with different ligands. This suggest that the Grscore may be dependent on the complexity of the interaction pattern of the reference. Simple interaction patterns may easily be reproduced by other ligands, leading to promiscuity of protein having such simplified patterns.

Two false positives in the set, have a complex rank of 2 while not the original protein-ligand pairs: ligand in 3fi8 (OPE603) with the protein 2b4r, and the ligand in 3llt (ANP) with the protein 3nie (see fig. 2 B). In the first case, OPE603 (co-crystallized ligand in 3fi8), despite showing a high Grscore (0.7437), does not bind in the site of the reference ligand in 2b4r (AES602 in Chain O), but binds in another binding site and interact with AES602 (in chain P). The high Grscore can be explained by those interactions as it interacts with the reference ligand in 2b4r (see fig. 4). In the second case, the two proteins (3llt and 3nie) are two kinases co-crystallized with the same ligand: phosphoaminophosphonic acid-adenylate ester (HET CODE: ANP). Indeed ANP co-crystalized with 3llt binds in 3nie reproducing a very similar pose to the ANP in 3nie (see fig. 3). This can explain their complex rank of 2.


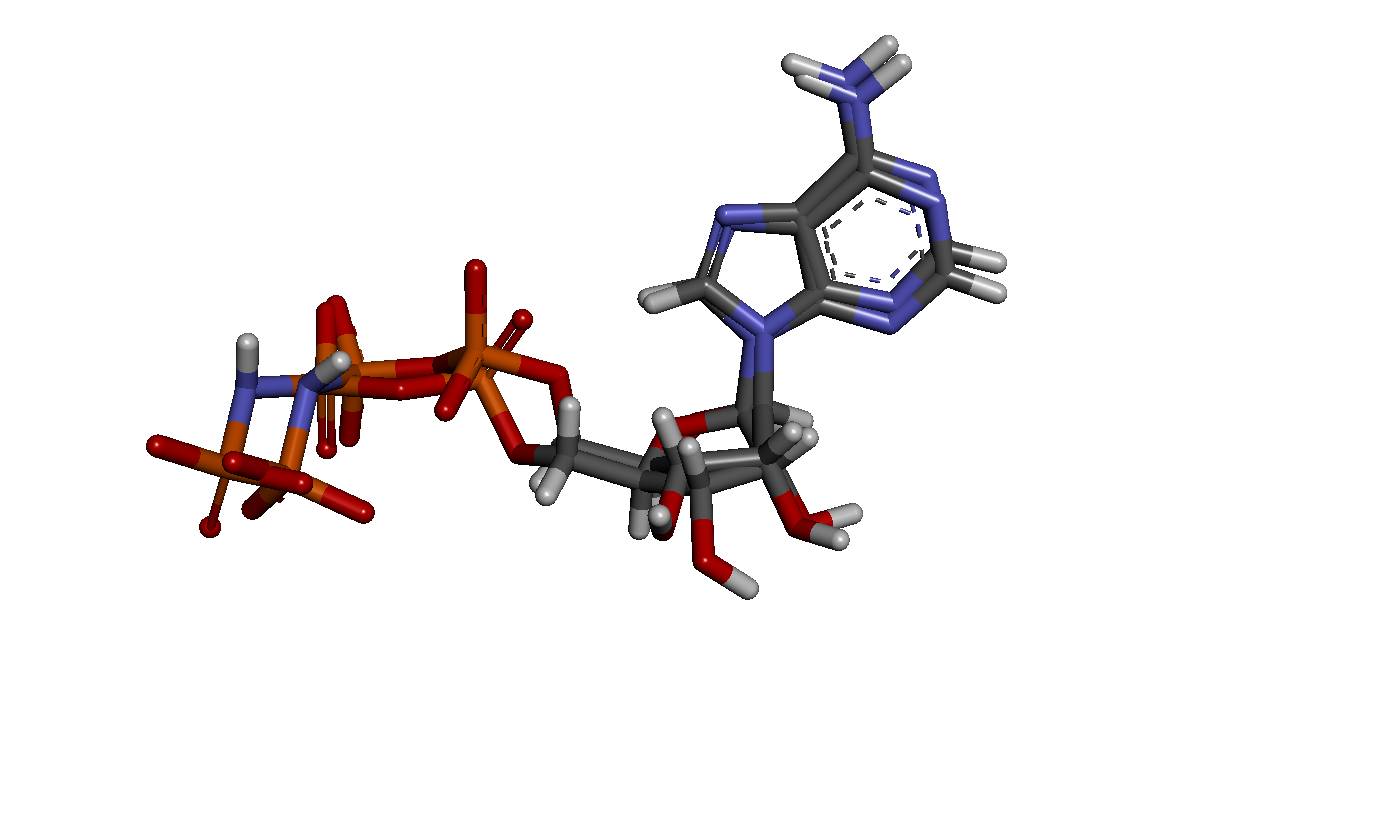


Figure S34: ANP in 3fi8 and 3llt. The image is rendered using Discovery Studio Visualizer 2017 R2.


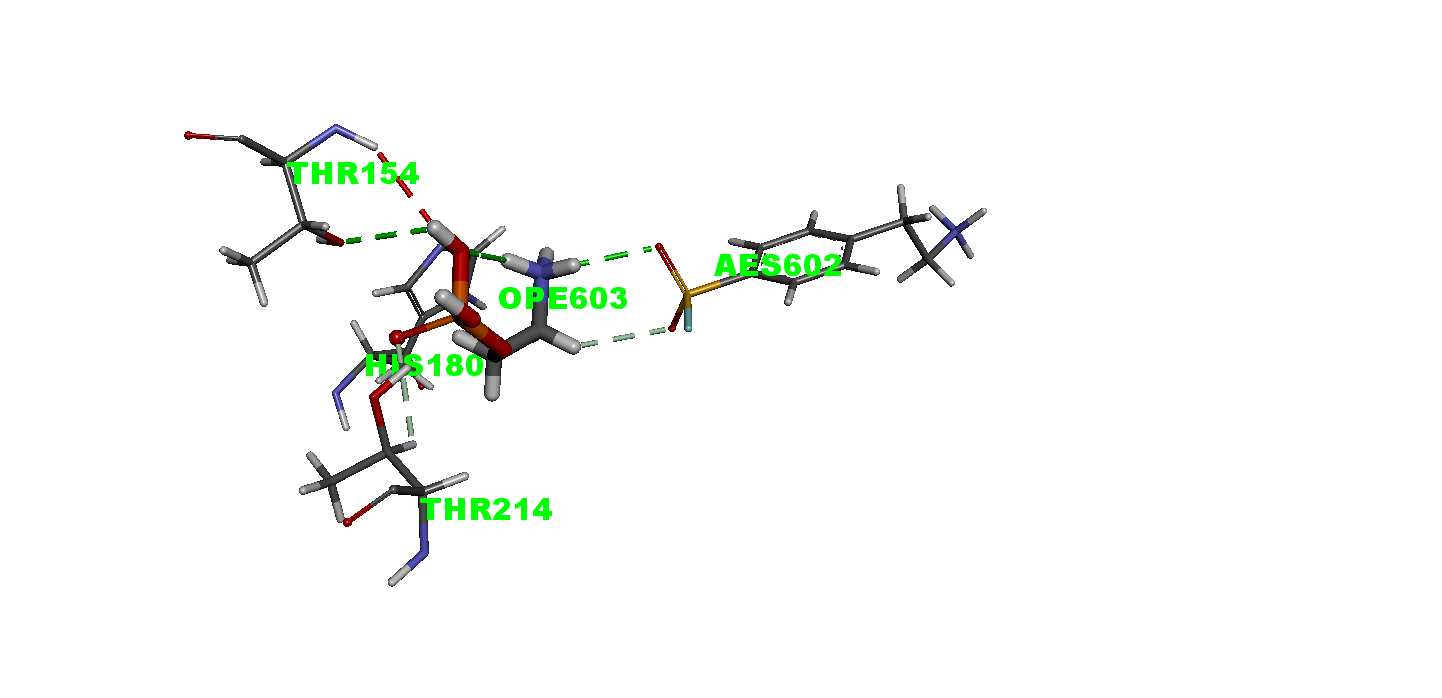


Figure S35: interactions OPE603-AES602. The image is rendered using Discovery Studio Visualizer 2017 R2.

## Top predicted complexes

Table 2. Top predicted complexes with targets PDB IDs and names, and compounds names, binding energies, GRIM Grscores, and ligand efficiency (LipE, SEI, BEI) values.

| PDB ID | Protein name | DrugBank ID | Compound name | Binding Energy (Kcal/mol) | Grscore | LipE | BEI | SEI | Mean PLIE^1^ (Kcal/mol) |
| --- | --- | --- | --- | --- | --- | --- | --- | --- | --- |
| 1nhg | Enoyl-acyl carrier reductase | DB01171 | Moclobemide | -9.0 | 0.73 | 8.2 | 36 | 23 | -146.70 |
| 1d5c | GTPase (Rab6) | DB00883 | Dianhydrosorbitol 2,5-dinitrate | -7.7 | 0.73 | 9.7 | 37 | 7 | -171.46 |
| 1p9b | Adenylosuccinate synthetase | DB11644 | Tafamidis | -10.3 | 0.71 | 6.1 | 34 | 17 | -151.97 |
| 1q4j | Glutathione S-transferase | DB06335 | Saxagliptin | -9.4 | 0.63 | 8.8 | 31 | 11 | -187.68 |
| 1rl4 | Peptide deformylase | DB08877 | Ruxolitinib | -9.0 | 0.69 | 6.2 | 31 | 12 | -150.06 |
| 1u5c | L-lactate dehydrogenase | DB01155 | Gemifloxacin | -8.2 | 0.67 | 8.1 | 23 | 7 | -213.04 |
| 1v0o | Cell division control protein 2 homolog | DB01217 | Anastrozole | -10.1 | 0.70 | 7.5 | 36 | 13 | -163.73 |
| 2bsx | Purine nucleoside phosphorylase | DB00853 | Temozolomide | -7.5 | 0.74 | 10.6 | 44 | 8 | -175.49 |
| 2igx | Plasmepsin 2 | DB08868 | Fingolimod | -7.9 | 0.79 | 5.6 | 29 | 13 | -177.18 |
| 2pml | Uncharacterized protein | DB01162 | Terazosin | -9.0 | 0.62 | 8.6 | 25 | 9 | -190.83 |
| 2yoh | Thymidylate kinase | DB01001 | Salbutamol | -7.7 | 0.70 | 7.4 | 36 | 12 | -151.25 |
| 3fi8 | Choline kinase | DB00469 | Tenoxicam | -8.8 | 0.74 | 7.8 | 28 | 10 | -173.48 |
| 3fnu | Plasmepsin III | DB00496 | Darifenacin | -9.5 | 0.73 | 6.0 | 23 | 18 | -152.76 |
| 3jqr | Ferredoxin–NADP reductase apicoplast | DB00365 | Grepafloxacin | -9.5 | 0.71 | 7.7 | 28 | 13 | -165.17 |
| 3o8a | Dihydroorotate dehydrogenase | DB01203 | Nadolol | -9.0 | 0.78 | 9.0 | 31 | 12 | -214.64 |
| 3qvi | Histo-aspartic protease | DB00649 | Stavudin | -7.6 | 0.60 | 9.3 | 38 | 10 | -142.12 |
| 3llt | Protein serine/threonine kinase-1 | DB06268 | Sitaxentan | -10.5 | 0.59 | 6.8 | 24 | 10 | -231.08 |
| 3uj8 | Phosphoethanolamine N-methyltransferase | DB01048 | Abacavir | -9.2 | 0.75 | 8.7 | 34 | 10 | -261.05 |
| 4gae | 1-deoxy-D-xylulose 5-phosphate reductoisomerase | DB00327 | Dihydromorfinon | -9.6 | 0.67 | 8.4 | 35 | 20 | -140.28 |
| 4j56 | Thioredoxin reductase 2 | DB00457 | Prazosin | -11.4 | 0.71 | 9.6 | 30 | 11 | -331.42 |
| 4mvf | Calcium-dependent protein kinase 2 | DB05812 | Abiraterone | -11.0 | 0.69 | 5.7 | 32 | 33 | -166.17 |
| 4nbj | D-aminoacyl-tRNA deacylase | DB00620 | Triamcinolone | -9.4 | 0.63 | 9.3 | 25 | 9 | -205.09 |
| 4py6 | Bromodomain protein putative | DB01291 | Pirbuterol | -6.7 | 0.69 | 7.2 | 33 | 9 | -190.13 |
| 2pmn | Protein kinase 7 | DB00555 | Lamotrigine | -7.8 | 0.69 | 6.7 | 34 | 10 | -201.50 |
| 2pt6 | Spermidine synthase | DB00489 | Sotalol | -7.9 | 0.62 | 7.7 | 32 | 11 | -224.17 |

1: PLIE: Protein-ligand interaction energy.

## Targets information

Protein cellular locations were determined using gene ontology annotations of the protein sequences.

Table 3 Targets information

| **PDB ID** | **Resolution** | **Ligand HET_CODE** | **Ligand name** | **Target class** | **pH** ^61^ | **Locations** |
| --- | --- | --- | --- | --- | --- | --- |
| **1d5c** | 2.3 | GDP | Guanosine-5'-diphosphate | Endocytosis/exocytosis | 6 | Golgi apparatus |
| **1nhg** | 2.43 | TCL | Triclosan | Oxidoreductase | 7.1 ^62^ | Apicoplast |
| **1p9b** | 2 | IMO | 6-o-phosphoryl inosine monophosphate | Ligase | 7.2 | Cytoplasm [GO:0005737] |
| **1q4j** | 2.2 | GTX | S-hexylglutathione | Transferase | 7.2 | Cytosolic |
| **1rl4** | 2.18 | BRR | (2r)-2-{[formyl(hydroxy)amino]methyl}hexanoic acid | Hydrolase | 7.1 ^62^ | Apicoplast [GO:0020011]; integral component of membrane [GO:0016021] |
| **1u5c** | 2.65 | BIK | 3,7-dihydroxy-2-naphthoic acid | Oxidoreductase | 7.2 | Cytoplasm |
| **1v0o** | 1.9 | INR | 2',3-dioxo-1,1',2',3-tetrahydro-2,3'-biindole-5'-sulfonic acid | Transferase | 7.2 | Cytoplasm [GO:0005737] |
| **2b4r** | 2.25 | AES | 4-(2-aminoethyl)benzenesulfonyl fluoride | Oxidoreductase | 7.2 | Cytoplasm |
| **2bsx** | 2 | NOS | Inosine | Transferase | 7.2 | Cytosol |
| **2foi** | 2.5 | JPA | 5-pentyl-n-{[4'-(piperidin-1-ylcarbonyl)biphenyl-4-yl]methyl}-n-[1-(pyridin-2-ylmethyl)piperidin-4-yl]pyridine-2-carboxamide | Oxidoreductase | 7.1 | Apicoplast [GO:0020011] |
| **2igx** | 1.7 | A1T | Asp_trp_asn | Hydrolase |  | Integral component of membrane [GO:0016021] |
| **2plw** | 1.7 | SAM | S-adenosylmethionine | Transferase | 7.1 ^62^ | Apicoplast (probable) |
| **2pml** | 2.6 | ANP | Phosphoaminophosphonic acid-adenylate ester | Transferase | 7.2 | Cytoplasm |
| **2pmn** | 2.8 | K51 | 4-(6-{[(1s)-1-(hydroxymethyl)-2-methylpropyl]amino}imidazo[1,2-b]pyridazin-3-yl)benzonitrile | Transferase | 7.2 | Cytoplasm [GO:0005737]; nucleus [GO:0005634] |
| **2pt6** | 2 | S4M | 5'-[(s)-(3-aminopropyl)(methyl)-lambda~4~-sulfanyl]-5'-deoxyadenosine | Transferase | 7.2 | Cytosol [GO:0005829] |
| **2rcy** | 2.3 | NAP | Nadp nicotinamide-adenine-dinucleotide phosphate | Oxidoreductase | 7.2 | Cytoplasm (soybean) |
| **2yoh** | 1.6 | WMJ | 1-[[(2R,3S,5S)-5-[5-methyl-2,4-bis(oxidanylidene)pyrimidin-1-yl]-3-oxidanyl-oxolan-2-yl]methyl]-3-(4-nitrophenyl)urea | Transferase | 7.2; 8 | Cytoplasm [GO:0005737]; cytosol [GO:0005829]; mitochondrion [GO:0005739]; nucleus [GO:0005634] |
| **3fi8** | 2.3 | OPE | Phosphoric acid mono-(2-amino-ethyl) ester | Transferase | 7.2 | Cytosol [GO:0005829] |
| **3fnu** | 3 | 6 | 6 | Hydrolase/hydrolase inhibitor | | Food vacuole [GO:0020020] |
| **3jqr** | 2.3 | FAD | Flavin-adenine dinucleotide | Oxidoreductase | 7.1 ^62^ | Apicoplast [GO:0020011] |
| **3llt** | 2.5 | ANP | Phosphoaminophosphonic acid-adenylate ester | Transferase | 7.2 | Nucleus [GO:0005634] |
| **3nie** | 2.3 | ANP | Phosphoaminophosphonic acid-adenylate ester | Transferase | 7.2 | Cytoplasm [GO:0005737]; nucleus [GO:0005634] |
| **3o8a** | 2.3 | O8A | N-cyclopropyl-5-[2-methyl-5-(trifluoromethoxy)-1H-benzimidazol-1-yl]thiophene-2-carboxamide | Oxidoreductase | 8 | Integral component of membrane [GO:0016021]; mitochondrial inner membrane [GO:0005743]; mitochondrion [GO:0005739] |
| **3peh** | 2.75 | IBD | 2-amino-4-{2,4-dichloro-5-[2-(diethylamino)ethoxy]phenyl}-N-ethylthieno[2,3-d]pyrimidine-6-carboxamide | Chaperone |  | Endoplasmic reticulum [GO:0005783]; perinuclear region of cytoplasm [GO:0048471] |
| **3qvi** | 2.5 | K95 | (4R)-N-[(1S,2R)-2-hydroxy-2,3-dihydro-1H-inden-1-yl]-3-[(2S,3S)-2-hydroxy-3-{[S-methyl-N-(phenylacetyl)-L-cysteinyl]amino}-4-phenylbutanoyl]-5,5-dimethyl-1,3-thiazolidine-4-carboxamide | Hydrolase/hydrolase inhibitor 5.5^63^ | | Integral component of membrane [GO:0016021] |
| **3t64** | 1.65 | DU3 | 2',5'-dideoxy-5'-[(diphenylmethyl)amino]uridine | Hydrolase/hydrolase inhibitor | | Parasitophorous vacuole |
| **3tlx** | 2.75 | ADP | Adenosine-5'-diphosphate | Transferase | 7.2; < 6 | Cytosol and inner mitochondrial membrane |
| **3uj8** | 1.35 | SFG | Sinefungin | Transferase/transferase inhibitor | 6 | Golgi apparatus |
| **3uow** | 2.72 | XMP | Xanthosine-5'-monophosphate | Ligase | 7.2 | Cytosol [GO:0005829] |
| **3vi2** | 2.1 | HMZ | 4-(2-hydroxy-4-methoxyphenyl)-4-oxobutanoic acid | Lyase/lyase inhibitor | 7.2 | Cytoplasm |
| **4j56** | 2.37 | FAD | Flavin-adenine dinucleotide | Oxidoreductase | 7.2;8 | Cytoplasm [GO:0005737]; mitochondrion [GO:0005739] |
| **4mvf** | 2 | STU | Staurosporine | Transferase/transferase inhibitor | | Parasitophorous vacuole and the tubovesicular system |
| **4nbj** | 2.2 | D3Y | 3'-deoxy-3'-(D-tyrosylamino)adenosine | Hydrolase | 7.2 | Cytoplasm [GO:0005737] |
| **4py6** | 2.5 | R78 | 4-{[(7R)-8-cyclopentyl-7-ethyl-5-methyl-6-oxo-5,6,7,8-tetrahydropteridin-2-yl]amino}-3-methoxy-N-(1-methylpiperidin-4-yl)benzamide | Protein binding | | Nua4 histone acetyltransferase complex [GO:0035267] |
| **4qt3** | 1.4 | RAP | Rapamycin immunosuppressant drug | Isomerase | 7.2 | Cytoplasm [GO:0005737]; nucleus [GO:0005634] |
| **4gae** | 2.3 | NDP | Nadph dihydro-nicotinamide-adenine-dinucleotide phosphate | Isomerase/isomerase inhibitor | 7.1 ^62^ | Apicoplast [GO:0020011] |

The subcellular locations were primarily retrieved using gene ontology annotation from Uniprot^64^ and secondarily from literature as indicated by the references. It is noteworthy that the experimental indication of a proteins localization may not be exclusive.

## In vitro assays

- pLDH (Malaria) *in vitro* assays: Single Concentration Screen

Figure S36 The bar graph show the % parasite viability ±SD obtained for the individual compounds. Table 4 shows the corresponding DrugBank IDs of the compounds IDs in the assays.

Table 4 Compounds' IDs in the assays and their corresponding DrugBank IDs

| Compounds' IDs in the assay | DrugBank IDs |
| --- | --- |
| BD151900 | DB00649 |
| BD31776 | DB00496 |
| BD21906 | DB01162 |
| BD152555 | DB00853 |
| BD152573 | DB00469 |
| T1203 | DB01203 |
| T0798 | DB00620 |
| T1267 | DB01048 |
| T1139 | DB01001 |
| T1050 | DB00457 |
| T0483 | DB00489 |
| T3043 | DB08877 |
| T2539 | DB08868 |
| T0680 | DB00555 |
| T6216 | DB05812 |
| T0084 | DB01171 |


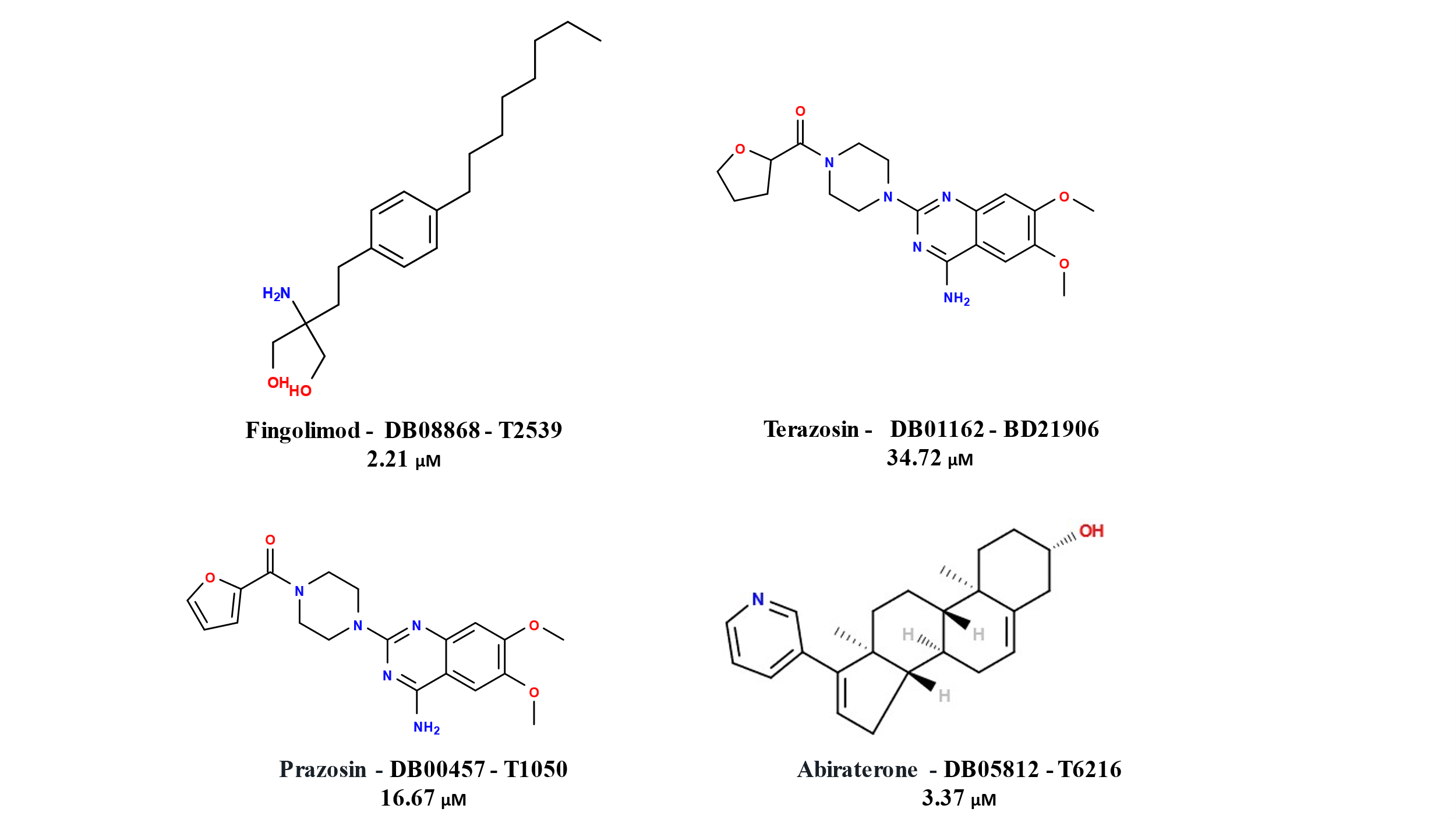


Figure S37 Hits structures with their potency, identifiers and names.


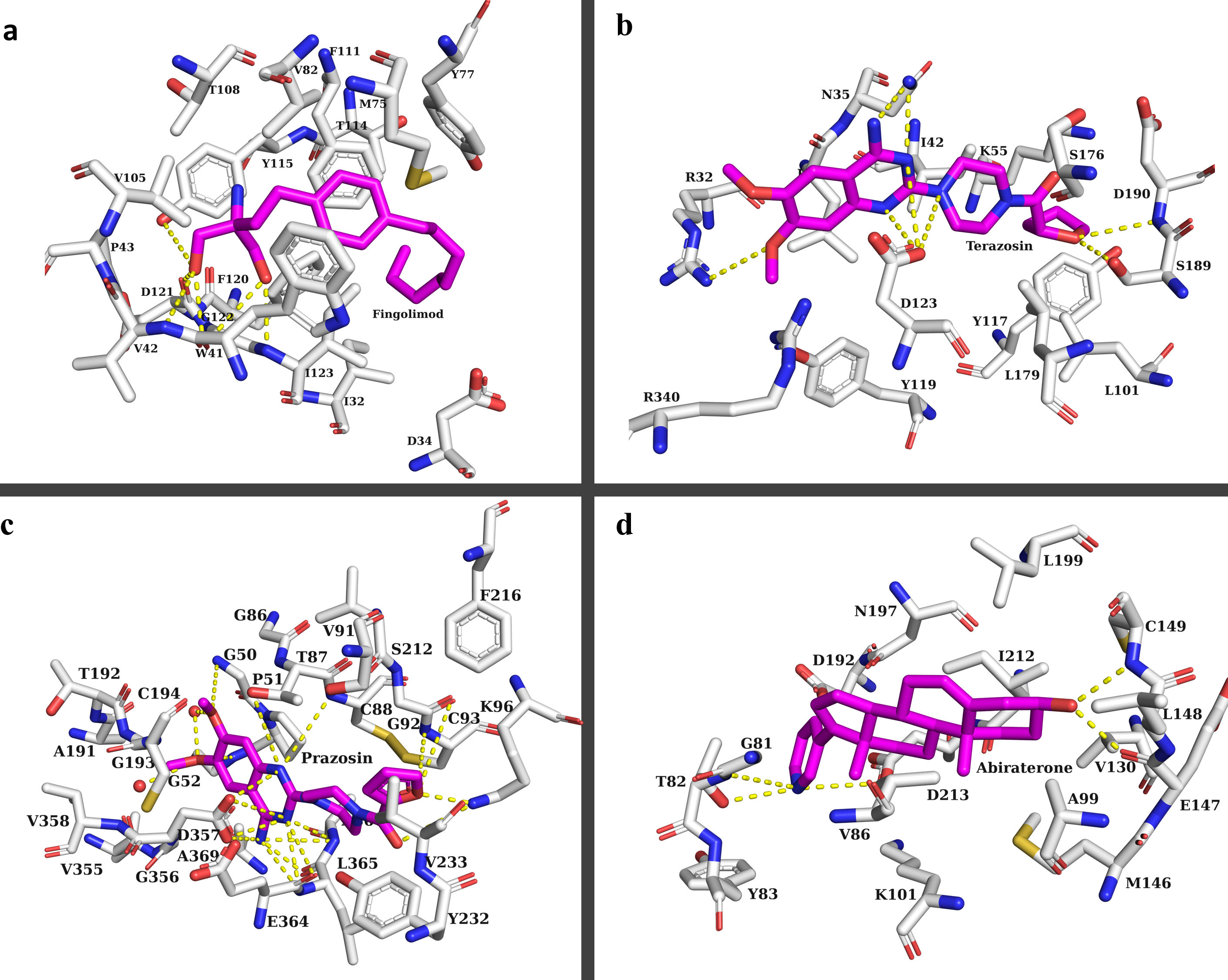


Figure S38 Active compounds binding poses. A) fingolimod, b) terazosin, c) prazosin, d) Abiraterone. The active compound is in magenta. Residues in a radius of 3.5 Ångströms are displayed in while (carbon) and atom types (other elements). They are labelled with their one-letter code and their residue numbers. Polar contacts are displayed in dashed yellow lines.


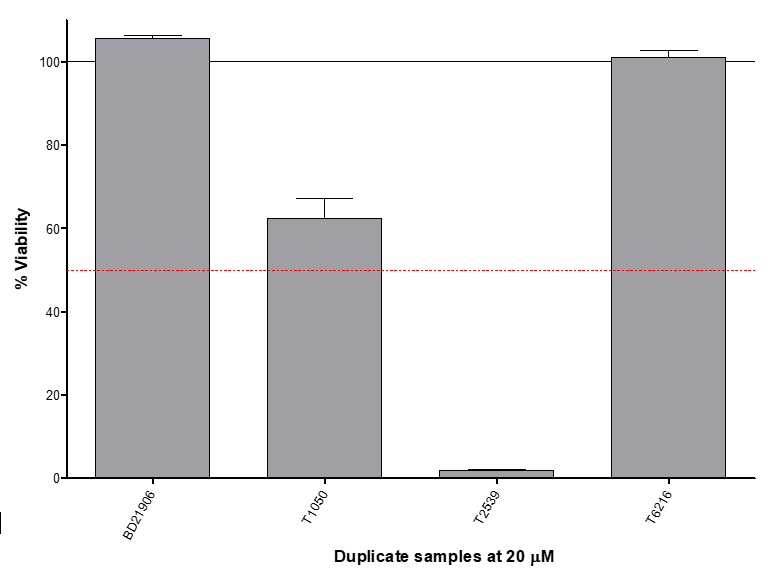


Figure S39 The bar graph show the % human cells viability ±SD obtained for the individual compounds. BD21906, T1050, T2539, T6216 correspond to the following compounds terazosin (DB01162), prazosin (DB00457), fingolimod (DB08868) and abiraterone (DB05812) respectively.


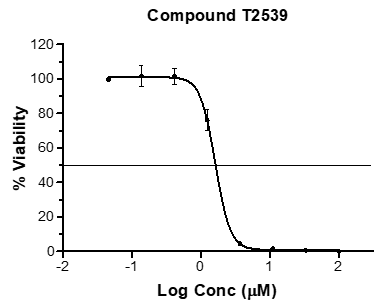


Figure S40 Dose-response plots. Human cell viability percentage is plotted against the Log (compound concentration). The IC50 (50% inhibitory concentration) is obtained from the curve by non-linear regression. Compounds are tested in triplicate with the standard deviation (SD) indicated by the error bars. T2539 corresponds to the following compound fingolimod (DB08868).

1. References

1. Waskom, M. *et al.* mwaskom/seaborn: v0.9.0 (July 2018). (2018) doi:10.5281/ZENODO.1313201.

2. Boss, C. *et al.* Achiral, cheap, and potent inhibitors of plasmepsins I, II, and IV. *ChemMedChem* **1**, 1341–1345 (2006).

3. Barratt, E. *et al.* Thermodynamic Penalty Arising from Burial of a Ligand Polar Group Within a Hydrophobic Pocket of a Protein Receptor. *J. Mol. Biol.* **362**, 994–1003 (2006).

4. Friedman, R. & Caflisch, A. Discovery of Plasmepsin Inhibitors by Fragment-Based Docking and Consensus Scoring. *ChemMedChem* **4**, 1317–1326 (2009).

5. Bhaumik, P. *et al.* Crystal Structures of the Histo-Aspartic Protease (HAP) from Plasmodium falciparum. *J. Mol. Biol.* **388**, 520–540 (2009).

6. Mendez, D. *et al.* ChEMBL: towards direct deposition of bioassay data. *Nucleic Acids Res.* **47**, D930–D940 (2019).

7. Chen, X., Ji, Z. L. & Chen, Y. Z. TTD: Therapeutic Target Database. *Nucleic Acids Res.* **30**, 412–415 (2002).

8. Llanos-Cuentas, A. *et al.* Antimalarial activity of single-dose DSM265, a novel plasmodium dihydroorotate dehydrogenase inhibitor, in patients with uncomplicated Plasmodium falciparum or Plasmodium vivax malaria infection: a proof-of-concept, open-label, phase 2a study. *Lancet. Infect. Dis.* **18**, 874–883 (2018).

9. Booker, M. L. *et al.* Novel inhibitors of Plasmodium falciparum dihydroorotate dehydrogenase with anti-malarial activity in the mouse model. *J. Biol. Chem.* **285**, 33054–64 (2010).

10. Ancelin, M. L., Calas, M., Bonhoure, A., Herbute, S. & Vial, H. J. In vivo antimalarial activities of mono- and bis quaternary ammonium salts interfering with Plasmodium phospholipid metabolism. *Antimicrob. Agents Chemother.* **47**, 2598–605 (2003).

11. Prado-Prado, F. J., García-Mera, X. & González-Díaz, H. Multi-target spectral moment QSAR versus ANN for antiparasitic drugs against different parasite species. *Bioorg. Med. Chem.* **18**, 2225–2231 (2010).

12. Witola, W. H., Pessi, G., El Bissati, K., Reynolds, J. M. & Mamoun, C. Ben. Localization of the phosphoethanolamine methyltransferase of the human malaria parasite Plasmodium falciparum to the Golgi apparatus. *J. Biol. Chem.* **281**, 21305–11 (2006).

13. Aoyama, C., Liao, H. & Ishidate, K. Structure and function of choline kinase isoforms in mammalian cells. *Prog. Lipid Res.* **43**, 266–281 (2004).

14. Serrán-Aguilera, L. *et al.* Plasmodium falciparum Choline Kinase Inhibition Leads to a Major Decrease in Phosphatidylethanolamine Causing Parasite Death. *Sci. Rep.* **6**, 33189 (2016).

15. Chattopadhyay, D. *et al.* Structure of the nucleotide-binding domain of *Plasmodium falciparum* Rab6 in the GDP-bound form. *Acta Crystallogr. Sect. D Biol. Crystallogr.* **56**, 937–944 (2000).

16. Wishart, D. S. *et al.* DrugBank 5.0: a major update to the DrugBank database for 2018. *Nucleic Acids Res.* **46**, D1074–D1082 (2018).

17. Milani, M. *et al.* Ferredoxin-NADP+ Reductase from Plasmodium falciparum Undergoes NADP+-dependent Dimerization and Inactivation: Functional and Crystallographic Analysis. *J. Mol. Biol.* **367**, 501–513 (2007).

18. Mahmoudi, N. *et al.* In vitro activities of 25 quinolones and fluoroquinolones against liver and blood stage Plasmodium spp. *Antimicrob. Agents Chemother.* **47**, 2636–9 (2003).

19. Dubar, F. *et al.* Enhancement of the Antimalarial Activity of Ciprofloxacin Using a Double Prodrug/Bioorganometallic Approach. *J. Med. Chem.* **52**, 7954–7957 (2009).

20. Chandra Shekhar, A., Shanthan Rao, P., Narsaiah, B., Allanki, A. D. & Sijwali, P. S. Emergence of pyrido quinoxalines as new family of antimalarial agents. *Eur. J. Med. Chem.* **77**, 280–287 (2014).

21. Rodrigues, T., Prudêncio, M., Moreira, R., Mota, M. M. & Lopes, F. Targeting the Liver Stage of Malaria Parasites: A Yet Unmet Goal. *J. Med. Chem.* **55**, 995–1012 (2012).

22. Ebstie, Y. A., Abay, S. M., Tadesse, W. T. & Ejigu, D. A. Tafenoquine and its potential in the treatment and relapse prevention of Plasmodium vivax malaria: the evidence to date. *Drug Des. Devel. Ther.* **10**, 2387–99 (2016).

23. Fritz-Wolf, K. *et al.* Crystal Structure of the Plasmodium falciparum Thioredoxin Reductase–Thioredoxin Complex. *J. Mol. Biol.* **425**, 3446–3460 (2013).

24. Derbyshire, E. R., Prudêncio, M., Mota, M. M. & Clardy, J. Liver-stage malaria parasites vulnerable to diverse chemical scaffolds. *Proc. Natl. Acad. Sci. U. S. A.* **109**, 8511–8516 (2012).

25. Kim, S. *et al.* PubChem 2019 update: improved access to chemical data. *Nucleic Acids Res.* **47**, D1102–D1109 (2019).

26. Holton, S. *et al.* Structures of P. falciparum PfPK5 test the CDK regulation paradigm and suggest mechanisms of small molecule inhibition. *Structure* **11**, 1329–1337 (2003).

27. Zhang, H. *et al.* *Stage-specific transcriptome of the malaria parasite in the red blood cell cycle*. vol. 0 https://pdfs.semanticscholar.org/f0d4/cb1f3ccc8a95832b5914dd3d89e91f0b3281.pdf.

28. Cui, H. *et al.* Synthesis and evaluation of α-thymidine analogues as novel antimalarials. *J. Med. Chem.* **55**, 10948–10957 (2012).

29. Färber, P. M., Graeser, R., Franklin, R. M. & Kappes, B. Molecular cloning and characterization of a second calcium-dependent protein kinase of Plasmodium falciparum. *Mol. Biochem. Parasitol.* **87**, 211–216 (1997).

30. Tewari, R. *et al.* The systematic functional analysis of Plasmodium protein kinases identifies essential regulators of mosquito transmission. *Cell Host Microbe* **8**, 377–87 (2010).

31. Josling, G. A. *et al.* A Plasmodium Falciparum Bromodomain Protein Regulates Invasion Gene Expression. *Cell Host Microbe* **17**, 741–751 (2015).

32. Manhas, A., Kumar, S. P. & Jha, P. C. Molecular modeling of Plasmodium falciparum peptide deformylase and structure-based pharmacophore screening for inhibitors. *RSC Adv.* **6**, 29466–29485 (2016).

33. Qidwai, T., Jamal, F., Khan, M. Y. & Sharma, B. Exploring Drug Targets in Isoprenoid Biosynthetic Pathway for Plasmodium falciparum. *Biochem. Res. Int.* **2014**, (2014).

34. Mishra, M., Mishra, V. K., Kashaw, V., Iyer, A. K. & Kashaw, S. K. Comprehensive review on various strategies for antimalarial drug discovery. *European Journal of Medicinal Chemistry* vol. 125 1300–1320 (2017).

35. Lenz, R. & Zenk, M. H. Purification and Properties of Codeinone Reductase (NADPH) from Papaver somniferum Cell Cultures and Differentiated Plants. *Eur. J. Biochem.* **233**, 132–139 (1995).

36. Masini, T., Kroezen, B. S. & Hirsch, A. K. H. Druggability of the enzymes of the non-mevalonate-pathway. *Drug Discov. Today* **18**, 1256–1262 (2013).

37. Perbandt, M., Burmeister, C., Walter, R. D., Betzel, C. & Liebau, E. Native and Inhibited Structure of a Mu class-related Glutathione S-transferase from Plasmodium falciparum. *J. Biol. Chem.* **279**, 1336–1342 (2004).

38. Quesada-Soriano, I., Barón, C., García-Maroto, F., Aguilera, A. M. & García-Fuentes, L. Calorimetric studies of ligands binding to glutathione S-transferase from the malarial parasite Plasmodium falciparum. *Biochemistry* **52**, 1980–1989 (2013).

39. Hiller, N. *et al.* Plasmodium falciparum glutathione S -transferase-Structural and mechanistic studies on ligand binding and enzyme inhibition . *Protein Sci.* **15**, 281–289 (2006).

40. Ahmad, S. *et al.* Mechanism of chiral proofreading during translation of the genetic code. *Elife* **2013**, e01519 (2013).

41. Bhatt, T. K., Soni, R. & Sharma, D. Recent updates on DTD (D-Tyr-tRNA Tyr Deacylase): An enzyme essential for fidelity and quality of protein synthesis. *Front. Cell Dev. Biol.* **4**, 32 (2016).

42. Dufe, V. T. *et al.* Crystal Structure of Plasmodium falciparum Spermidine Synthase in Complex with the Substrate Decarboxylated S-adenosylmethionine and the Potent Inhibitors 4MCHA and AdoDATO. *J. Mol. Biol.* **373**, 167–177 (2007).

43. Wang, J. *et al.* Haem-activated promiscuous targeting of artemisinin in Plasmodium falciparum. *Nat. Commun.* **6**, 10111 (2015).

44. Bhaumik, P. *et al.* Structural insights into the activation and inhibition of histo-aspartic protease from Plasmodium falciparum. *Biochemistry* **50**, 8862–79 (2011).

45. Freundlich, J. S. *et al.* Synthesis, biological activity, and X-ray crystal structural analysis of diaryl ether inhibitors of malarial enoyl acyl carrier protein reductase. Part 1: 4′-Substituted triclosan derivatives. *Bioorg. Med. Chem. Lett.* **15**, 5247–5252 (2005).

46. Samal, R. P. *et al.* Design, Synthesis, Structural Characterization by IR, ^1^ H, ^13^ C, ^15^ N, 2D-NMR, X-Ray Diffraction and Evaluation of a New Class of Phenylaminoacetic Acid Benzylidene Hydrazines as *pf* ENR Inhibitors. *Chem. Biol. Drug Des.* **81**, 715–729 (2013).

47. Lo, E. J. *et al.* DrugBank 5.0: a major update to the DrugBank database for 2018. *Nucleic Acids Res.* **46**, D1074–D1082 (2017).

48. Bilsland, E. *et al.* Plasmodium dihydrofolate reductase is a second enzyme target for the antimalarial action of triclosan. *Sci. Rep.* **8**, 1038 (2018).

49. Trager, W. *et al.* Human malaria parasites in continuous culture. *Science (80-. ).* **193**, 673–675 (1976).

50. Plouffe, D. *et al.* In silico activity profiling reveals the mechanism of action of antimalarials discovered in a high-throughput screen. *Proc. Natl. Acad. Sci. U. S. A.* **105**, 9059–9064 (2008).

51. Chaudhary, K. *et al.* Purine Salvage Pathways in the Apicomplexan Parasite Toxoplasma gondii* □ S Downloaded from. *J. Biol. Chem.* **279**, 31221–31227 (2004).

52. Eaazhisai, K. *et al.* Crystal Structure of Fully Ligated Adenylosuccinate Synthetase from Plasmodium falciparum. *J. Mol. Biol.* **335**, 1251–1264 (2004).

53. Liu, T., Lin, Y., Wen, X., Jorissen, R. N. & Gilson, M. K. BindingDB: a web-accessible database of experimentally determined protein-ligand binding affinities. *Nucleic Acids Res.* **35**, D198–D201 (2007).

54. Sumathy, K., Jayalakshmi, R., Shivayogi, M. S. & Balaram, H. Cloning and characterization of the Plasmodium falciparum adenylosuccinate synthetase gene. *Current Science* vol. 78 610–615 (2000).

55. Conners, R. *et al.* Mapping the binding site for gossypol-like inhibitors of Plasmodium falciparum lactate dehydrogenase. *Mol. Biochem. Parasitol.* **142**, 137–148 (2005).

56. Jaijyan, D. K., Verma, P. K. & Singh, A. P. A novel FIKK kinase regulates the development of mosquito and liver stages of the malaria. *Sci. Rep.* **6**, 39285 (2016).

57. Hassan, N. M., Alhossary, A. A., Mu, Y. & Kwoh, C. K. Protein-Ligand Blind Docking Using QuickVina-W with Inter-Process Spatio-Temporal Integration. *Sci. Rep.* **7**, 15451 (2017).

58. Wójcikowski, M., Ballester, P. J. & Siedlecki, P. Performance of machine-learning scoring functions in structure-based virtual screening. *Sci. Rep.* **7**, 46710 (2017).

59. Desaphy, J., Raimbaud, E., Ducrot, P. & Rognan, D. Encoding protein-ligand interaction patterns in fingerprints and graphs. *J. Chem. Inf. Model.* **53**, 623–637 (2013).

60. Vigers, G. P. A. & Rizzi, J. P. Multiple Active Site Corrections for Docking and Virtual Screening. *J. Med. Chem.* **47**, 80–89 (2004).

61. Theillet, F. X. *et al.* Physicochemical properties of cells and their effects on intrinsically disordered proteins (IDPs). *Chemical Reviews* vol. 114 6661–6714 (2014).

62. Mohring, F. *et al.* Determination of glutathione redox potential and pH value in subcellular compartments of malaria parasites. *Free Radic. Biol. Med.* **104**, 104–117 (2017).

63. Nasamu, A. S., Polino, A. J., Istvan, E. S. & Goldberg, D. E. Malaria parasite plasmepsins: More than just plain old degradative pepsins. *J. Biol. Chem.* **295**, 8425–8441 (2020).

64. Bateman, A. *et al.* UniProt: The universal protein knowledgebase. *Nucleic Acids Res.* **45**, D158–D169 (2017).
